# Genome-Wide Identification and Expression Pattern of the *ANK* Gene Family in *Sorghum bicolor* Under Salt Stress

**DOI:** 10.3390/ijms27177810

**Published:** 2026-08-31

**Authors:** Cuijie Cui, Xueer Chen, JiaHui Wang, Qihuan Yao, Chao Wang, Shangfu Ren, Kun Xie

**Affiliations:** Key Laboratory of Biological Resources and Ecology of Pamirs Plateau in Xinjiang Uygur Autonomous Region, College of Life and Geographic Sciences, Kashi University, Kashi 844000, China; ccj19987654321@163.com (C.C.); 17395592308@163.com (X.C.); 19999797307@163.com (J.W.); yaoqihuana@163.com (Q.Y.); 13579774188@163.com (C.W.); renshangfu@163.com (S.R.)

**Keywords:** *Sorghum bicolor* L., *ANK* gene family, bioinformatics analysis, expression pattern, salt stress

## Abstract

The Ankyrin-repeat proteins (ANKs) play a key role in plant development and in response to abiotic stress. This research identified family members of the ANK genes in *Sorghum bicolor* at the whole-genome level, analyzed their sequence characteristics, evolutionary relationships, and expression patterns, and provided a scientific basis for elucidating the functionality of *SbANK* genes and for salt-tolerant breeding. Using bioinformatics methods, this study conducted a comprehensive identification of the *SbANK* gene family, analyzing its physicochemical properties, domain composition, chromosomal distribution, colinearity relationships, promoter cis-acting elements, and conserved protein motifs. Transcriptomic data and qRT-PCR were used to detect changes in their expression under salt stress. A total of 186 *ANK* family members were identified in the *Sorghum bicolor* genome, classified into 13 subfamilies and unevenly distributed across 10 chromosomes. Intra-species colinearity analysis revealed 7 pairs of duplicated genes, while inter-species colinearity analysis showed that *S. bicolor* and *Oryza sativa* share 88 pairs of orthologs, far exceeding the number found in *Arabidopsis thaliana* (11 pairs). Promoter analysis indicated that *SbANK* genes are enriched with cis-acting elements associated with hormone responses (particularly MeJA elements, accounting for 51.7%) and stress responses (particularly anaerobic-inducible elements, accounting for 60.9%). Transcriptomic expression analysis revealed that *SbANK* genes exhibit distinct tissue specificity, with the ANK-IQ subfamily highly expressed in leaves and the ANK-M subfamily showing the most widespread response under salt stress. Expression levels of the 10 candidate genes showing the most significant responses to salt stress were analyzed using qRT-PCR. The results indicated that *SbANK91*, *SbANK135*, and *SbANK136* were significantly upregulated under 200 mmol/L NaCl treatment. The *SbANK* family is distinguished by a large number of member genes and structural diversity, with the ANK-M subfamily being the primary group responding to salt stress. *SbANK91*, *SbANK135*, and *SbANK136* are identified as putative candidate genes for salt stress responses.

## 1. Introduction

*Sorghum bicolor* is a C4 monocot in the Poaceae family. Due to its strong photosynthetic capacity and wide adaptability, it is one of China’s five major grain crops. It is cultivated in arid and semi-arid regions worldwide and is used in various industries, including brewing, livestock feed, and sugar production [1]. In recent years, with the global population continuing to grow and environmental degradation accelerating rapidly, *S. bicolor* has attracted widespread attention from breeders as a high-quality energy crop due to its exceptional drought, cold, and salt-alkali tolerance [2,3,4]. *S. bicolor* is a diploid plant with a genome size of approximately 750 Mb. In 2009, the complete genome sequencing of *S. bicolor* was successfully completed, providing a crucial foundation for the study of *S. bicolor* gene families [5].

Ankyrin-repeat proteins (ANKs) are a class of protein structures widely found in living organisms. ANKs were initially identified in the yeast cell-cycle regulating factors Swi6/Cdc10 and in Drosophila melanogaster signaling proteins [6]; subsequently, a 24-amino acid ANK sequence was characterized in the cytoskeleton [7]. A typical ANK protein repeat consists of 33 amino acid residues. The ANK motifs fold into a β2α2 structure within the ANK domain, forming an L-shape in space. A variable number of ANK units interact via hydrogen bonds and hydrophobic interactions to form a compact, stable protein domain [8,9]. Most ANK domains mediate protein–protein interactions [10], can bind to a variety of ligands, and are found in proteins with diverse functions [11,12].

Proteins containing ANK repeats are widely distributed, ranging from viruses, prokaryotes, and eukaryotes to humans; they can be found in both the cytoplasm and the extracellular matrix. These proteins are numerous and form a large family, which primarily includes CDK (cyclin-dependent kinase) inhibitors, transcription regulators, cytoskeletal organizers, developmental regulators, membrane proteins, and toxins, among others [13]. In plants, current research on ANK proteins is primarily focused on the model plant *Arabidopsis thaliana*. The first ANK protein-coding gene identified in *A*. *thaliana* is AKR, which is regulated by light signals and plays a regulatory role in cell differentiation and growth [14]. Subsequently, the functions of an increasing number of plant ANK proteins have been reported. In *A. thaliana*, the ANK protein BOPI is involved in leaf morphogenesis [15]; *A. thaliana* EMB506 contains five ANK domains and plays a crucial role in embryonic development [16]; *A*. *thaliana* XBAT32, which encodes a ubiquitin ligase, is involved in lateral root formation [17]; and the ANK protein LINK in the genus *Lilium* plays a key role in pollen tube emergence and growth [18]. Research indicates that ANK proteins are also involved in plant responses to signals from biotic and abiotic stresses. For example, *A. thaliana* AKR2 is implicated in the metabolism of antioxidants during biotic and abiotic response processes [19]; ACD6 and NPR1 are associated with salicylic acid signaling and play important roles in biotic stress responses [20,21,22]; *Oryza sativa* XB3 is involved in XA21-mediated plant immune processes [23]; *Capsicum annuum* KR1 responds to both biotic and abiotic stress processes [24]; and *Gossypium hirsutum* ANK169 is involved in cotton’s resistance to high-temperature stress [25].

To date, systematic studies of the *ANK* gene family have primarily focused upon the reference plants *A. thaliana* and *O. sativa*, while research on *ANK* genes in other species, particularly grasses, remains in its infancy. There is currently a severe lack of research on the role of ANK in the development of *S. bicolor* and its resilience to abiotic stress. The identification and analysis of *ANK* genes in grass genomes will help refine our understanding of the functional and evolutionary characteristics of this family. This study employed bioinformatics techniques to identify the *SbANK* family members at the genome-wide level. We analyzed the physicochemical properties, phylogenetic relationships, gene structure, conserved motifs, and expression patterns of the SbANK proteins, with the aim of providing a reference for research into the functions of the *SbANK* family of genes.

## 2. Results

### 2.1. Identification of the S. bicolor ANK Gene Family

Using the HMMER software (V3.4), a combined total of 186 *ANK* family members were characterized in the *S. bicolor* genome and named *SbANK1* through *SbANK186*. Key characteristics of the *SbANK* gene family—including coding sequence length, molecular weight, theoretical isoelectric point, instability index, aliphatic index, hydrophilicity, and subcellular localization—are summarized in Table S1. The number of amino acids ranges from 90 (SbANK143) to 1967 (SbANK99); molecular weights range from 9.85 kDa (SbANK143) to 177.98 kDa (SbANK99); theoretical isoelectric points range from 4.3 (SbANK41) to 10.05 (SbANK140), with 70 members having a theoretical isoelectric point greater than 7, indicating basicity; 116 members have a theoretical isoelectric point below 7, indicating acidity; the instability index ranges from 19.16 (SbANK35) to 65.74 (SbANK66); additionally, the aliphaticity index ranges from 64.05 (SbANK171) to 112.41 (SbANK147), reflecting differences in thermal stability within the SbANK family.

Most SbANK proteins have negative GRAVY (Global Average Hydrophilicity) scores, indicating that the sorghum ANK family is predominantly hydrophilic; however, 40 members exhibit positive GRAVY scores, suggesting that they are hydrophobic. Subcellular localization predictions indicate that most SbANK members are localized to the nucleus, cytoplasm, plasma membrane, and chloroplasts, while a small number are predicted to be localized to peroxisomes, mitochondria, the cytoskeleton, or the endoplasmic reticulum. Domain analysis reveals that 96 SbANK proteins contain only ANK repeats, while most other SbANK proteins contain multiple domains. Based on differences in protein domains, SbANK proteins were classified into 13 categories (Table S2), including 100 members of the ANK-M subfamily, which contains only the ANK domain. In addition to the ANK domain, other domains with known functions were identified, including 7 members of the ANK-TM subfamily that contain transmembrane domains. The 14 members of the ANK-RF subfamily contain RING finger domains. Eight members of the ANK-BTB subfamily contain BR-C, TTK, and BAB domains. Three members of the ANK-BPA subfamily contain BAR, PH, and ArfGAP structural motifs. The 20 members of the ANK-TPR subfamily contain a tetrahedral tetrapeptide. The ANK-ZF subfamily has 5 members containing zinc-finger domains. The 11 members of the ANK-IQ subfamily contain calmodulin-binding domains. The ANK-MS subfamily has 3 members containing Motile_Sperm domains. The ANK-GPCR family has 1 member containing a GPCR chaperone domain. The ANK-O family has 3 members containing other domains, including ANK-Chromo, ANK-AAA, and ANK-Pellino. The ANK-SBP family has 1 member containing an SBP domain. The ANK-IT family has 10 members containing potassium channel proteins.

### 2.2. Phylogenetic Tree Analysis of the ANK Gene Family in S. bicolor

**Figure 1:**
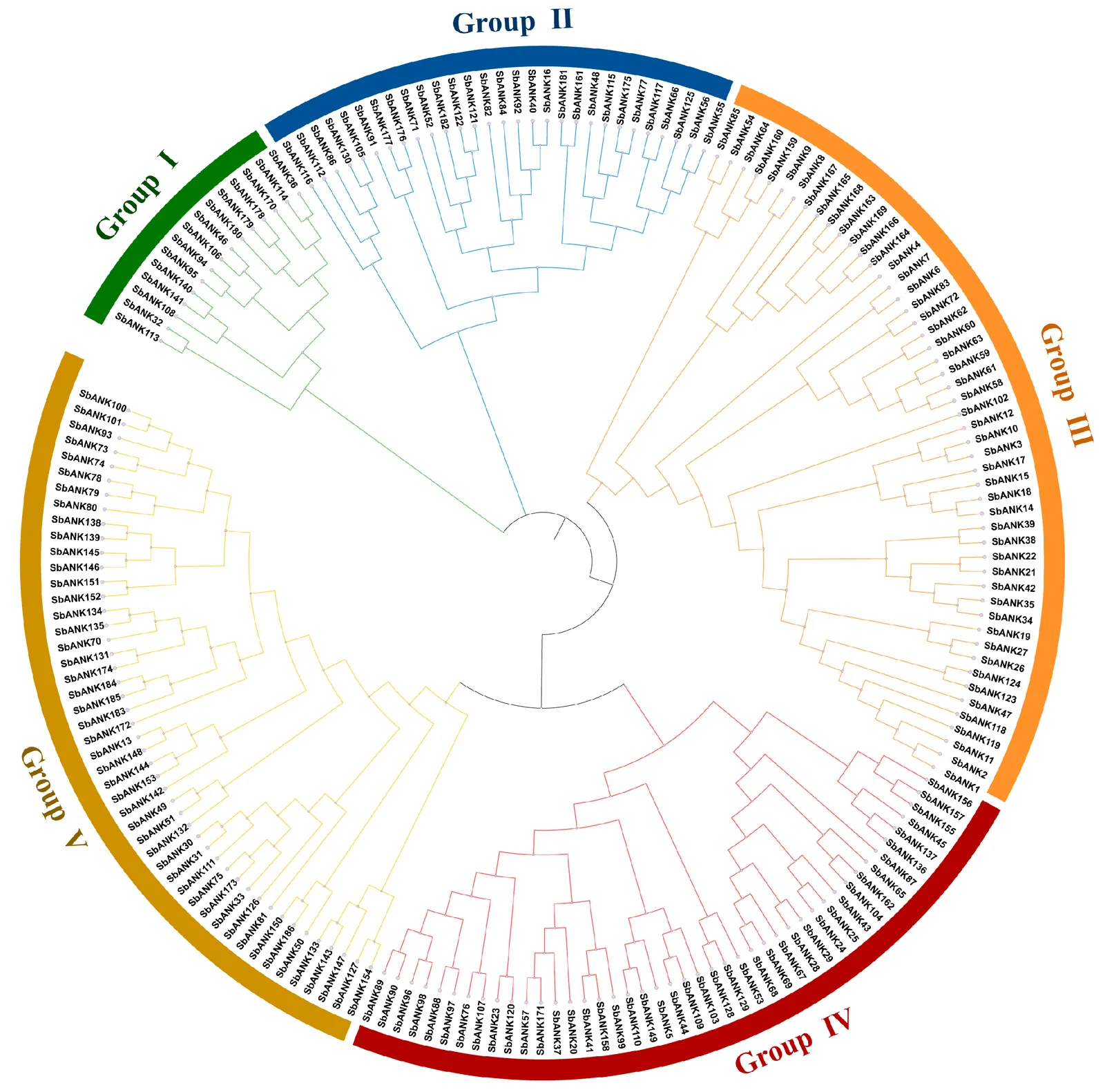
A phylogenetic tree of the *SbANK* gene family was constructed by employing the maximum likelihood method and performing 1000 bootstrap repetitions.

To elucidate phylogenetic relationships within the *SbANK* gene family, we reconstructed a phylogenetic tree using the maximum likelihood method (bootstrap = 1000) with the full-length sequences from the SbANK proteins (Table S2). Based on these evolutionary relationships, we classified the 186 *SbANK* genes into five groups (Groups I–V), containing 15, 29, 51, 44, and 47 members, respectively (Figure 1). In the phylogenetic tree, some closely related genes belong to different subfamilies. For example, in Group III (which includes multiple subfamilies such as ANK-M, ANK-O, ANK-RF, ANK-TPR, and ANK-BTB), *SbANK160* and *SbANK159* are adjacent on the tree; however, *SbANK160* is classified as belonging to the ANK-M subfamily, while *SbANK159* belongs to the ANK-ZF subfamily. Similarly, *SbANK66* and *SbANK117* in Group II are closely related but are classified into the ANK-GPCR and ANK-M subclades, respectively. The clustering outcomes of the phylogenetic tree primarily reflect the overall similarity of full-length sequences, whereas subfamily classification relies more heavily on the composition of specific conserved domains. Therefore, genes that are phylogenetically close but have different domain compositions may still be adjacent on the tree, reflecting the complex relationship between domain rearrangement and sequence conservation within the *ANK* gene family during evolution.

### 2.3. Genetic Structure of the ANK Gene Family in S. bicolor

To investigate structural similarities and differences among members of the *SbANK* family, we analyzed the composition of conserved motifs in the family’s proteins as well as the exon-intron structures of the corresponding genes. Within the 186 SbANK proteins, a combined total of six conserved motifs (Motifs 1–6) were characterized (Figure S1A). The composition and arrangement of these motifs showed significant differentiation across different subfamilies. In particular, motif 4 is present in the majority of members across all subfamilies and is the core conserved motif of the SbANK protein family, suggesting that it plays a key role in sustaining the fundamental structural and functional stability of SbANK proteins. It is worth noting that certain members of this gene family lack Motif 4; among those lacking Motif 4, the majority contain only Motif 1 and Motif 3, exhibiting distinct structural simplification (e.g., SbANK5, SbANK41, SbANK52, SbANK55, SbANK56, SbANK57, SbANK64, SbANK84, SbANK85, SbANK91, SbANK107, SbANK115, SbANK120, SbANK125, SbANK149, SbANK159, SbANK160, SbANK161, SbANK176, SbANK177, SbANK181). According to the results of the above analysis, we speculate that members lacking Motif 4 may have undergone structural loss and functional diversification during evolution.

Analysis of gene structure indicates that *SbANK* family genes contain between 2 and 20 exons, and there are significant differences in exon-intron structure among different family members (Figure S1B). This degree of variation suggests that family members may no longer perform identical functions and have diverged in terms of tissue specificity, spatiotemporal expression, and biochemical functions. Furthermore, among the 186 *SbANK* genes, 29 lacked detectable UTR structures, indicating structural incompleteness. In summary, this family includes both structurally intact, conventionally post-transcriptionally regulated genes and a group of structurally simplified or incompletely annotated members, reflecting the complexity of the *SbANK* gene family in terms of evolution and functional differentiation.

### 2.4. Chromosomal Location and Synteny Analysis

To clarify the genomic patterns of the *SbANK* gene family, we analyzed the localization of 186 *SbANK* genes across 10 chromosomes. The results indicate that the distribution of *SbANK* genes across the chromosomes is uneven (Figure S2A). Chromosomes 1 and 2 carry the largest number of *ANK* genes (34 each), followed by chromosome 5 (23), chromosomes 4 and 8 (19 each), chromosome 9 (15), chromosome 7 (13), and chromosome 10 (11), with chromosome 3 having the fewest (10). This distribution pattern suggests that the *SbANK* gene family may have experienced unequal amplification events throughout its evolutionary history.

To investigate duplication events within this gene family, we conducted an intraspecific linkage analysis. The results identified seven pairs of linked *SbANK* genes in the sorghum genome (Figure S2B), all of which were segmental duplicates; no tandem duplicates were detected. This suggests that segmental repeats are the key molecular mechanism underlying the expansion and structural diversity of members of the *SbANK* gene family.

To better evaluate the evolutionary conservation of the *SbANK* gene family, we constructed interspecific linkage maps between sorghum and representative species (*A. thaliana* and *O. sativa*) (Figure S2C). The findings revealed that there were only 11 pairs of direct homologous *ANK* genes between *S. bicolor* and *A. thaliana*, whereas 88 pairs of direct homologous genes were detected between *S. bicolor* and *O. sativa*, another member of the Poaceae family, accounting for a significant proportion (approximately 47%) of the sorghum *ANK* gene family. This striking contrast indicates that the level of synteny and conservation within the *SbANK* gene family is significantly higher between the two compared grass species (*S. bicolor* and *O. sativa*) than between *S. bicolor* and the dicotyledonous plant *A. thaliana*, suggesting that this *ANK* gene family may have evolved relatively conservatively within monocotyledons.

### 2.5. Identification of Cis-Acting Regulatory Elements in the ANK Gene Family Promoters of S. bicolor 

Using the PlantCARE database, cis-regulatory elements within the 2000 bp region upstream of the *SbANK* gene promoter were identified. A total of 4772 cis-elements were detected across all *SbANK* promoters (Figure S3). These cis-regulatory elements were classified into four major categories: (1) hormone response elements, (2) stress and defense response elements, (3) growth and development regulation elements, and (4) light response and metabolic regulation elements. Among them, 2056 elements related to light response and metabolic regulation were identified, comprising five subcategories: light response, circadian rhythm, metabolic regulation, MYB binding, and ATBP binding; In addition, 1791 hormone-responsive elements were identified, including: auxin responsiveness, gibberellin responsiveness, salicylic acid responsiveness, MeJA responsiveness, and abscisic acid responsiveness, with MeJA-responsive elements being the most prevalent, accounting for 51.7%; Among the stress and defense response elements, the following were identified: cold response elements, anaerobic induction elements, wounding response elements, defense response elements, and inducer-activated elements. Among these, anaerobic induction elements accounted for the highest proportion at 60.9%, followed by low-temperature responsiveness, accounting for 26.9%. Among the six growth and development regulatory elements, which include meristem expression, endosperm expression, seed-specific regulation, root-specific regulation, differentiation of palisade mesophyll cells, and cell cycle regulation, meristem expression was dominant, accounting for 52.5%. It is worth noting that the distribution of cis-regulatory elements is similar among genes within the same subfamily but differs between members of different subfamilies. For example, in the ANK-IQ subfamily, *SbANK67*, *SbANK68*, and *SbANK69* contain the same types of cis-regulatory elements, including the most abundant environmental stress response motifs. In the ANK-M subfamily, *SbANK58*, *SbANK59*, *SbANK60*, *SbANK61*, *SbANK62*, and *SbANK63* share the same types of cis-regulatory elements, which are rich in hormone response elements. This diverse distribution suggests that *SbANK* genes may have the potential to respond to various environmental signals and developmental processes.

### 2.6. Analysis of the Expression Patterns of ANK Family Genes in S. bicolor

**Figure 2:**
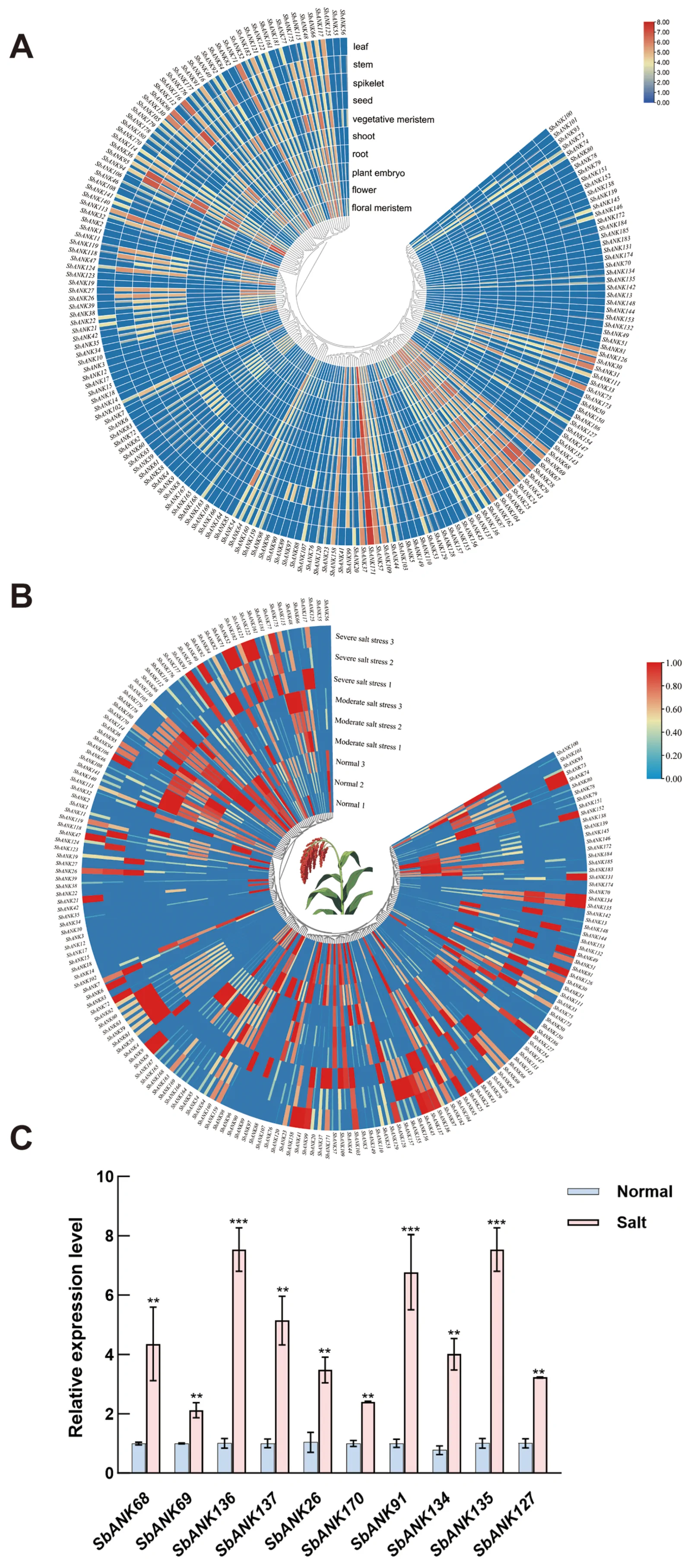
Expression of the *SbANK* gene Family. (**A**) Expression pattern analysis of the *SbANK* gene in different tissues. (**B**) Analysis of the expression patterns of the *SbANK* gene under varying degrees of salt stress. (**C**) Expression of the *SbANK* gene in *S. bicolor* leaves under 200 mmol/L NaCl treatment. Data are presented as the mean (±standard deviation) of three biological replicates. The asterisks above the data bars indicate significant differences (Student’s *t*-tests,** *p* < 0.01 and *** *p* < 0.001) or no significant differences, respectively.

To preliminarily investigate the putative functions of the *SbANK* gene family in *S. bicolor* growth and development, this study utilized transcriptomic data to analyze the expression levels of 186 *SbANK* genes across 10 tissue types. As shown in Figure 2A, most *SbANK* genes exhibit tissue-specific or tissue-preferential expression patterns, and members of the same subfamily often display similar expression characteristics. In the ANK-RF subfamily, *SbANK94* and *SbANK95* exhibited high expression levels (expression values of 5–6) in stems, vegetative meristems, roots, and floral meristems, indicating that both genes may primarily participate in the development of sorghum vegetative organs and the maintenance of meristematic activity. The ANK-IQ subfamily exhibits distinct high expression in leaves: *SbANK24*, *SbANK25*, *SbANK28*, *SbANK29*, *SbANK65*, *SbANK68*, *SbANK69*, and *SbANK162* all exhibit high expression across all tested tissues (expression values 4–8), with leaf expression being the most prominent (expression value 8). This broad-spectrum, high-expression pattern suggests that members of this subfamily may perform fundamental functions in multiple tissues of sorghum. The ANK-M subfamily exhibits unique expression patterns, with the vast majority of members showing generally low expression levels across all tested tissues (expression values 0–2); however, *SbANK171* is a notable exception: this gene exhibits high and uniform expression levels (expression values of 6–8) across all 10 tissues, suggesting that *SbANK171* is a highly expressed gene that likely plays an important role in fundamental cellular processes in sorghum, with a function that differs from that of other, low-expression members of this family. The expression profile described above provides important clues for subsequent functional validation of candidate genes.

To explore the putative roles of *SbANK* gene family members in sorghum’s stress response to salt, this study analyzed the expression changes of 186 *SbANK* genes under normal growth conditions (CK), moderate salt stress (MS), and severe salt stress (SS). The findings revealed that members of different subfamilies exhibited significantly different response patterns to salt stress (Figure 2B). In the ANK-RF subfamily, the expression levels of *SbANK94*, *SbANK95*, and *SbANK106* showed increased expression under moderate salt stress, indicating that these three genes may primarily participate in sorghum’s early-stage response to moderate salinity. In the ANK-IQ subfamily, the expression levels of *SbANK68* and *SbANK69* increased under severe salt stress, while no significant changes were observed under moderate salt stress, suggesting that they may play specific roles in tolerance to severe salt stress. The ANK-M subfamily exhibited the most extensive response to salt stress. Under salt stress conditions, expression levels are upregulated in the vast majority of members of this subfamily. Among these, *SbANK58*, *SbANK59*, *SbANK60*, *SbANK61*, *SbANK62*, and *SbANK63* were all consistently upregulated under both moderate and severe salt stress conditions, whereas *SbANK136*, *SbANK137*, and *SbANK91* showed increases in expression only under severe salt stress. These results indicate that the ANK-M subfamily is the primary *ANK* gene group responding to salt stress in sorghum, and different members may have distinct functional roles in responding to varying levels of stress. 

In summary, the expression of multiple subfamilies within the sorghum *SbANK* gene family (particularly the ANK-M subfamily) is induced by salt stress, indicating that they may be participating in the regulatory network governing sorghum salt tolerance and represent important candidates for future functional characterization of salt-tolerance genes. qRT-PCR validation of the expression of selected *SbANK* genes in sorghum leaves under salt stress treatment showed that, under 200 mmol/L NaCl treatment, the expression profiles of the selected *SbANK* genes under high salt stress were inconsistent with the transcriptomic sequencing data, with all genes showing upregulated expression. Among them, *SbANK91*, *SbANK135*, and *SbANK136* exhibited the most significant changes in expression levels (Figure 2C). *SbANK91*, *SbANK135*, and *SbANK136* were the genes that were most significantly and consistently upregulated under salt stress conditions, indicating that they are key members of the *SbANK* gene family involved in salt stress response and may play important biological roles in sorghum’s defense against salt stress.

## 3. Discussion

ANK proteins are involved in protein–protein interactions, respond to various biotic and abiotic stresses, and regulate plant developmental growth. *ANK* genes are widely distributed in plants, though the types and numbers of ANK proteins vary significantly among species. With the advancement of plant genomics research, the study of gene families has become a key focus in current research on plant gene function. In this study, a search of the sorghum genome identified a family of 186 members of the *ANK* gene family, which were further classified into 13 subfamilies according to their domain structure. This number is comparable to that in *Oryza sativa* (175) [23] but significantly higher than in *Arabidopsis thaliana* (105) [17]. Whether this difference reflects adaptive expansion, lineage-specific retention, or stochastic genomic variation remains unclear. While we consider it plausible that the expanded *ANK* repertoire may contribute to stress adaptation, this remains a speculative hypothesis that requires formal testing through evolutionary analyses such as dN/dS and functional divergence studies.

Previously, research has demonstrated that certain members of the ANK protein family in species such as *A. thaliana* [17], *O. sativa* [23], *Lilium* [18], and *C. annuum* [24] are involved in light signaling regulation, leaf morphogenesis, embryonic development, lateral root formation, and responses to biotic and abiotic stress signals. Given that sorghum contains a larger number of ANK proteins with greater structural diversity than either *O. sativa* or *A. thalian*, we speculate that the expanded ANK repertoire in sorghum may contribute to more complex and diverse physiological functions. As a typical C4 stress-tolerant crop with remarkable adaptability to drought and salinity, sorghum’s large ANK family size may be related to its environmental adaptation requirements.

Structural analysis revealed that 96 SbANK proteins contain only ANK repeats (ANK-M subfamily), whereas other members possess additional domains such as RING, BTB, TPR, and IQ, suggesting functional specialization within the family. ANK-M proteins likely function primarily as scaffold proteins, mediating protein–protein interactions through their ANK repeats. In contrast, ANK-RF members carrying RING finger domains (e.g., SbANK94, SbANK95) may possess E3 ubiquitin ligase activity and participate in protein degradation pathways [26], a function previously confirmed for XBAT32 in Arabidopsis [17] and XB3 in rice [23], both of which regulate lateral root development and immune responses through ubiquitination. Similarly, ANK-TPR and ANK-BTB subfamily members may be involved in protein complex assembly and transcriptional regulation [27]. However, these predictions are inferred from domain architecture and require direct experimental confirmation. Notably, while Motif 4 is conserved in most SbANK proteins, a subset of members lacking this motif (e.g., *SbANK159*, *SbANK160*) retain only Motifs 1 and 3, exhibiting structural simplification. These proteins have rarely been reported in plants and may either mediate specific interactions through shorter ANK units or have undergone functional degeneration during evolution. Additionally, 29 genes lacked detectable UTR structures, which may reflect incomplete genome annotation, although the possibility of genuine atypical gene structures cannot be excluded.

A total of 88 homologous ANK gene pairs were identified between *S. bicolor* and *O. sativa*, compared to only 11 pairs between *S. bicolor* and *A. thaliana*. This finding is highly concordant with the phylogenetic relationships between the species (*S. bicolor* and *O. sativa* belong to the Poaceae family, while *A. thaliana* belongs to the Brassicaceae family), indicating that *ANK* genes are relatively conserved in monocotyledons but have undergone significant diversification in dicotyledons. This result also supports the notion that subsequent functional studies should draw more heavily on existing reports from Poaceae crops (such as *O. sativa*) rather than *A. thaliana*.

Transcriptomic analysis indicates that *SbANK* genes display distinct tissue-specific expression patterns. The ANK-IQ subfamily is highly expressed in leaves (expression value of 8) and shows relatively high expression across all tissues, indicating this subfamily may participate in core functions of the leaf organ, such as photosynthesis and carbon fixation. The IQ motif typically binds to calmodulin [28]; therefore, ANK-IQ members may act as calcium signal receptors, regulating calcium-dependent physiological processes within leaf cells. *SbANK94* and *SbANK95*, members of the ANK-RF subfamily, are highly expressed in stems, roots, and meristematic tissues, suggesting that they may regulate the development of vegetative organs through ubiquitination pathways.

Salt stress treatment revealed a fine division of labor within the *SbANK* gene family. The ANK-RF subfamily (*SbANK94*, *SbANK95*, *SbANK106*) specifically responded to moderate salt stress, while their expression declined under severe stress, exhibiting “early response” characteristics, suggesting they may be involved in the rapid transduction of osmotic regulation signals. The ANK-IQ subfamily (*SbANK68*, *SbANK69*) was upregulated only under severe salt stress, indicating its potential involvement in cellular defense mechanisms under severe stress, such as the scavenging of reactive oxygen species and the maintenance of protein homeostasis. In our expression analysis, the ANK-M subfamily exhibited the most extensive transcriptional response to salt stress, suggesting that it may be the subfamily within the *SbANK* gene family that plays a major role in regulating the response to salt stress: six members, including *SbANK58–63*, are consistently upregulated under both moderate and severe stress, while *SbANK136*, *SbANK137*, and *SbANK91* are induced only under severe stress. This gradient response pattern suggests the existence of a functional “relay” mechanism within the ANK-M subfamily: some members are responsible for basic stress adaptation, while others are recruited under extreme conditions. Analysis of cis-regulatory elements in promoters provides a molecular-level hypothesis for the aforementioned expression differences: the promoters of salt-stress-responsive genes are rich in jasmonic acid response elements and hypoxia-inducible elements, suggesting that they may mediate salt tolerance through the jasmonic acid signaling pathway or the hypoxia response pathway. The presence of ABA response elements is consistent with known salt stress signaling pathways. However, the mere presence of cis-acting elements does not prove the active involvement of the relevant signaling pathways. Further experimental validation can be conducted using methods such as promoter-reporter gene assays, transcription factor binding studies, or mutant analysis. Furthermore, none of the 10 candidate genes for salt stress response examined in this study were predicted to contain typical transmembrane domains or ion channel motifs; instead, they were classified into known protein–protein interaction functional groups (ANK-M as scaffold proteins, ANK-IQ as calmodulin-binding proteins, and ANK-RF as E3 ubiquitin ligases). Based on this, it is hypothesized that these genes do not function directly as ion channels or transporters, but instead synergistically regulate salt tolerance by modulating protein stability, ion homeostasis, or upstream pathways of stress-signal transduction. The expression of selected *ANK* genes in sorghum leaves under salt stress was validated by qRT-PCR. Although the transcriptomic dataset originated from a different genotype, qRT-PCR validation on our own material (Dali Shi) confirmed the same directional changes for the selected genes, with consistent upregulation patterns under salt stress. This concordance supports the reliability of the candidate gene identification despite the genotype difference. Ultimately, we found that *SbANK91*, *SbANK135*, and *SbANK136* are key candidate genes in the *SbANK* gene family that respond to salt stress. In the future, these candidate genes can be subjected to functional validation (e.g., through overexpression, CRISPR/Cas9 knockout, or heterologous expression) to determine their exact roles in salt tolerance. This study establishes a molecular basis for functional analysis of the *SbANK* gene family and provides putative gene resources for breeding salt-tolerant *S. bicolor*.

## 4. Materials and Methods

### 4.1. Cultivation and Processing of S. bicolor

The test material was the *S. bicolor* variety “Dali Shi,” purchased from Bailu International Grass Industry Co., Ltd. (Lanzhou, China). *S. bicolor* seeds were germinated in plastic Petri dishes (9 cm in diameter). After two weeks, seedlings of uniform size were selected and transplanted into plastic flower pots filled with a potting mix (vermiculite: potting mix = 1.5:1) (top dimensions: length × width = 10 cm × 10 cm; height 8.5 cm). Transplant 5 *S. bicolor* seedlings into each pot, then place them in a controlled environmental chamber (temperature 26 °C, humidity 50%, 10 h dark/14 h light) for cultivation. Water with deionized water within 48 h after transplanting, and then irrigate with Hoagland’s nutrient solution(Haibo Biotechnology Co., Ltd., Qingdao High-Tech Industrial Park, Qingdao, China). When the seedlings reach the three-leaf-and-one-heart stage, select seedlings with uniform growth and subject them to salt stress: First, treat the seedlings sequentially for 12 h with Hoagland’s solution containing 50 mmol/L and 100 mmol/L NaCl to reduce the stress response of sorghum when exposed to higher salt concentrations, followed by treatment with Hoagland’s solution containing 200 mmol/L NaCl for 24 h. Each treatment was repeated three times. After treatment, *S. bicolor* leaf tissue was collected, wrapped in aluminum foil, and flash-frozen in liquid nitrogen for 15 min, then stored at −80 °C for use in subsequent experiments.

### 4.2. Identification, Analysis of Physicochemical Properties, Subcellular Localization, and Prediction of Transmembrane Domains of Members of the S. bicolor ANK Gene Family

Obtaining genomic data and annotation files for *S. bicolor* from the Ensembl Plants database (Available online: https://plants.ensembl.org/index.html (accessed on 18 May 2026)). Download the Hidden Markov Model (.hmm) files for the ANK domain (PF00023 or SM00248) from the Pfam database (Available online: http://pfam.xfam.org (accessed on 18 May 2026)) [29], and use TBtools-II v2.390 to analyze the *S. bicolor* proteome to obtain candidate sequences containing the ANK domain. The Batch SMART tool (Available online: http://smart.embl-heidelberg.de/ (accessed on 18 May 2026)) was used to screen for ANK-domain-containing members, and domains unrelated to ANK were excluded. Submit the obtained candidate sequences to the NCBI CDD software (Available online: https://www.ncbi.nlm.nih.gov/Structure/bwrpsb/bwrpsb.cgi (accessed on 18 May 2026)) for domain validation [30,31], ultimately identifying members of the *SbANK* gene family. The domain composition of the SbANK protein was investigated utilizing the SMART database (Available online: http://smart.embl-heidelberg.de/ (accessed on 18 May 2026)), which provides individual domain maps for each protein sequence. Subsequently, the Pfam database was employed to refine the domain assignments, and based on the combined SMART/Pfam results, the 186 SbANK proteins were classified into 13 distinct subfamilies. Secondary structure prediction was performed using SOPMA (Available online: https://npsa-prabi.ibcp.fr/cgi-bin/npsa_autopmat.pl?page=npsa_sopma.html (accessed on 18 May 2026)) [32]. Physicochemical parameters—including molecular weight, theoretical isoelectric point (pI), and grand average of hydropathicity (GRAVY)—were computed via the ExPASy ProtParam server (Available online: https://web.expasy.org/protparam/ (accessed on 18 May 2026)) [33]. Subcellular localization predictions were performed using the PlantmPLoc (Available online: http://www.csbio.sjtu.edu.cn/bioinf/plant-multi/ (accessed on 18 May 2026)) [34], and transmembrane helix regions were recognized using TMHMM-2.0 (Available online: https://services.healthtech.dtu.dk/ (accessed on 18 May 2026)) [35].

### 4.3. Chromosomal Localization and Collinearity Analysis of the S. bicolor ANK Gene Family

Chromosomal information was extracted using TBtools-II v2.390 (Available online: https://github.com/CJ-Chen/TBtools/releases (accessed on 20 May 2026)), and the distribution of *SbANK* genes was plotted using the Gene Location Visualize from GTF/GFF tool [36]. A collinearity analysis was performed using the One Step McsanX tool in TBtools-II v2.390; the parameters were set as follows: CPU for BlastP = 2, e-value = 1 × 10^−3^, Number of Blast Hits = 10. A colinearity analysis was performed, yielding a file containing all tandem genes in the sorghum genome, and the results were visualized using the Multiple System Plot tool in TBtools-II v2.390 [37].

### 4.4. Phylogenetic Analysis of the ANK Gene Family in S. bicolor

For phylogenetic analysis, all 186 SbANK protein sequences were aligned using Clustal W (v1.83) in MEGA 12.0 software (Available online: https://www.megasoftware.net/ (accessed on 21 May 2026)) with default parameters (gap opening penalty = 10, gap extension penalty = 0.2, protein weight matrix = Gonnet series). Poorly aligned regions and ambiguous sites were manually inspected and trimmed using the alignment editor in MEGA. The optimal amino acid substitution model was selected using the ‘Find Best DNA/Protein Models (ML)’ tool in MEGA 12.0, which calculates BIC, AICc, and lnL values for 64 models. The model with the lowest BIC score (WAG + G + F) was chosen. The phylogenetic tree was constructed using the Maximum Likelihood (ML) method with the following parameters: Substitution Type = Amino Acid, Model/Method = WAG with Freqs. (+F) model, Rates among Sites = Gamma Distributed (G) with 5 discrete gamma categories, and Gaps/Missing Data Treatment = Partial-Deletion with a 95% site coverage cutoff. Branch support was assessed using the Bootstrap test with 1000 replicates, and format the resulting phylogenetic tree file using Adobe Illustrator 2021 software (Available online: https://www.adobe.com/products/illustrator/free-trial-download.html# (accessed on 21 May 2026)).

### 4.5. Analysis of Gene Structure and Protein Conservation Motifs in the S. bicolor ANK Family

To investigate the structural diversity of the SbANK proteins, conserved amino acid motifs were predicted using the MEME suite (Available online: https://meme-suite.org/meme/ (accessed on 21 May 2026)) with the following parameters: maximum number of motifs = 6, and motif width ranging from 6 to 100 amino acid residues. The resulting motif compositions were visualized with the “MEME Suite Wrapper” module of TBtools-II v2.390 [38]. In parallel, the exon–intron organization of each *SbANK* gene was determined by comparing the coding sequences with the corresponding genomic sequences extracted from the *S. bicolor* genome annotation file (GFF3), and the gene structure diagrams were generated using the Gene Structure View (Advanced) tool in TBtools-II v2.390.

### 4.6. Promoter Analysis of the S. bicolor ANK Gene Family

From the *S. bicolor* whole-genome sequence, a 2000 bp genomic sequence upstream of the translation start codon (ATG) of each *SbANK* gene was extracted as the promoter sequence. The promoter sequences were then submitted to the PlantCARE database (Available online: https://bioinformatics.psb.ugent.be/webtools/plantcare/html/ (accessed on 22 May 2026)) to identify cis-regulatory elements [39]. The identified elements were manually categorized into four functional classes (hormone-responsive, stress-responsive, growth/development-related, and light-response/metabolic elements), and their distribution patterns were summarized.

### 4.7. Expression Profile Analysis of the S. bicolor ANK Gene Family

The *S. bicolor* transcriptome dataset GSE140928 (containing FPKM values) under salt stress was downloaded from the NCBI GEO database. This includes RNA-Seq expression profiles of *S. bicolor* under normal conditions, moderate salt stress, and severe salt stress, with three biological replicates for each treatment condition. We extracted expression data (FPKM values) for members of the *SbANK* gene family under different salt stress conditions and plotted heatmaps using the Heatmap tool in TBtools-II v2.390. For the tissue-specific expression dataset by Davidson et al. [40], we similarly obtained the data from the published supplementary files and processed it using the same workflow.

Based on expression patterns observed in the transcriptomic data, 10 SbANK genes were selected for qRT-PCR validation; all selected genes showed significant upregulation under moderate (MS) or severe (SS) salt stress conditions (|log_2_FC| > 1.0, adjusted *p*-value < 0.05). For expression analysis under salt stress treatment, RNA was extracted from *S. bicolor* leaves using the ONOBATE Hard-to-Extract Plant RNA Extraction Kit (R4165-02) (ONOBATE Biotech Co., Ltd., Xianyang, China), and cDNA was synthesized via reverse transcription using the TransScript^®^ One-Step gDNA Removal and cDNA Synthesis SuperMix Kit (Beijing Quanshijin Biotechnology, Beijing, China). qPCR reaction mixture: 10 μL 2× BioGold SYBR qPCR Master Mix, 0.4 μL 50× ROX Reference Dye 1, 1 μL template DNA, 0.4 μL each of F and R primers (10 μmol/L), and RNase-free water to a total volume of 20 μL (The above reagents were purchased from.he above reagents were purchased from Beijing Quanshijin Biotechnology, Beijing, China). qPCR reaction program: 95 °C for 30 s; 95 °C for 5 s, 60 °C for 30 s, 40 cycles; dissociation. We determined the amplification curves and melting curve after the qRT-PCR reaction, and calculated gene expression levels using the 2^−ΔΔCt^ method [41]. The primers required for qRT-PCR are listed in Table S3, with the sorghum *PP2A* as the reference gene. For qRT-PCR validation, three biological replicates were performed, each consisting of independently treated plants grown in separate pots. For each biological replicate, three technical replicates were run for each gene and each treatment condition. The reference gene *PP2A* was used as an internal control to normalize gene expression levels. Preliminary data analysis was performed using Excel, while correlation analysis and the calculation of fitting parameters for the logistic equation were performed using SPSS 18.0 statistical software (Available online: https://spss.mairuan.com/ (accessed on 30 May 2026)). The figure was generated with GraphPad Prism 8.3 software (Available online: https://www.graphpad.com/updates/prism-830-release-notes (accessed on 30 May 2026)). We used Student’s *t*-test to indicate statistical significance. * *p* < 0.05, ** *p* < 0.01, *** *p* < 0.001, and **** *p* < 0.0001.

## 5. Conclusions

In this research, we performed genome-wide identification and characterization of the *ANK* gene family in *Sorghum bicolor*, identifying 186 *SbANK* family members and classifying them into 13 subfamilies. Bioinformatics analysis revealed their basic genetic characteristics, including protein physicochemical properties, chromosomal localization, conserved sequences and structures, and cis-acting elements in the SbANK promoters. Transcriptomic analysis and qRT-PCR validation indicated that *SbANK91*, *SbANK135*, and *SbANK136* were significantly upregulated under salt stress conditions, making them putative candidate genes for the salt stress response. Overall, this research establishes a foundational resource for the functional characterization of the *SbANK* gene family and lays the groundwork for future functional characterization and applications in stress tolerance.

## Data Availability

The original contributions presented in the study are included in the article; further inquiries can be directed to the corresponding authors.

## Supplementary Materials

The following supporting information can be downloaded at https://www.mdpi.com/article/10.3390/ijms27177810/s1.
