# Supplementary material for: Genome-Wide Identification and Expression Pattern of the ANK Gene Family in Sorghum bicolor Under Salt Stress
Source: Int J Mol Sci. 2026 Aug 31;27(17):7810. doi: 10.3390/ijms27177810 (PMC13566331; doi:10.3390/ijms27177810)
Supplement: Supplementary file 1 [file ijms-27-07810-s001.zip › ijms-4411035-supplementary.pdf]

Table S1. Analysis of the Physical and Chemical Properties of Members of the SbANK Gene Family

Sequence ID	Gene Name	Number of Amino Acid	Molecular Weight	Theoretical pI	Instability Index	Aliphatic Index	Grand Average of Hydropathicity	Subcellular localization prediction	Category
transcript:OQU78664	SbANK1	477	52377.19	6.23	31.15	93.1	-0.104	chloroplast	ANK,BPA
transcript:OQU78665	SbANK2	427	46878.83	6.66	28.58	88.95	-0.131	chloroplast	ANK，BPA
transcript:OQU79515	SbANK3	314	35288.95	6.76	39	97.93	-0.118	cytosol	ANK，BPA
transcript:EES16297	SbANK4	390	41318.26	4.59	40.12	91.62	-0.288	chloroplast	ANK,BTB
transcript:EES16520	SbANK5	480	50496.81	6.11	42.93	92.94	0.028	endoplasmic reticulum	ANK,BTB
transcript:EES17326	SbANK6	366	40019.03	6.57	50.62	78.2	-0.564	cytosol	ANK,BTB
transcript:OQU79516	SbANK7	376	41350.61	6.8	54.87	74.31	-0.535	mitochondrion	ANK，BTB
transcript:KXG23773	SbANK8	1102	122580.38	7.61	47.36	82.89	-0.328	nucleus	ANK，BTB
transcript:KXG23772	SbANK9	1100	122535.39	7.61	47.24	83.05	-0.327	nucleus	ANK，BTB
transcript:OQU79519	SbANK10	394	43021.17	5.34	34.26	93.91	-0.146	cytosol	ANK，BTB
transcript:KXG22907	SbANK11	434	47601.88	6.54	28.72	94.47	-0.064	cytosol	ANK，BTB
transcri	SbANK	444	47493.3	5.97	30.98	89.55	-0.095	plasma	ANK，

pt:EES1 7332	12		3					membr ane	GPCR
transcri pt:OQ U79686	SbANK 13	550	61163.7 4	6.53	38.11	99	0.095	plasma membr ane	ANK，IQ
transcri pt:KXG 23895	SbANK 14	426	46797.8 9	5.51	36.01	90.33	-0.147	cytosol	ANK，IQ
transcri pt:OQ U79518	SbANK 15	418	45098.8 9	5.56	28.64	100.5	0.033	cytosol	ANK，IQ
transcri pt:KXG 23574	SbANK 16	588	62084.8 2	5.94	54.89	74.66	-0.278	chlorop last	ANK，IQ
transcri pt:OQ U79517	SbANK 17	398	43741.3 1	6.77	31.04	86.63	-0.169	cytosol	ANK，IQ
transcri pt:EES1 6309	SbANK 18	417	44986.6 9	5.46	34.57	93	-0.044	chlorop last	ANK，IQ
transcri pt:EES1 6450	SbANK 19	437	48461.6 4	5.41	33.66	92.7	-0.155	cytosol	ANK，IQ
transcri pt:OQ U93303	SbANK 20	381	41309.9 3	4.67	35.43	81.34	-0.459	mitoch ondrio n	ANK，IQ
transcri pt:KXG 37850	SbANK 21	405	44172.0 1	7.5	28.98	99.28	-0.156	cytosol	ANK，IQ
transcri pt:OQ U91202	SbANK 22	430	46807.0 7	8.29	33.98	98.05	-0.145	cytosol	ANK，IQ
transcri pt:OQ U93285	SbANK 23	435	48945.8 1	6.64	53.61	88.37	-0.499	chlorop last	ANK，IQ
transcri pt:OQ U91909	SbANK 24	1022	113534. 05	5.63	49.33	74.56	-0.512	nucleus	ANK,I T
transcri pt:OQ U91908	SbANK 25	1021	113462. 97	5.63	49.45	74.53	-0.514	nucleus	ANK,I T
transcri pt:KXG 37960	SbANK 26	414	45451.8 3	6.83	35.7	87.49	-0.353	cytosol	ANK，IT

transcript:EER91188	SbANK27	447	49288.21	6.05	33.87	87.16	-0.354	cytosol	ANK，IT
transcript:KXG39235	SbANK28	988	110593.37	6.2	46.08	78.65	-0.485	peroxisome	ANK，IT
transcript:EER94762	SbANK29	1024	114399.66	5.86	45.84	78.76	-0.456	nucleus	ANK，IT
transcript:OQU92798	SbANK30	680	73318.22	9.81	48.65	97.1	-0.128	plasma membrane	ANK，IT
transcript:EER95067	SbANK31	688	74036.01	9.8	48.29	96.26	-0.132	plasma membrane	ANK，IT
transcript:EER92859	SbANK32	208	21992.35	4.38	36.15	85.05	-0.274	cytosol	ANK，IT
transcript:KXG39650	SbANK33	567	61727.98	9.48	44.26	95.71	-0.142	plasma membrane	ANK，IT
transcript:KXG37852	SbANK34	450	48760.17	7.14	19.9	91.56	-0.173	cytosol	ANK, M
transcript:EER93669	SbANK35	356	38467.35	6.83	19.16	94.89	-0.146	cytosol	ANK, M
transcript:EER92045	SbANK36	520	55549.13	6.02	48.91	81.85	-0.044	nucleus	ANK, M
transcript:EER93086	SbANK37	351	37267.59	4.6	43.24	71.14	-0.495	mitochondrion	ANK, M
transcript:OQU91203	SbANK38	411	44607.25	6.18	37.29	77.03	-0.479	chloroplast	ANK, M
transcript:EER91080	SbANK39	490	53003.08	6.13	35.89	83.14	-0.361	chloroplast	ANK, M
transcript:EER93617	SbANK40	745	79472.39	6.51	59.83	67.15	-0.371	nucleus	ANK, M
transcript:KXG	SbANK41	141	15286.7	4.3	34.69	74.18	-0.608	nucleus	ANK, M

37573									
transcript:KXG37848	SbANK42	497	54390.29	6.08	25.41	84.45	-0.228	cytosol	ANK, M
transcript:OQU93088	SbANK43	994	110359.29	5.71	47.07	72.33	-0.549	nucleus	ANK, M
transcript:EER91108	SbANK44	598	66536.8	5.95	55.28	90.9	-0.3	chloroplast	ANK, M
transcript:EER93420	SbANK45	244	25301.41	5.09	33.46	92.21	-0.108	cytosol	ANK, M
transcript:EER92481	SbANK46	448	48063.5	8.42	51.59	79.84	-0.229	nucleus	ANK, M
transcript:KXG38850	SbANK47	436	47310.55	6.32	32.23	87.82	-0.169	cytosol	ANK, M
transcript:EER93157	SbANK48	969	105842.97	5.29	49.84	79.29	-0.301	plasma membrane	ANK, M
transcript:EER90917	SbANK49	556	61761.41	8.67	38.88	98.78	-0.078	plasma membrane	ANK, M
transcript:EER93727	SbANK50	557	57219.77	9.03	27.99	88.29	-0.065	cytosol	ANK, M
transcript:EER90916	SbANK51	565	62774.42	9.08	36.03	93.75	-0.149	plasma membrane	ANK, M
transcript:KXG37496	SbANK52	336	36877.59	4.75	42.74	80.54	-0.221	nucleus	ANK, M
transcript:EER91850	SbANK53	254	26917.89	6.61	38.7	64.25	-0.766	nucleus	ANK, M
transcript:EER98028	SbANK54	1071	116167.5	9.15	42.7	76.52	-0.466	nucleus	ANK, M
transcript:OQU89729	SbANK55	566	64608.89	9.18	51.4	94.26	-0.234	cytosol	ANK, M
transcri	SbANK	415	47196.4	8.91	53.55	91.42	-0.276	cytosol	ANK,

pt:OQU89728	56		7						M
transcript:EER97033	SbANK57	323	34787.79	4.41	47.89	74.74	-0.532	mitochondrion	ANK, M
transcript:KXG35329	SbANK58	441	47361.39	6.09	41.68	95.94	-0.123	chloroplast	ANK, M
transcript:OQU89200	SbANK59	442	47489.52	6.09	42.04	95.72	-0.131	chloroplast	ANK, M
transcript:OQU89196	SbANK60	425	45413.33	6.75	43.16	96.8	-0.054	chloroplast	ANK, M
transcript:OQU89201	SbANK61	437	46952.04	6.52	41.6	96.82	-0.105	chloroplast	ANK, M
transcript:OQU89199	SbANK62	426	45541.46	6.75	43.54	96.57	-0.062	chloroplast	ANK, M
transcript:OQU89198	SbANK63	438	47080.17	6.52	41.96	96.6	-0.113	chloroplast	ANK, M
transcript:EER97545	SbANK64	291	31435.5	7.87	55.94	61.72	-0.504	nucleus	ANK, M
transcript:KXG36244	SbANK65	914	102439.49	8.36	40.21	77.23	-0.513	nucleus	ANK, M
transcript:EER98424	SbANK66	651	72992.65	8.31	65.74	73.29	-0.608	nucleus	ANK, M
transcript:KXG36732	SbANK67	1017	113665.29	5.55	47.86	74.84	-0.548	nucleus	ANK, M
transcript:KXG36731	SbANK68	1028	114724.47	5.55	47.41	75.18	-0.539	nucleus	ANK, M
transcript:KXG36730	SbANK69	1030	114866.63	5.55	47.34	75.22	-0.535	nucleus	ANK, M
transcript:OQU89270	SbANK70	562	61346.17	7.36	32.62	90.66	-0.049	plasma membrane	ANK, M

transcript:EER98436	SbANK71	516	55380.95	6.23	55.19	76.05	-0.299	nucleus	ANK, M
transcript:EER98651	SbANK72	443	47301.94	5.64	29.38	93.95	-0.095	chloroplast	ANK, M
transcript:EER97319	SbANK73	660	70752.07	9.01	45.71	98.83	0.23	nucleus	ANK, M
transcript:EER99460	SbANK74	508	53604.41	6.97	43.77	95	0.057	nucleus	ANK, M
transcript:EER97054	SbANK75	562	61625.35	8.85	38.71	99.82	-0.031	plasma membrane	ANK, M
transcript:EER95927	SbANK76	919	100649.17	8.18	43.78	86.54	-0.141	chloroplast	ANK, M
transcript:EER99172	SbANK77	836	93765.41	6.89	51.12	75.61	-0.531	nucleus	ANK, M
transcript:KXG36380	SbANK78	396	42258.4	6.53	35.55	93.74	0.101	vacular membrane	ANK, M
transcript:KXG36379	SbANK79	399	42633.83	6.39	35.31	93.76	0.109	vacular membrane	ANK, M
transcript:KXG36384	SbANK80	399	42122.27	5.86	31.67	102.18	0.157	cytosol	ANK, M
transcript:EER96593	SbANK81	484	52047.7	9.31	39.02	108.51	0.406	plasma membrane	ANK, M
transcript:EER99440	SbANK82	615	66603.48	9.51	60.2	79.11	-0.34	chloroplast	ANK, M
transcript:KXG35317	SbANK83	434	47076.71	6.06	40.43	93.18	-0.132	mitochondrion	ANK, M
transcript:EER97396	SbANK84	680	72441.2	6.88	59.24	73.66	-0.392	nucleus	ANK, M
transcript:EER	SbANK85	184	20050.89	8.92	44.56	80.65	-0.29	chloroplast	ANK, M

97310									
transcript:EER99756	SbANK86	442	49379.21	6.6	37.05	87.9	-0.383	cytosol	ANK, M
transcript:EER99842	SbANK87	155	15965.81	4.41	23.62	94.58	0.047	cytosol	ANK, M
transcript:KXG22064	SbANK88	842	93389.95	6.31	35.84	97.49	-0.031	plasma membrane	ANK, M
transcript:OQU78061	SbANK89	734	81880.87	6.49	34.38	96.99	-0.087	cytosol	ANK, M
transcript:OQU78060	SbANK90	575	63292.1	5.55	37.11	98.7	-0.157	cytosol	ANK, M
transcript:KXG21535	SbANK91	525	58565.39	8.78	37.91	91.96	-0.249	cytosol	ANK, M
transcript:OQU77325	SbANK92	611	64097.55	8.54	53.65	68.94	-0.244	chloroplast	ANK, M
transcript:EES18294	SbANK93	699	74824.93	8.42	36.93	94.58	0.108	plasma membrane	ANK, M
transcript:EES18892	SbANK94	465	49424.12	6.64	48.8	83.89	-0.145	nucleus	ANK, M
transcript:OQU77236	SbANK95	429	45587.77	6.37	50.58	83.4	-0.136	nucleus	ANK, M
transcript:OQU78066	SbANK96	688	76995.25	5.82	31.56	97.49	-0.067	cytosol	ANK, M
transcript:EES19575	SbANK97	837	93301.97	6.06	32.3	96.43	-0.029	plasma membrane	ANK, M
transcript:KXG22060	SbANK98	695	77124.45	6.49	31.78	96.69	-0.099	endoplasmic reticulum	ANK, M
transcript:KXG21921	SbANK99	1627	177976.28	5.91	42.09	87.91	-0.197	nucleus	ANK, M

transcript:KXG21092	SbANK100	627	68485.46	6.91	38.17	99.92	0.243	plasma membrane	ANK, M
transcript:OQU78058	SbANK101	626	68459.08	6.31	42.16	103.63	0.21	plasma membrane	ANK, M
transcript:EES18832	SbANK102	460	48798.67	5.09	32.56	88.41	-0.298	chloroplast	ANK, M
transcript:EES00131	SbANK103	583	64460.56	5.66	43.48	96.74	-0.225	cytosol	ANK, M
transcript:EES01985	SbANK104	845	95220.08	6.97	39.15	81.96	-0.477	nucleus	ANK, M
transcript:EES03608	SbANK105	473	53269.5	6.69	47.14	77.76	-0.59	peroxisome	ANK, M
transcript:EES02150	SbANK106	484	51264.12	6.77	45.43	87.5	-0.104	nucleus	ANK, M
transcript:EES03354	SbANK107	885	99321.75	7.04	39.84	94.42	-0.13	chloroplast	ANK, M
transcript:EES03825	SbANK108	406	42655.64	5.71	41.93	97.51	0.054	sytosol	ANK, M
transcript:EES01524	SbANK109	621	67648.06	5.64	50.5	86.76	-0.277	chloroplast	ANK, M
transcript:OQU88196	SbANK110	507	53644.31	6.01	43.54	92.07	0.03	chloroplast	ANK, M
transcript:EES03848	SbANK111	556	60504.46	9.25	39.05	97.37	-0.12	plasma membrane	ANK, M
transcript:KXG33869	SbANK112	478	53264.14	9.17	40.14	81.59	-0.364	chloroplast	ANK, M
transcript:EES06098	SbANK113	165	18376.85	7.05	54.73	71.03	-0.644	mitochondrion	ANK, M
transcript:EES0	SbANK114	497	53846.74	6.93	51.52	85.23	0.029	nucleus	ANK, M

5937									
transcript:OQU85359	SbANK115	839	94010.72	6.45	47.75	83.16	-0.398	chloroplast	ANK, M
transcript:EES06629	SbANK116	485	53939.16	6.45	46.6	85.48	-0.315	cytosol	ANK, M
transcript:EES05988	SbANK117	631	70053.12	6.8	49.53	72.88	-0.644	cytosol	ANK, M
transcript:OQU84955	SbANK118	481	51832.41	6.43	32.31	93.18	-0.173	chloroplast	ANK, M
transcript:OQU84957	SbANK119	357	38966.66	5.24	26.08	89.92	-0.177	cytosol	ANK, M
transcript:EES04435	SbANK120	299	32888.65	8.61	49.56	91.4	-0.399	chloroplast	ANK, M
transcript:EES06392	SbANK121	617	68149.19	6.26	32.48	85.56	-0.205	plasma membrane	ANK, M
transcript:KXG29635	SbANK122	665	73549.35	6.24	33.94	85.25	-0.177	plasma membrane	ANK, M
transcript:OQU84959	SbANK123	403	43943.39	5.35	37.98	90.15	-0.182	chloroplast	ANK, M
transcript:KXG30235	SbANK124	396	42926.15	5.4	34.42	91.24	-0.118	chloroplast	ANK, M
transcript:KXG30372	SbANK125	453	51407.23	9.07	51.38	92.65	-0.263	cytosol	ANK, M
transcript:EES07299	SbANK126	529	57623.34	8.42	29.81	103.16	0.049	plasma membrane	ANK，M
transcript:KXG30444	SbANK127	589	64310.32	5.38	40.89	95.09	0.033	mitochondrion	ANK，M
transcript:EES05379	SbANK128	709	76884.24	8.62	45.4	82.79	-0.297	nucleus	ANK，M
transcri	SbANK	639	70243.5	6.74	46.78	90.3	-0.289	cytosol	ANK，

pt:EES06857	129								M
transcript:KXG30604	SbANK130	489	54495.38	9.1	41.39	79.84	-0.478	chloroplast	ANK，M
transcript:OQU84849	SbANK131	637	69400.37	8.37	40.64	99.48	0.173	plasma membrane	ANK，M
transcript:KXG28826	SbANK132	405	44941.05	8.99	41.92	101.38	0.057	plasma membrane	ANK，M
transcript:OQU83146	SbANK133	114	12281.26	6.15	43.57	84.04	0.129	cytosol	ANK，M
transcript:OQU84108	SbANK134	761	83295.17	7.92	38.65	88.76	-0.14	plasma membrane	ANK，MS
transcript:OQU84107	SbANK135	669	72976.4	6.36	37.57	79.22	-0.362	endoplasmic reticulum	ANK，MS
transcript:KXG28660	SbANK136	551	61414.59	7.64	35.98	79.36	-0.223	plasma membrane	ANK，MS
transcript:OQU83632	SbANK137	430	47589.7	7.04	36.79	77.65	-0.295	endoplasmic reticulum	ANK，O
transcript:OQU83028	SbANK138	673	71403.17	9.14	38.08	94.71	0.18	plasma membrane	ANK，O
transcript:OQU83029	SbANK139	676	71646.43	9.14	37.96	94.72	0.184	plasma membrane	ANK，O
transcript:EES10220	SbANK140	417	45900.98	10.05	45.03	90.55	-0.156	mitochondrion	ANK,RF
transcript:EES08937	SbANK141	440	48253.1	10.01	42.41	84.7	-0.289	chloroplast	ANK,RF
transcript:OQU83151	SbANK142	146	16183.89	8.31	59.7	100.21	0.079	cytosol	ANK，RF
transcri	SbANK	90	9850.41	6.14	44.16	92.22	0.19	chlorop	ANK，

pt:KXG28088	143							last	RF
transcript:OQU83144	SbANK144	402	43536.01	6.43	28.17	95.42	0.06	endoplasmic reticulum	ANK，RF
transcript:KXG27928	SbANK145	664	70733.06	8.27	35.26	99.53	0.132	plasma membrane	ANK，RF
transcript:KXG27920	SbANK146	493	52807.94	9.11	34.13	100.16	0.199	plasma membrane	ANK，RF
transcript:OQU83139	SbANK147	224	24180	6.45	48.6	112.41	0.019	chloroplast	ANK，RF
transcript:KXG28091	SbANK148	594	65225.78	8.76	28.42	101.09	0.063	plasma membrane	ANK，RF
transcript:EES09234	SbANK149	484	50585.82	6.1	43.69	93.18	0.048	endoplasmic reticulum	ANK，RF
transcript:OQU83143	SbANK150	349	37627.81	6.42	38.15	90.6	-0.125	cytosol	ANK，RF
transcript:KXG28374	SbANK151	654	70732.2	9.13	41.25	100.6	0.097	plasma membrane	ANK，RF
transcript:EES09724	SbANK152	650	70319.67	9.13	41.31	99.57	0.079	plasma membrane	ANK，RF
transcript:KXG28093	SbANK153	934	101886.03	7.19	36.3	100.95	0.074	plasma membrane	ANK，RF
transcript:EES09541	SbANK154	624	68401.37	5.02	41.02	89.49	-0.182	nucleus	ANK，SBP
transcript:EES12357	SbANK155	671	70830.86	9.15	36.72	88.36	-0.029	chloroplast	ANK，TM
transcript:OQU81848	SbANK156	594	63028.95	9.3	38.26	88.77	-0.071	chloroplast	ANK，TM
transcri	SbANK	769	81019.2	8.62	37.56	90.98	-0.031	chlorop	ANK，

pt:EES11039	157		2					last	TM
transcript:EES13117	SbANK158	334	35670.49	4.54	45.25	75	-0.354	endoplasmic reticulum	ANK，TM
transcript:KXG26960	SbANK159	668	74137.42	7.75	51.3	66.29	-0.733	nucleus	ANK，TM
transcript:KXG26959	SbANK160	507	56486.11	8.99	45.42	67.85	-0.705	chloroplast	ANK，TM
transcript:EES10868	SbANK161	725	82245.13	8.51	32.92	103.24	0.039	plasma membrane	ANK，TM
transcript:OQU81398	SbANK162	995	111783.6	5.95	48.11	74.03	-0.586	nucleus	ANK，TPR
transcript:OQU80692	SbANK163	1084	119570.82	8.78	36.74	91.99	-0.21	endoplasmic reticulum	ANK，TPR
transcript:OQU80690	SbANK164	1116	123078.96	8.73	37.27	91.81	-0.18	endoplasmic reticulum	ANK，TPR
transcript:OQU80688	SbANK165	1078	118938.13	8.8	35.82	92.14	-0.209	endoplasmic reticulum	ANK，TPR
transcript:OQU80686	SbANK166	1079	119011.14	8.75	35.73	91.7	-0.216	endoplasmic reticulum	ANK，TPR
transcript:OQU80689	SbANK167	1058	117182.19	8.7	35.66	92.95	-0.213	endoplasmic reticulum	ANK，TPR
transcript:OQU80691	SbANK168	1064	117814.88	8.69	36.6	92.79	-0.214	endoplasmic reticulum	ANK，TPR
transcript:OQ	SbANK169	1059	117255.2	8.65	35.57	92.49	-0.22	endoplasmic	ANK，TPR

U80687								reticulum	
transcript:EES14803	SbANK170	483	51907.96	8.95	46.4	91.95	0.063	nucleus	ANK，TPR
transcript:EES15272	SbANK171	335	36260.3	4.48	47.49	61.28	-0.701	nucleus	ANK，TPR
transcript:KXG24315	SbANK172	617	67500.19	8.83	33.71	90.31	-0.092	plasma membrane	ANK，TPR
transcript:EES15259	SbANK173	562	61569.45	9.56	41.32	102.94	-0.022	plasma membrane	ANK，TPR
transcript:OQU80298	SbANK174	695	75305.26	8.79	43.13	90.73	-0.033	plasma membrane	ANK，TPR
transcript:OQU80812	SbANK175	824	92894.43	6.58	49.52	76.58	-0.554	nucleus	ANK，TPR
transcript:KXG19893	SbANK176	580	65485.29	4.74	50.7	88.43	-0.307	cytosol	ANK，TPR
transcript:KXG19892	SbANK177	545	61376.31	4.72	50.53	84.99	-0.373	mitochondrion	ANK，TPR
transcript:OQU75733	SbANK178	352	36877.25	6.52	45.74	93.49	0.118	nucleus	ANK，TPR
transcript:OQU75732	SbANK179	324	33787.73	6.65	46.96	91.91	0.11	nucleus	ANK，TPR
transcript:KXG19181	SbANK180	406	42103.15	6.59	42.77	93.87	0.103	nucleus	ANK，TPR
transcript:KXG19708	SbANK181	854	97265.64	5.95	42.89	94.56	-0.15	plasma membrane	ANK，TPR
transcript:EER88713	SbANK182	640	70149.4	6.41	29.56	80.89	-0.209	plasma membrane	ANK,ZF
transcript:EER88274	SbANK183	758	81385.34	6.6	40.42	94.16	-0.001	plasma membrane	ANK,ZF

transcript:EER87700	SbANK184	806	86095.93	5.33	40.63	94.8	0.053	plasma membrane	ANK,ZF
transcript:EER87702	SbANK185	736	79989.25	6.69	42.23	92.88	-0.105	plasma membrane	ANK,ZF
transcript:EER89595	SbANK186	378	42061.42	6.34	35.08	91.61	-0.158	chloroplast	ANK,ZF

Table S2. CDS sequences of the *SbANK* gene family

>SbANK1
MRRGSEMNRRPAYIGTCKGNDRCNAAADGNLRVFKRIASALDGGKGRIREAVEAVRDELGDGV
LHVAAALGRTRVCAYLVEELHMDINAAAVSGQTPLSYAVCSDDNVEVVRYLLDHDADPDKPVY
HGCNSLHIAVAKGLCVMVELLLSKGADVNSMSYCGTPLNVAAMENQGAVMKILLDYNADCN
KEYSTIFTPLSTALCARSLSCVKLLIKAGADVTTKGTHKFSPLIFAANEGLTDFYECLLEAGADPNV
PDDEKFWACDQPVSWILWRGVNTRSLKLSIRCLLLKSGLLPIEIAALENRRKDVEILLRVTARIPSV
PDWSVDGVLAYVKSIPTDEDDPVYKMNLADLKLEGNKAFKKKDYYTAVKFYSVAIMKNANSVD
ATLFSNRSLCWIKMGEGNKALIDAEACRLFQPYWAKACYRQGAAHMFLKDYEKACGAFLDGL
KLDPANAELENELRESLNSLTISRGTTKVSE
>SbANK2
MRRGSEMNRRPAYIGTCKGNDRCNAAADGNLRVFKRIASALDGGKGRIREAVEAVRDELGDGV
LHVAAALGRTRVCAYLVEELHMDINAAAVSGQTPLSYAVCSDDNVEVVRYLLDHDADPDKPVY
HGCNSLHIAVAKGLCVMVELLLSKGADVNSMSYCGTPLNVAAMENQGAVMKILLDYNADCN
KEYSTIFTPLSTALCARSLSCVKLLIKAGADVTTKGTHKFSPLIFAANEGLTDFYECLLEAGADPNV
PDDEKFWACDQPVSWILWRGVNTRSLKLSIRCLLLKDDPVYKMNLADLKLEGNKAFKKKDYYT
AVKFYSVAIMKNANSVDATLFSNRSLCWIKMGEGNKALIDAEACRLFQPYWAKACYRQGAAH
MFLKDYEKACGAFLDGLKLDPANAELENELRESLNSLTISRGTTKVSE
>SbANK3
MAPSTADLAIQVTSDGNLCLLNQISKELELRGVKGFKERSLLHFAAGSGQLEACKFLAEGDGNLP
VLRYLLDRGGDPAMPDARGLTLLHNAAGNVVHDDLSPLTMACCAHSLKCLKLLTQAGADVNS
KSPHDAPILMLVVNEDLIDIFKLLLEVGAEPNIPDRRGKFPIMLAAGREHRDLVEILFPQTKPISLFP
DWSVDGIIRAMKYLSFGPQEQNFADYYILIYMLEPLERYGLVTEHASSMFRSRWKNRLLLRSNDK
THMMPTIFANRSLCWLRLREGERALSDAQRCKTLDPHWAKAWYREDMALSFLKV
>SbANK4
MAPTRSTSDARSVWSSSLRPPLDLQPAVLPEDDDGDDDRAVAELLNAALEGDLSRVKELVEELN
EEGKGVDEVVTAAAVGVTGTKRRGPLHLAAANGNAEMCRFLIKVGDVDVDAADSDGATPLIFA
IQGLGSTTVVRLLLNNGANLNKADNSGVAPLHIAAERGLYEVAELLLSREAEVDLMCENGGAPI
HIAAENGHAKFLKLLLQHEADYNAGDPVTALVIAAGKGLTDCIKCLLKAGADANIPDEDGKLP
VQIAACQGWKECVEILFPVTTPLAEYATWSIDGIIQHEKTASSEPQLQVSNSDDKENAVSDDDTN
KDTQPHQSKSCSEKGKALTTVEENGGGIETLMSGLNLGPEDPSDKASMPLLRRSTRVRQPNVRIS
GPEWAR

>SbANK5
MSSEDSLKSLSLDYLNLLINGQAFSDVAFSVEGRLVHAHRCVLAARSLFFRKLFCGLDPNHPPPPP
PPPQPPTGGGGGARTPDLVIPVSSIRYEVLVLVLQFLYSGQASVAAPKSGPLPGCGARGCWHTRC
GAAVDLALDTLAAARSFGVEQLALLVQKQLESMVKEASVDDVMKVLMASRKFEMQELWATCS
HLVARSGLSADLLAKHLPIDVVAKIEEIRAKSPAGAAVSSATTPRSPFLTHHYLPINAASSAADRD
HKIRRMRRALDAADIELVKLMVMGEGLDLDDALAVHYAVQHCGRDVVKALLELGAADVNSR
AGPTGKTALHLAAEMVSPDMVSVLLDHHADPSARTLDGVTPLDVLRGLTSEFLFKGAVPGLTHI
EPNKLRLCLELVQSAVMVTTRDEGAGDAGGSDGGGNFPRSDADDSLVSLTMNSTLMYQGQEM
AAAAVAAGEARKGNGGGGGRGSPSNLYFPNGGFP
>SbANK6
MRGEGEGRRRGGGPGRPDGMRGEGEERRRGSGRRRAGRSRGEQTIIRGQPGTTIALDLEHADPN
KADDSGITPLHIAATQGYYEIAEYLLSKGANVDPLCQDGESPLLCAALQGNERIVTLLLKHNADY
NRLSKKMLTPLLASLHGSSLNCLEILIEAGADVNGAGCSVPPIALAAYKGLHGCIKCLLRNGANP
NVPDEDNNIPIQIAAVKGWERCVEILLPHAPHLAQYEGLTTSEIVQWEKTDRFVRSSRAIEQGDA
AYRRKEYDIALRFYTEAAQVGHQDPTLYAKRSLCHLYLRVPAKYTEEAINYMNMVQPNLSTLDP
ESDAKRMVLDFGRTWKESGFASTPGSGSSPTDKTDKTSSGEHQ
>SbANK7
MSGEGEGRRRGSGPGRPDGMRGEGEERRRGSGRRRAGRSRGEQPNRAEGMTPLMFASHHVGNE
AIVKYLISNQADPNKADNSGITPLHIAARQGCYEIAEYLLSKGANVDPLCQDGQSPLLCAALQGN
ERIVTLLLKHNADYNRMSKKMITPLLASLHCSSLSCLEILIEAGADVNGAGCSVPPIALAAYKGLH
GCIQCLLRNGANPNVPDEDNNIPIQFAAFKGWERCVEILLPYAPHLAQYEGLTTSEIVQWEKTDR
FVRSSRAIEQGDAAYRRKEYDIALRFYTEAAQVGHQDPTLYAKRSLCHLYLRVPAKYTEEAINYM
NMVQPNLSTLDPESDAKRMVLDFGRTWKESGFASTPGSGSSPTDKTTSEEHQ
>SbANK8
MGENSSEETSGAGTRGSMERVKHDNFCRAVAEDNTALLLSAVGNFRKEAMCRIRKGSDASRVL
DKEMSTRLLHLACKHDAVECARLLLEGGSGIMAAPIDARDQLTRTPLHVAAETHSARCIQLLLSK
NARTDLRVVDGRPLLPLEIALMSRRVQTNWSVENPIEDLLSFLKQRDLDAVRLLAEKTRVVGELA
YRYAMEGSIPALAMLLLVVEEKISSHVSVVIEGVRTKRSIYNSIVDEALSMGDASARDGNERKKAL
LCEIQLLNQFGSASWRDHNDRRTLPPLLRAAKVGDMDVIKMLLMGNVDVNEADSEGNTALHW
CLSGASCTKEPRIVWLLLKNGARVFQGNKLGLTPVHSAAAKGNYKALQSLLLHAQDCVDIPTKT
KETPLFFAVKNGSVDCVKLLLRFGADTKARNLRKQRPIDVATSQDMRFILSSANVLPSSGNHSSPQ
KNRVMRKEIYKELLGDEFDDLDKDYCDESYTGLKTSVGQRDFRSSNRSAQGPKSKSQYVPKQAQ
GSKFVPRNNHWPKHDYTRKIFVGGLPPSVDSDYLIEFFNAEFGPVEEAVVIGIPIGNRVQSRGFGF
VKFEREEDMISAKEAHHVYMLGKRVEVKDAVARAYLPLEDQRYTSLRECIKERPKVHRVLDGEL
TEEHNLQKRQPLPEKCLPSWFFIFRKWLPGFLEDETARLGGERYPLSSLKGDFRATCRMELDHTAL
GYPKLSDFMRSLPGICRMCVVPVGSGPATHMVLLPPLSRPKYVPLLEPCSFDRDELAESVSDHHSP
RSPLNANIADDSPHNTDSQQGDDACSETNVQILQVDDACSETCVQSQHGHESSRSSAESQQDGS
SSDNGSLLDDVPVSTTKLDLIESVTSVKPALIESVPRRNPDLTESVPPRKPDLVESVPARTNLMEYGF
LPQKNESGPILKPNLLQFGPSRKLELIESKPSECFIDRPFERPAVTPSSCESDMRFSFFQSQWDKYLTP
YPRSDCCIICRSCEAGMQLVPCLHKICVACMMRCNIRACMVCGTSVSGVKSSPAVDATYRYMGL
TERALDQRCQLMVVCRGAEAIVRCSPCMHTIACRGCFLASVTLLKSCTTCGFMVEHFKIV
>SbANK9
MGENSSEETSGAGTRGSMERVKHDNFCRAVAEDNTALLLSAVGNFRKEAMCRIRKGSDASRVL
DKEMSTRLLHLACKHDAVECARLLLEGGSGIMAAPIDARDQLTRTPLHVAAETHSARCIQLLLSK

NARTDLRVVDGRPLLPLEIALMSRRVQTNWSVENPIEDLLSFLKQRDLDAVRLLAEKTRVVGELA
YRYAMEGSIPALAMLLLVVEEKISSHVSVVIEGVRTKRSIYNSIVDEALSMGDASARDGNERKKAL
LCEIQLLNQFGSASWRDHNDRRTLPPLLRAAKVGDMDVIKMLLMGNVDVNEADSEGNTALHW
CLSGASCTKEPRIVWLLLKNGARVFQGNKLGLTPVHSAAAKGNYKALQSLLLHAQDCVDIPTKT
KETPLFFAVKNGSVDCVKLLLRFGADTKARNLRKQRPIDVATSQDMRFILSSANVLPWNHSSPQK
NRVMRKEIYKELLGDEFDDLDKDYCDESYTGLKTSVGQRDFRSSNRSAQGPKSKSQYVPKQAQGS
KFVPRNNHWPKHDYTRKIFVGGLPPSVDSDYLIEFFNAEFGPVEEAVVIGIPIGNRVQSRGFGFVKF
EREEDMISAKEAHHVYMLGKRVEVKDAVARAYLPLEDQRYTSLRECIKERPKVHRVLDGELTEE
HNLQKRQPLPEKCLPSWFFIFRKWLPGFLEDETARLGGERYPLSSLKGDFRATCRMELDHTALGY
PKLSDFMRSLPGICRMCVVPVGSGPATHMVLLPPLSRPKYVPLLEPCSFDRDELAESVSDHHSPRS
PLNANIADDSPHNTDSQQGDDACSETNVQILQVDDACSETCVQSQHGHESSRSSAESQQDGSSS
DNGSLLDDVPVSTTKLDLIESVTSVKPALIESVPRRNPDLTESVPPRKPDLVESVPARTNLMEYGFL
PQKNESGPILKPNLLQFGPSRKLELIESKPSECFIDRPFERPAVTPSSCESDMRFSFFQSQWDKYLTPY
PRSDCCIICRSCEAGMQLVPCLHKICVACMMRCNIRACMVCGTSVSGVKSSPAVDATYRYMGLT
ERALDQRCQLMVVCRGAEAIVRCSPCMHTIACRGCFLASVTLLKSCTTCGFMVEHFKIV

>SbANK10

MPPSPLEVAVRAAVAGDLRLIKEMATTMDLRRGMGRNGRNMLHFSATAGVLDFCRFLVEEAGF
HPNSVSAEGETPIFTATEADGGGAAIVSVVRYLIERGGDPAKPDAKGCTPLHKAAEFGHVEVVRL
LLSKGVPVDPIERRGTPLHLAAVTDNHQSLKLLLDHGADAGADVNFVSPTGRTVLFNVVEDGLI
DFVKFLLEAGADPNIPDGNGRLPIMLAATREKRELVEILLPQTKPIPFVPDWSVDGIIRAMKLIQSS
EPRELVEKRLADAKAQGKEAFADGHYVAAAYYYMRAKEIDPNDATVFSNLSLCWLRLREGEQA
LAYARQCKALRPDWSKAWYREGMALSFLENYEGAVNALHEALKLDPASNEIKEALREAVEVHL
NSLLDGEDP

>SbANK11

MAPPLVGCKLHRHRLLQAAAHGDLRRFKRIASALDGGKGRIREAVEVVVDGNGAGALHVAAA
HGRTRVCAYLVEELQMDVNDTAVLSGDTPLTYAVRSKKNVDAVRYLLDHDADPNKRVHHGCT
PLSIAVGKGICVMVELLLSKGADVNSLSCGGTPLHVAAMENHGAVMKILLDYNADCNKEFFILF
TPLFTALRARSLNCVKHLIKAGADVMKGIHKFTPLILAAYEGLTDFYECLLEAGADPNVPDDSGL
LPIEIAAFQNRRKDVEILFRVTSRIPSVHDWSVDGVLTFFKYVKSFLTDQDDPMYKMDLADLKLEG
SKAYNEKDYSTAVKLYSVAIMKADSGDATLLSNRSLCFIRMGEGDKALVDAEACRALQPYWPKA
CFRQGAAHMLLKDYEKACDAFLDGLKLDPANDEMENALREALNSFKISRAT

>SbANK12

MAAPISWSDLVEALMAAPTMAASTISWMDLVEAAVSTGNLHRLKEIETASGMDLRQPTSTRGRN
LLHLAVMGACVTGWMGVCRYLVEEKGFDANSTSTEGETPIFVATESNKDGVVPLLTYFLGRGGD
PAAPDARGCTPLHNAAEYGHSEAIGLLLSRGIPVDPLSYHGTPLHLAAGKGHAQALKVLLEHGA
DPNRVVNHVSTPLLMACFAAKLECVKLLVQAGANVNFQRSPGPTMLLAAVSVGSAAIVKFLLE
AGADPNVHDGCGKSPIMLAASHKQRELVEILLPRTNPIPSMPDWSVGGIIGTMKTLPRGLAEDPM
ANVKSEGKEAFANGDYGYAIHCYLRASTIDPHDATILANQSLCWLKLREGERAVTSAQQCRRLR
PDWAKAWYREGAALSLLQKYKDAADVFLEALKLDPGNDEIENALREAMRHRNGGDAGENP

>SbANK13

MPRMDKDLLQAAIYGDSRKLQNMVAEKGLDVLLGTTQAGNTCLHIASIHGHREFCMDVLHQN
RSLITAFNDDGETPLLAAITRGHTSLASFFLHFCLNHGHRDAIFDHRELAMELIRAVPEMSRAVNS
YDESPMFISVMRDYEDITKLLLEIEDSAHGGTSGFNALHAAVRNGNSGIAKMIIEKRPDLSTEENG
YRNTAVEQAVFLNKIDVLKVLLEHDWSLGYSVSTSGSPLLVSAAQRGYVGVARELLKHCPDAPY

CDRNGWTCLHEAVLSGHTEFVEFVMGSQQLRNKLVNMRSEVGKTALHYAVEKCNPRILQALLL
NHTTTDFTMISSYGREPSSGLLTEKEPPNKISDWNEVSMVLLQKDPNNASFVYYIHKCVKDMVTN
ASSKAFKSLTQTLIGNTFLVAILIATITFAAAFTLPGGYNSEGLPTMARKAAFQAFLISDTLAMCSSL
AVAFICIIARWEDIGFLLYYRSFTKKLMWLSYMATTTAFATGLYTVLAPRLLWLAIAVCTLPVLLPI
FTKLLDELPFLSLRLRLGRTFKSELLDIV
>SbANK14
MAPASKPSDSYVVLQAALDGNLRLLKMMARKVNLREAKGDKGRNALHAAAAGDHLDVCRFL
VEELGLNVNYTTEEGMSPVHFAATAGSMCVLKYLLECGVDPAMPDSSRGCTPLHCAAEEGRCE
AVRLLLSKGIDVDPVNYRGTPLHLAVSKDQDQVVKILLEHGADPNKVVNHVFSPLMVACCAHS
LKCMKLLVEGGADVNLITPYGQTILTEAVDDGLTEIVKFLLEAGADPNIADEDGKIPIMCAAGHA
QRELIEMLFPKTRPIPSVPDWSIDGIIRSIDSLCFKTRDEALLEEQIADVKLQGKEAFAKGEYLTAIYF
YCQAMNRDPFDATLFANRSICWLRQREGDRALLDAQQCTMMRPDWSKGWYREGAALAFLKD
YKRAVVAFERALKLDPASDEIKKALREATDAMKSAAARTEQQNP
>SbANK15
MALSLSVLAIINAALDGNLGVLKQMASQTDLREAKDNVGRNALHFAAMKGHLEVCSFLVEELG
IDVNCTNTDGVTPVKYAAATGEVGVLRYLLDHGGDPEMPDARDTTPLHFAAEQGHCEALRLLL
SKGVDVDALHCRRLSPLLFAVGKGHDQAVNVLLEHGADPNIVAHNRFSPLIMACIGGSLKCMRL
LVEAGADVNLKNHYGTTALIIAVQGGFADIVRFLLEAGADPNISDANGKIAIMYAANKRLRDLV
QILFPHTKPITSLPNWSIDGIIDTMTSTSFKSMDKEHIIAGAKPRGNEAFAKGDYLAATLCYDMALL
EDPLDATLFSNRSLCWLRLGDGKLALLDAQQCKAMRPRWPKAWYREGAALSLLKDYKGAVNA
FLDALMLDPANDEIKTALREATEALSAACSEEQNP
>SbANK16
MTEPSDAAAAAAVSARLLEVAADDDSAALGDLIAAHPSLADEPAPWYSPARGAEPMTPLMVA
AAYGSVACIDVLLSPPHLADPNRASPSSLSTALHLAAGGGASTAPAAVSRLLAAGADPTLLDHL
HRRPSDLVALPPNSLPLKNHLLSLLGGRKEWPPDPSLPDIKNGAYASDDFRMYSFKVRACSRAYS
HDWTECPFVHPGENARRRDPRKYHYSCVPCPEFKKGAGCRRGDMCEYAHGVFESWLHPAQYR
TRLCKDGVGCARRVCFFAHTPEELRPLYVSSAGSQRSAMEMAAAMGMGLPSPGASFTPPLSPSG
GGSGVAGAWPQPNVPALCLPGSAGNLHLSRLRTSLSARSRAVDELLASADYDGLIGSPASVRSAR
GKTLVPSNLDDLFSAEMAGSAASHSPRYGDQGGAAFSPTRKAAMLNQFQQQQSLLSPRATVIPE
PVSPMSSRLLAALAQREKMQQQTLRSMSSRDLGSGASVLVGSPVTSSWSKWGIPSSTPDWGADD
DEIGRLKRSSSFELRNGANGDEPDLSWVNTLVKEPTPEKPSIASLSQATSHEDIGGEDDTAGVIGG
WLEQLQLDEMVV
>SbANK17
MAFWITTSSDFPEALVAAKDGDLCLLKEMAGKVNLRETKDAKGRNALHWAAVNGHLESPVHS
AASGRRESVLRYLLDRGGDPVRPDLRGTTPLHFAAEEGNCEAVRLLLSKGVDVEPVNFMGTPLH
LAASQGQDQVMKILLEHGADPKRVVNYVFTPLLMACNGPSLKCAKLLVEAGADVNFRSPSGPVI
LMEAVDDGFTEIVKFLLEAGADPNIADEDGRIPIMCAGAHGKRELVEILFPWTKPIPSMPEWSVD
GIIRGMRYLRFEAQGAVLEEHIADAKSSGKEAFAKGEYFAAVYFYGRALDRDPLDATLLANISLC
WLRVRDGKQALSFAQKCRMMCPRWSKAWYREGAALRFLKHYRGAVDAFKQALKLDPASNEIK
NALGEATDAMRNTTRS
>SbANK18
MAALSAVALQAALSGSLRLLKLVASKMNLREAKDDDGWNALHLAAEMGHLHVCRFLVEESGF
DVNSTCAEGRTPIHCAAAGGSESVLRYLLDRGGDPAMPDFVGSTPLHDAAEEGHCAAVRMLLS
KGVDVDIHGCCGTPLHLACSNDRDQVVKILLEHGADPSKVVGHVLPPLTMAYCGGSLRCMKLLI

EAGADVNLIAPSGVILAQAVCDGRTDIVKFLLEAGADPNIPDEDGRIPIMYPAVNGNRELVEILFP
KTRPVPSLPDWSVDGIIRSIEYPFFKPQDAAVVEKKIADAKSQGKEAFAKGEYLAAEYFYTMVLEN
DLDATAFANRSLCWLRMRDGAKALLDARQCKMIRPRWSKAWYREGAALSLLKDYKGAANSF
MEALKLDPASDEIKRALREATDALANVGCSGEQNP
>SbANK19
MPRQEVSLPDAFNLVLGKDWDNWPPEAGLLVAAYYGDIRRLKEIAKIIDTEGKGLHDTLANTHF
LGTYALHAACDSGCMPVLQYLVKDLKIDINKADTMRGFTPVMHAVLYGNLPALRFLVDHGAD
VHGQHKGLSLFHSAAEGGRSEIAKFLLSKGVRIDAESSLLTPLLIATYRGYPSIVEILLERGADPNER
MNDEVSPLIIALKHSSMPCLKLLVQAGADVNGFGSYSPLAKAAEKGLTEAIKCLLDAGANPNVP
DTFGRLPIELAAEYGTWEDVELLFPVTSKIPTVADWSVNGVISHVYMEVMQLEDDEFVKKKKSEL
KRQGADAFSKEDYLNASVFYTQALKVDQFDATLFSNRSLCWLRLGDGKKALLDAMKCKNLRPK
WGKAYYREGAALMFLKDYDSAYDAFNRGLGFDPESEEMEKLLWEAMDLKTT
>SbANK20
MEAVLRHPSLSRLKPPNPNLPRTPSPSLPPPSLLRLRARRLIAAAVFQDQKPKEPASKGGDDEEAY
GEVDRIVSSRTVSSPVFAEDGSASAAVATEYLVEWKDGHEPSWVPKEAIAADVVAEYETPWWTA
AKKADAEALAALLADETLRRDPDAEDAQGRTAAHFAAGLGSEECLRALAAAGADLGHRERAG
GGLTPLHIAVGYGRAGAVRALLELGADPEAPDGQGRTPLELVQEVLARTPKGNPAAFQLRQGLE
AAQKELEKAVYEWAEVEKVIDGRGEGKWREYLVEWRDGGEREWVKAAWVAEDLVSDFEAGLE
YAVAEAVVDKRQVATATAEGENRWEYLVKWVDIEEATWEPAENVDAELVQEFELRQSGSAGG
D
>SbANK21
MELPQPRPRGGGVDKYDFYYWLGCQILVLGDSIKGKLVEAIQDGDVRRLKGLVSSMKKNTRAKL
AKMNIDRHGLLQLAVFLGKLEVCRYFVEDLSFDVDCSGLCNGATPLGCASLFGEVACARFLLDH
GADPNKMDETGSVALHNAAKNGNGELMRLLLSRGARVDIAIPQGTPLHIAASYGNIDAVKILLD
HHADPDIISEVSGSPLVSALQSTKLVNESDSLECVKLLIKAGADVNSANPNTPLEVATVNGLTDCI
KCLLEAGADPNIAKNQVEKNGKTTKLKLKLDGDKAVRRKNYLAASKLYTQAIELDLDDATLYA
NRSLCHLRIGEANKALLDANSCIKIRPEWLKGYYRKGVALMSLREYKEACDAFLAGLKLDPTNV
DMERMFREAIEAMKKDHVTT
>SbANK22
MELPQPRPRGGGVDKYDFYYWLGCQILGTSCHLPRRRSVLTASGFGCSILHPVLGDSIKGKLVEAI
QDGDVRRLKGLVSSMKKNTRAKLAKMNIDRHGLLQLAVFLGKLEVCRYFVEDLSFDVDCSGLC
NGATPLGCASLFGEVACARFLLDHGADPNKMDETGSVALHNAAKNGNGELMRLLLSRGARVD
IAIPQGTPLHIAASYGNIDAVKILLDHHADPDIISEVSGSPLVSALQSTKLVNESDSLECVKLLIKAG
ADVNSANPNTPLEVATVNGLTDCIKCLLEAGADPNIAKNQVEKNGKTTKLKLKLDGDKAVRRK
NYLAASKLYTQAIELDLDDATLYANRSLCHLRIGEANKALLDANSCIKIRPEWLKGYYRKGVAL
MSLREYKEACDAFLAGLKLDPTNVDMERMFREAIEAMKKDHVTT
>SbANK23
MTPPSIFHHHHRLLPLPSASPTQILNPFAASSRRLNHHLPKPKVLSLPPRPVFSSAFAVAAVGDDE
DVVVGDCLVFDDDAFEEQDLDILSSPPPPTSASRQDWRAEAGEGESLVPERWRDAEEEINLTKKE
KRRIAHGLRFGSRLERRAPPAVAAPDEFRAYRKGMLSAEREHVAHVYRGPLERALPSEVEEPPPP
EPGTRVTPRNPRMGMDVGSLEDIDEFFRSREYVQDEMEDSKSPKGRHKLFSNEEKVLLNKRVPNL
EAATSSKWLPLHTLAASGDFYLLDSLLKHNVDINAVDKDGLPAIHKPILSKKAAIINYLLRNSANP
FIQDKDGATLMHYAVQTACSQTIKTLLLYNVDINRPDDCGWTPLHLAVQTQRTDIVKLLLIKGA
DRTLKTQDGLTPLELCLRLGHDVRAYELIKLLKSFRRQKQHDLV

>SbANK24
MASAEARRLAVVPQLEIEQILKEAQHRWLRPAEICEILKNYRNFRIAPEPPNRPPSGSLFLFDRKVL
RYFRKDGHNWRKKNDQKTVKEAHERLKSGSIDVLHCYYAHGEENINFQRRTYWMLEEDYMHI
VLVHYLETKGGKSSRARGNNNIIQGTAVDSPVSQLPSQTMEGESSLSGQASEYEEAESADIYSGAG
YNSFTWMQQHENGTGPVTNSSVFSSYTPASSVGNYQGLHATQNTSFYPVNQHNSPLILNGSSAM
LGTNGRANQTDLPSWNSVIELDEPVQMPHLQFPVPPDQSATTEGLGVDYLTFDEVYSDGLSLKDI
GAAGTHGESYLQFSSATGDLSATENSFPQQNDGSLEAAIGYPFLKTQSSNLSDILKDSFKKTDSFTR
WMSKELPEVEDSQIHSSSGGFWSTEEANNIIEASSREPLDQFTVSPMLSQDQLFSIVDFAPNWTYV
GSKTKILVAGSILNDSQINEGCKWSCMFGEVEVPAKVLADGTLICYSPQHRPGRVPFYITCSNRLA
CSEVREFEFRPTVSQYMDAPSPHGETNKVYFQIRLDKLLSLGPDEYQATVSNPSLEMIDLSKKISSL
MASNDEWSNLLKLAVDNEPSTADQHDQFAENLIKEKLHVWLLNKVGMGGKGPSVLDDEGQG
VLHLAAALGYDWAIRPTLAAGVNINFRDVHGWTALHWAAFCGRERTVVALIALGAAPGALTD
PTPDFPGSTPADLASANGQKGISGFLAESSLTSHLQALNLKEANMSQISGLPGIGDVTERDSLQPPS
GDSLGPVRNAAQAAAQIYQVFRVQSFQRKQAAQYEDDKGGMSDERALSLLSVKPPKSGQLDPL
HSAATRIQNKFRGWKGRKEFLLIRQRIVKIQAHVRGHQVRKHYRKIVWSVGIVEKVILRWRRRGA
GLRGFRSTEGSVESSSGGTSSSSIQDKPSGDDYDFLQEGRKQTEERLQKALARVKSMAQYPEARDQ
YQRILTVVSKMQESQAMQEKMLEESAEMDFMSEFKELWDDDTPIPGYF
>SbANK25
MASAEARRLAVVPQLEIEQILKEAQHRWLRPAEICEILKNYRNFRIAPEPPNRPPSGSLFLFDRKVL
RYFRKDGHNWRKKNDQKTVKEAHERLKSGSIDVLHCYYAHGEENINFQRRTYWMLEEDYMHI
VLVHYLETKGGKSSRARGNNNIIQGTAVDSPVSQLPSQTMEGESSLSGQASEYEEAESDIYSGAGY
NSFTWMQQHENGTGPVTNSSVFSSYTPASSVGNYQGLHATQNTSFYPVNQHNSPLILNGSSAML
GTNGRANQTDLPSWNSVIELDEPVQMPHLQFPVPPDQSATTEGLGVDYLTFDEVYSDGLSLKDIG
AAGTHGESYLQFSSATGDLSATENSFPQQNDGSLEAAIGYPFLKTQSSNLSDILKDSFKKTDSFTR
WMSKELPEVEDSQIHSSSGGFWSTEEANNIIEASSREPLDQFTVSPMLSQDQLFSIVDFAPNWTYV
GSKTKILVAGSILNDSQINEGCKWSCMFGEVEVPAKVLADGTLICYSPQHRPGRVPFYITCSNRLA
CSEVREFEFRPTVSQYMDAPSPHGETNKVYFQIRLDKLLSLGPDEYQATVSNPSLEMIDLSKKISSL
MASNDEWSNLLKLAVDNEPSTADQHDQFAENLIKEKLHVWLLNKVGMGGKGPSVLDDEGQG
VLHLAAALGYDWAIRPTLAAGVNINFRDVHGWTALHWAAFCGRERTVVALIALGAAPGALTD
PTPDFPGSTPADLASANGQKGISGFLAESSLTSHLQALNLKEANMSQISGLPGIGDVTERDSLQPPS
GDSLGPVRNAAQAAAQIYQVFRVQSFQRKQAAQYEDDKGGMSDERALSLLSVKPPKSGQLDPL
HSAATRIQNKFRGWKGRKEFLLIRQRIVKIQAHVRGHQVRKHYRKIVWSVGIVEKVILRWRRRGA
GLRGFRSTEGSVESSSGGTSSSSIQDKPSGDDYDFLQEGRKQTEERLQKALARVKSMAQYPEARDQ
YQRILTVVSKMQESQAMQEKMLEESAEMDFMSEFKELWDDDTPIPGYF
>SbANK26
MDEWLPLPNVFGRLLDTNDQDQWPPEATLLIAANDGNVRRLKEIARGLDVNGRGINSVLRRTTF
KGMDALHAAAGGKGKLPICRYLVEEAKMDVNKRDTFKGKSMTPLQHAVFSGNLPIVRFLLDHG
ADLHQEGSLGGHERCTALHTAAEKGRCEIAKLLLSRGAYVDGKSCHMTPLQLAVHGGHDSTLK
ILLDHNADPNKEINLSTPLTIALCTPSLSCLKLLIQAGAEVNEIGNPLARAAREGLTEAIKCLLEAG
ANPNTTDCYGRFPIELAAVYGTREDVEILFPCTSPIPTVTNWSIDGIINHMELERKQREDDDFVEAR
KSDLKRQGGDAFKKQDYINSSAFYTQAVKVDQYDAGLFSSRSLCWLRTGDGKRALGDAVRCKM
LSPKWSKGYYREGQALILLKGGNGSEE
>SbANK27
MDEWLPLPNVFGRLLDTNDQDQWPPEATLLIAANDGNVRRLKEIARGLDVNGRGINSVLRRTTF

KGMDALHAAAGGKGKLPICRYLVEEAKMDVNKRDTFKGKSMTPLQHAVFSGNLPIVRFLLDHG
ADLHQEGSLGGHERCTALHTAAEKGRCEIAKLLLSRGAYVDGKSCHMTPLQLAVHGGHDSTLK
ILLDHNADPNKEINLSTPLTIALCTPSLSCLKLLIQAGAEVNEIGNPLARAAREGLTEAIKCLLEAG
ANPNTTDCYGRFPIELAAVYGTREDVEILFPCTSPIPTVTNWSIDGIINHMELERKQREDDDFVEAR
KSDLKRQGGDAFKKQDYINSSAFYTQAVKVDQYDAGLFSSRSLCWLRTGDGKRALGDAVRCKM
LSPKWSKGYYREGQALILLKEYEKACDTLSEGLELNPMCDELDKLYWEAMDLKNSSTGAA
>SbANK28
MADARKFVLPGQPPDISQILQEAQKRWLRPTEICEILSNYKLFSIAAEPPNMPRSGSLFLFDRKVLR
YFRKDGHNWRKKKDGKTVKEAHEKLKAGSIDVLHCYYAHGEENENFQRRTYWLLEEDFTHIVL
VHYLEVKGCKQSFNRVKEEFMQLSNVDSPSCSNSITSQNQMGPQNMEADESPISGQISEYEDTESD
NCRASSRYHPLAEMQQLVDGVITENMLYPSASTVDSRQGYHGEMLPITDNLDSRSFSHHDIARM
LDGANIGLSDVSSTPFDSVPFNEPFTNYSAGFREPTLHSSFACLEANNLDDSSCLQTFTSEALYTNH
LSQKEADALGFTGILTSEVNGDRYNDDSIKHPLLKQSSLDLLTIETPGLKKHDSFSRWMSKELEEV
VDLGIKSTSDAFWSSIETVKVPDGSNVLSNEQLGAYVVSPSLSQDQLFSILDVSPSCAYIGTNTKVSV
TGTFLVNKEHVENQKWSCMFGDVEVPAEVLTDGTLRCYAPAHQSGRVPFYVTCSNRVACSEVR
EFEYRDSDAQYMETSRSQANGVNEMHLHIRLEKLLTLGPDDHHMLAISSGNEKYEIVNAINALM
LDGKWSNQESSVKEVVSTARGQSIKKLVKEKLHQWLICKVNDDGKGPNVLCKEGQGVIHLVAA
LDYDWAIRPIMVAGVNVNFRDAHGWTALHWAASLGRERTVSVLIANGAAAGALTDPTSEFPSG
RSPADLASVNGHKGIAGFLAESALTSHLSALTIRESNDSTVEVCGLPVAEDLTGTDSAQLAGEGPH
AESLEGSLSAVRKSTQAAARIFQAFRVESFHRKKVVEYGDDDCGLSDERTLSLVSLKNVKPGQHD
THLHSAAVRIQNKFRGWKGRKEFMIIRQRIVKLQAHVRGHQVRKNYRKVVWSVGIVEKVILRWR
RKRPGLRNFQPQKQLEGPSQIQPAKAEDEYDFLHDGRRQAEARLQRALARVHSMSQYPEAREQY
HRLTTCVAEMKQSRV
>SbANK29
MADARKFVLPGQPPDISQILQEAQKRWLRPTEICEILSNYKLFSIAAEPPNMPRSGSLFLFDRKVLR
YFRKDGHNWRKKKDGKTVKEAHEKLKAGSIDVLHCYYAHGEENENFQRRTYWLLEEDFTHIVL
VHYLEVKGCKQSFNRVKEEFMQLSNVDSPSCSNSITSQNQMGPQNMEADESPISGQISEYEDTESD
NCRASSRYHPLAEMQQLVDGVITENMLYPSASTVDSRQGYHGEMLPITDNLDSRSFSHHDIARM
LDGANIGLSDVSSTPFDSVPFNEPFTNYSAGFREPTLHSSFACLEANNLDDSSCLQTFTSEALYTNH
LSQKEADALGFTGILTSEVNGDRYNDDSIKHPLLKQSSLDLLTIETPGLKKHDSFSRWMSKELEEV
VDLGIKSTSDAFWSSIETVKVPDGSNVLSNEQLGAYVVSPSLSQDQLFSILDVSPSCAYIGTNTKVSV
TGTFLVNKEHVENQKWSCMFGDVEVPAEVLTDGTLRCYAPAHQSGRVPFYVTCSNRVACSEVR
EFEYRDSDAQYMETSRSQANGVNEMHLHIRLEKLLTLGPDDHHMLAISSGNEKYEIVNAINALM
LDGKWSNQESSVKEVVSTARGQSIKKLVKEKLHQWLICKVNDDGKGPNVLCKEGQGVIHLVAA
LDYDWAIRPIMVAGVNVNFRDAHGWTALHWAASLGRERTVSVLIANGAAAGALTDPTSEFPSG
RSPADLASVNGHKGIAGFLAESALTSHLSALTIRESNDSTVEVCGLPVAEDLTGTDSAQLAGEGPH
AESLEGSLSAVRKSTQAAARIFQAFRVESFHRKKVVEYGDDDCGLSDERTLSLVSLKNVKPGQHD
THLHSAAVRIQNKFRGWKGRKEFMIIRQRIVKLQAHVRGHQVRKNYRKVVWSVGIVEKVILRWR
RKRPGLRNFQPQKQLEGPSQIQPAKAEDEYDFLHDGRRQAEARLQRALARVHSMSQYPEAREQY
HRLTTCVAEMKQSRMMQDEMLSEAAGGGVDDFMAGLEDLICIDDAAMSAIW
>SbANK30
MADLEIGPASPVSEGAPSPASSAGASGERPDQSPSLLAKRPGLVMSFSGKRLDQSPGGSPSQSRPVL
VMSHSSNRLDQSPARPVLVMSRSSNRLDQSSPASSPARVRGPVLVMSGSSNRLESSSPSPSPTAAA
AAAPVLVLSNSGKRMDQAGRKKYVKQVTGRHNDTELHLAAQRGDLEAVRQIIAEIDAQMTGT

GEEFDSEVAEIRAAIVNESNEMEATALLIAAEKGFLDIVVELLKHSDKDSLTRKNKSGFDPLHVAA
REGHRDIVKVLLDHDPSLGKTFGQSKVTPLITAAIRGHTAVVNLLLERVCGLVELSKANGKNALH
FAARQGHVEVVKALLDADTQLARRTDKKGQTALHMAVKGTNPLVVQALVNADPAIVMLPDR
NGNLALHVATRKKRSEIVNVLLLLPDMNVNALTRDRKTAFDIAEGLPLSEESQEIKECLARAGAV
RANDLNQPRDELRKTVTEIKKDVHTQLEQARKTNKNVYGIAKELRKLHREGINNATNSVTVVA
VLFATVAFAAIFTVPGGNTNDGVAVAVHATAFKVFFIFNAIALFTSLAVVVVQITLVRGETKAERR
VIEIINKLMWLASVCTTVAFISSSYIVVGRRLKWAALLVTLIGGVIMAGVLGTMTYYVVKSKRTRKI
RRKVKSTRRSGSNSWQHNSEFSDSEIDRIYAI

>SbANK31

MAADSGKGMADLEIGPASPVSEGAPSPASSAGASGERPDQSPSLLAKRPGLVMSFSGKRLDQSPG
GSPSQSRPVLVMSHSSNRLDQSPARPVLVMSRSSNRLDQSSPASSPARVRGPVLVMSGSSNRLESSS
PSPSPTAAAAAAPVLVLSNSGKRMDQAGRKKYVKQVTGRHNDTELHLAAQRGDLEAVRQIIAEI
DAQMTGTGEEFDSEVAEIRAAIVNESNEMEATALLIAAEKGFLDIVVELLKHSDKDSLTRKNKSGF
DPLHVAAREGHRDIVKVLLDHDPSLGKTFGQSKVTPLITAAIRGHTAVVNLLLERVCGLVELSKA
NGKNALHFAARQGHVEVVKALLDADTQLARRTDKKGQTALHMAVKGTNPLVVQALVNADP
AIVMLPDRNGNLALHVATRKKRSEIVNVLLLLPDMNVNALTRDRKTAFDIAEGLPLSEESQEIKE
CLARAGAVRANDLNQPRDELRKTVTEIKKDVHTQLEQARKTNKNVYGIAKELRKLHREGINNA
TNSVTVVAVLFATVAFAAIFTVPGGNTNDGVAVAVHATAFKVFFIFNAIALFTSLAVVVVQITLV
RGETKAERRVIEIINKLMWLASVCTTVAFISSSYIVVGRRLKWAALLVTLIGGVIMAGVLGTMTYY
VVKSKRTRKIRRKVKSTRRSGSNSWQHNSEFSDSEIDRIYAI

>SbANK32

MAVPGGRNGLRDEDDEMAEHAEAFAGASDDEDVPPHLRALANAAQTGDVAALVAALDNHD
GSIDVPVEDGDTLLHLACLYGHLPCVQLLLERGASLECKDEEGAIPLHDACAGGFTEMVQYILNF
AANKDGCVARMLDTVDSEGDTPLHHAARGEHLDVVKLLIEAGASPKKENTYGQTPADMADQD
TEVRTLLTAKQIEASTHMSGN

>SbANK33

MEAKNQTGGDEECSRPQQQPVVMDSPRAAQAATRRKKMTKQLTGKRDDTAMHAAARAGQL
ASMREMMSGKDAEELGALLSRQNQAGETPLFVAAEYGYVALVGEMIRCHDVATASIKARSGYD
ALHIAAKQGDVDVVRELLQALPQLSLTVDSSNTTALNSAATQGHLDVVRLLLQVDRSLALIARS
NGKTALHSAARNGHVEVVRALLEAEPSIALRTDKKGQTALHMASKATRLDLVDALLAAEPALL
NQKDNKGNTALHIAARKARHEIIRRLVTMPDTDLKAINRSGETPLDTAEKMGNGDAAELLAEH
GVQSARAISPGAGGGNNKQQRELKQQVSDIKHEVHSQLEQTRQTRVRMQGIAKRINKLHEEGL
NNAINSTTVVAVLIATVAFAGIFTVPGEYVEDPRSSLTPGKDVGEANISHQTAFIIFFVFDSVSLFISL
AVVVVQTSVVVIERKAKKQMMAVINKLMWVACVLISVSFLALSFVVVGRAERWLAVSVTIMGTT
ILATTIGTMLYWVIAHRMEAKRIRTNKRNRLSRSRSLSISGMSEGEWVEEEYKRMYAI

>SbANK34

MEGAFDLNTRIFLSLNSRLPYGDKSPQMDFFDAAFKGDLRRVREMARGKDAEGKAQLVDVCIG
GQGPLQAAARMGRLDVVKFMVKELGFDINLGSSGTGVTALHAAALDGKLDTVRYLLDNGADP
NKKDEPGEVPLHCAAKYGHDEVAKLLLSRGANVDMAYFHGTPLHIAAVHGKADFMKVLLEQH
ADPNKISEALGTPLVATLNAASDGLPESIALKCVKLLVEARADVNFVGPDTPLVVATTHGLTDCI
KYLLNAGANSNIPKKCCGAIPIEIAAGYGRRKDVELLFPSTSPTQTVSVWTVDGILSHAQSKHSNP
KVEKYGKITKLELKLGGDKAVGRKNYLAASKLYTQAIELDPDDATLYSNRSLCQLQIGEANKALL
DANSCIKIRPEWLKGYYRKGVALMSLKEYKEACDAFLAGLKLDPTNVDMERMFMEAIEAMKKD
HVTT

>SbANK35
MSVSGVTALHAAALDGKLDTVRYLLDNGADPNKKDEPGEVPLHCAAKYGHDEVAKLLLSRGA
NVDMAYFHGTPLHIAAVHGKADFMKVLLEQHADPNKISEALGTPLVATLNAASDGLPESIALKC
VKLLVEARADVNFVGPDTPLVVATTHGLTDCIKYLLNAGANSNIPKKCCGAIPIEIAAGYGRRKD
VELLFPSTSPTQTVSVWTVDGILSHAQSKHSNPKVEKYGKITKLELKLGGDKAVGRKNYLAASKL
YTQAIELDPDDATLYSNRSLCQLQIGEANKALLDANSCIKIRPEWLKGYYRKGVALMSLKEYKEA
CDAFLAGLKLDPTNVDMERMFMEAIEAMKKDHVTT
>SbANK36
MGNSLGCSASGERLVSAARDGDAVEARMLLELSPALARYSTFGGLNTPLHFAAAKGHLDIVTML
LEKGADVNARNYCGQTALMHACRHGHWEVVQMLLLFRCNVTRADYLSGRTALHFAAHDGFV
RCIRLLVADFVPSVALEDIASSVVDGGDCQTNSGSSPNSLLGQKFNESARVRYINKPADGGVTAL
HMAALNGHLDCMQLLIDLGANVSAVTFPYGTTTNLIGAGSTPLHYAAGGGKQECCELLISKGAS
RLTLNCNGWLPVDVARIFGRRSLEPLLSPNSHSNVPVFQPSSYLALPLMSILNIAREFGLQHTVPSA
DDNDLCSVCLERSCSVAAEGCSHEFCIKCALYLCSTSNIRVEFTGPPGSIPCPLCRNGIMSFTKLPST
PTEGLKSSSALTFCNPCILNTRSMDSPATVSKAEIRRNRVAAVSSELVCPITCSPFPSSALPTCRCSD
DDPCGATDAQDGSEAQSPRPSHCVSMELDKRGEEDLDRTSCSGMFWSRRSCHREQQCDAEINA
>SbANK37
MAAQEEKTATAVTTEEPAPAAEPQPKPAAAARRAGPSASANPFDFSTMMNLLNDPSIKEMAEQI
AKDPAFTEMAEQLQKTVVSPRQRQQQAASAAAAAQLDPQKYVATMQQLMQNPQFVAMAE
RLGSALMQDPAVSTMLGGLTNPAHKEQLEARVARMKDDPDLKPILDEIESGGPAAMMKYWND
PEALQKFGRAMGVGGGPSGEAGGAEHAEAEDDAGEEGEYEDESIVHHTASIGDVEGLKKALED
GADKDEEDSEGRRGLHFACGYGELQCAQALLEAGAAVDAVDKNKNTALHYAAGYGRKDCVA
LLLESGAAVTLQNLDGKTPIDVAKLNNQDDVLKLLEKHAFV
>SbANK38
MAAWSSREHDKQMAASLRMIASAMGGTGISSQTLDEMKRTVAKADCDASNVVEVLERMAEDS
DEEEHLGPLHDAAGAGKMDVCKHLVENLGFDVDVRANDGSGKTPLTCAVSRGKVIAVRYLIAK
GADLNKQDAMGFTPLHYAVKKGYSGIARLLLSRGANVDVESSEGTPLHVSAARGKFGVMEILLE
HHADGHKTPIEIAAKSGRRNLVESLFPFTSPIQTISDWSVEGILAHARSRQSKHKGERSDNDSKIQL
NLNGERSVKEDAGALKGCTEDKLSDEDRKAQLKLHGGQAVKGKDYAGASKFYTEAIMLDPAD
ATLYSNRSFCHLKIGGARDALVDANACISLQPDWPKGYYRKGSALMSLKEYKEARDAFMDGLRL
DPSNLDIQNAYWEADEAMIKKHSTGQSAD
>SbANK39
MAAWSSREHDKQMAASLRMIASAMGGTGISSQTLDEMKRTVAKADCDASNVVEVLERMAEDS
DEEEHLGPLHDAAGAGKMDVCKHLVENLGFDVDVRANDGSGKTPLTCAVSRGKVIAVRYLIAK
GADLNKQDAMGFTPLHYAVKKGYSGIARLLLSRGANVDVESSEGTPLHVSAARGKFGVMEILLE
HHADPDRVSPDLCTPLAIILCATYEKASLSACLKCMKLLVKAGADLNSTNPDTPLVIATSRGLNE
CIDYLLEVGANANIQIKHGHKTPIEIAAKSGRRNLVESLFPFTSPIQTISDWSVEGILAHARSRQSKH
KGERSDNDSKIQLNLNGERSVKEDAGALKGCTEDKLSDEDRKAQLKLHGGQAVKGKDYAGASK
FYTEAIMLDPADATLYSNRSFCHLKIGGARDALVDANACISLQPDWPKGYYRKGSALMSLKEYK
EARDAFMDGLRLDPSNLDIQNAYWEADEAMIKKHSTGQSAD
>SbANK40
MNGMPISASSAAGVDGGAVAAGTPMKNATAAAAVAEMARHLTVDTEDAFAGLLEFAADDNA
EGLRRALERAPPAAADEAGLWYGRRKVLEHRTPLMVAATYGSLAALRLLVSLPSVDVNRRSGTD
GTTALHCAASGGSRTAVEAVKLLLGAGADADTMDDAGRRPADVISVPPKMFDAKFALQDLLGF

PKSEHGMLRVVTRSTNSISSPVSSPTAEDARSPSASVMMISKFGDLPRVATSEKKEYPVDPSLPDIK
NSIYASDEFRMYSFKIRPCSRAYSHDWTECPFVHPGENARRRDPRKYHYSCVPCPDFRKGVCRRG
DMCEYAHGVFECWLHPAQYRTRLCKDGTSCNRRVCFFAHTTDELRPLYVSTGSAVPSPRASATA
AMEMAAAMGLMPGSPSSVSAVMSPFTPPMSPSGNGMPPSLGWQQPNVPTLHLPGSSLQSSRLRT
SLSARDMPADDYSMMPDLDSQLMNDLCYSRLSSSTGNHSARTKSLNPSNLDDLFSAEMVSSPRYS
NADQGAMFSPSHKAAILNQFQQQQQALLSPINTGVYSPKADNQQLPSHSSLLQASLGMSSPGRM
SPRCVESGSPMNSHLAAALVQREKQQQTMRSLSSRDLGPSAARASALGSPLSSSWSKWGSPSGTP
DWGVNGEELGKLRRSSSFELRSGGDDPDLSWVHTLVKESPPEKQVTTAESINSVGPSPLMPPGMN
SGEGSGLNTQLDAHDQAAVIGALLEQMQLDQQIGSLAT
>SbANK41
MPREEAMQEYITIVQELFPNWDAGASAKGKDEDSIASARGPRDLSLMYEEEEGNDSELGDIHVSA
RERANDDIVKPLSDGVEVNARDTEGRSPLHWAVDCGHLSAVEALAKANADLNAKDNEGQTTL
PCAAVCEREDIASC
>SbANK42
MEAPFEFKMRTILCLNRNLPYGTTGPDMDFFEAAFEGDIPRLREMASGKDAEERALLADVCFDGL
GPLQVVARMGRLDVVRYLVEDLGFDVNHGSAQFGLTALSSAALDGRLDTVRYLLDNGANPNLQ
DELGQVPLHCAAKYGHAEVTELLLSRGASVDLTYFHATPLHIAAVYGKASVMKILLEHHADPN
MVSEVLGTPLVATLHATTEGLEECISLKCVKVLVEAGADVNSTDPDTPLVVATTHGLTDCIKYLL
KAGANANIPNSCCGAMPIETAASCGRRKHVEMLFPFTSPIQTVSKWSVDGIISHVKSKRSKYSKHS
KHSKPKHEESTEVKVKLDGDKVVGRKNHLATSKLYGEAEQYDKSMKDELKLNGDKAVGRKDY
LTASKFYGKAIELDPADATLYSNRSLCLLQIGEATGALSDASTCIKMRPEWIKGYYRKGTALMSLK
EYKEACNAFMAGFKLDPSNAVMERMFWEAAEAMKKDHVGTKDLESID
>SbANK43
MAEARRHAIAPQLGGSLFLFDRKVLRYFRKDGHNWRKKRDGKTVKEAHERLKSGSVDVLHCYY
AHGEGNENFQRRSYWMLEEDFMHIVLVHYLEVKGGKSTSRIRGHDDMLQAARTDSPLSQLPSQT
TEGESSLSGQASEYEETESDIYSGGSGYHPFSWTQQHENGGGPVMGASIPSSYIPALSLGNFQGFPA
TVTNTDIYSYCQDALPGALNEPGLGVGFNGADNQVDPSSLNGLVKPDQGILQMSPPQSTVPSELF
PFAEGHGIESFTFDEVYGNGLSIKDADVIGTDEESVWQLPGAISSFPPEDSFQQNDRSLEENINYPLL
KTQSSSLSEMLKDSFKKSDSFTRWMSKALGEVDDSQIKSSSGVYWNSEETDNIIEASSRDQLDQFTL
DPVLAQDQLFSIDDFSPSWTYAGSKTRVLITGRFLNSNEIQRCKWSCMFGEVEVPAEVSADGTLRC
YSPSHKPGRVPFYVTCTNRLACSEIREFEFRPSVTQYMDAPSPHGATNKTYLQMRLDDLLSLGHN
EYQATVSNPTKEMVDLSKKISSLMTSNDSWSKLLKLASDNEPATDHNQDQFFEKRLKEKLHIWL
VHKARDGGKGPNVLDDEGQGVLHLAAALGYDWVIRPTVSAGVNINFRDAHGWTALHWAAFC
GRERTVVALIALGAAPGALTDPTPDFPTGSTPADLASANGYKGISGFLAESSLTSHLQTLDLKEGK
GSNAPEISGLPGIGDVTERRASPLAGEGLQAGSVGDSLGAVRNAAQAAARIYQVFRVQSFQRKQ
AVQYEDGNGAVSDDRAISLLSVKPSKPVQLDPLHTAATRIQNKYRGWKGRKEFLLIRQRIVKIQA
HVRGHQVRKHYRKIIWSVGIVEKVILRWRRKGAGLRGFRSTEGAMEGNSSSSSNLIQNKPAEDDY
DFLQQGRKQTEERLQKALARVKSMAQYPDARDQYQRILTVVTKIQESQAMQEKMLDESTEMDE
GFFMSEFKELWDDDVPMPSWS
>SbANK44
MEVSTISFSSPSSSPPSLSPSPPPSQQQQHQPRGATPADLEAVGLRRLSDNLQRLLDPAFLNCADAE
IALAPAKGGGAVGVHRCILAARSAFFLHHFASLPAPAGGGGERPRLELADLVPGGRHIGQDALV
PVLGYLYTGRLKSPPQEATVCMDDACGHGTCRPAIDFVVESMYAASGFQISELISLFQRRLSDFVSE
ALDEDVVPIIHVASTCDLQDLLNQCIHRVAVSTLDSRYLEKELPDDIYCRIKEIRRSTFHDESSESAIL

DPEHDKRVRNILKALDSDDVDLVGLLLKESTVTLDDAFAIHYAAAYCEPKVFAELLKLDSANVN
RKSNSGYTPLHIACMRREPDIILSLVERGASVLERTLDGRDALTICKRLTREKDCNRKLEKYEEKSK
AYLCIDILEQELKRKSFILDPISIEESIATPLLVDNFHMRLINLENRVAFARIFFPSEAKLVMRIAQAD
STEEFAGITNFSKLKEVDLNETPTMQNRRLRERLDALTKTVELGRRYFPHCSDVLDKFLNEESTDLI
FLETGTPEDQRVKRMRFSELKEDVRKAFTKDKAAVAAIASSASSSSPRCEGRGRSNRKLRPSR
>SbANK45
MAAAMDVDRAGGGERRPSEKELFRAAESGDAAAFASLAPADLSLRNEDGRSLLHVAAAAGHP
QVVLALAQCGGDAAANVLNAKDEEGWAPIHSAASSGNSQIIDILLERGADVNLVTDGGRTALH
YAASKGRLNIAEKLIANGANVNKKDKFGCTPLHRAASTGNAELCEFLIEEGAEVDAVDKTGQTP
LMHAVICENKGVALLLIRHGADVDVEDKEGYTVLGRASNSFRPALIDAAKAMLEG
>SbANK46
MGHGASCGRPSDEVDFFGAAQAGDTARLAAALRSRPTLLTRTTLFDRLSALHIAAAHGHLQVVS
LALDLCVHPDVVNRHKQTALMLAAMHGKTDCVRRLLDAGANIVMFDSSHGRTCLHYAAYYG
HADCLRTILSAAKSAPVSESWGFARFVNVRDDTGATPLHLAARQGWRRCVHVLLENGAIVSASS
GAFGFPGSTPLHLAARGGNLDCVRQLLSWGADRLQRDSVGRIPYEVAVKRGHVACAALLNPSS
AEPLVWPSALKFISELEPNAKSLLEAALMEANRERERRILKGTKNALPSPSHPDDGAHDTAIAEAS
DAEVCSICFEQACSIEVRECGHQMCSACTLALCCHAKPNPATQSQPQPTCPFCRGGIARLVVATR
TRAGDDEDDEERGRLESPRHRRSRRSMNLSGDAGSTSTLMGSIASSIGKMGRRRTDSSEQVDVKP
>SbANK47
MAAPFVYLPSTFNGNSPDALLKAALDGNLGRIKGIIRNLGIGNGDRAAVLSFHKDGFGVLHCAA
CQGHLEVCKYLVEELGCDPNMAEGSCEGATPFMASAQSGDISTVKYLLDHGGDLMKADAKGRT
VLHHAVCAGSCKVTEFLLSKGIPVDIDCGRGTPLFHAANNEQDKTLKILLDHHANPNTIVGGGM
GTPLMSALIYRSLKCMKLLIKAGAEVNGEGSVMPPLVFATGHGGYTNFIQLLLKAGANPNIPDDL
GRLPIELAALRECKEEVEMLLPLTSPIPNVRNWSVDGVISHAKFENTKPLDKMRINRRRAMIKSQA
DLAFRQKDYALALKFYNMGIDIAPEATLYSNRSLCKLRMGDGEGALSDAYQCRMMRPDWAKA
CYRQAAAHMLLKEYKQAYDALLDAQKLDPGNDEIEKELSKAMESMNVSPDED
>SbANK48
MEAARFGTQSSHLYGGGLGELDLNRRENRVFGWDLNDWSWDSQRFVATPVPAQAANGSGLNS
SPSSSEEAEEDVARNGGLRGDTDKRKRVVVIDDDETEDQDTVVNGGGSLSLRIGGTAVGVEAME
NSDVNEDERNGKKIRVQGGGSSGPACQVEGCGADLTAAKDYHRRHKVCEMHAKASTAVVGN
TVQRFCQQCSRFHLLQEFDEGKRSCRRRLAGHNRRRRKTRPDTAVGGTASIEDKVSNYLLLSLIGI
CANLNSDNVQHSNSQELLSTLLKNLGSVAKSLEPKELCKLLEAYQSLQNGSNAGTSGTANVAEE
AAGPSNSKLPFVNGSHCGQASSSVVPVQSKATMVVTPEPASCKLKDFDLNDTCNDMEGFEDGQ
EGSPTPAFKTADSPNCASWMQQDSTQSPPQTSGNSDSTSTQSLSSSNGDTQCRTDKIVFKLFDKVP
GDLPPVLRSQILGWLSSSPTDIESHIRPGCIILTVYLRLVESAWQELSENMSLHLDKLLSSSTDDFWA
SGLVFVMVRRHLAFMLNGQIMLDRPLAPSSHDYCKILCVKPVAAPYSATINFRVEGFNLLSTSSRL
ICSFEGRCIFQEDTDFIAENAECEDRDIECLSFCCSVPGPRGRGFIEVEDSGFSNGFFPFIIAEKDVCSE
VSELESIFESSSNEHANVNDDARDQALEFINELGWLLHRANRMSKEDETDTSLAALNMWRFRNL
GVFAMEREWCAVIKMLLDFLFIGLVDVGSRSPEDVVLSENLLHAAVRRKSVNMVRFLLRYKPNK
NSKGTAQTYLFRPDALGPSTITPLHIAAATSDAEDVLDVLTDDPGLIGISAWSNARDETGFTPEDY
ARQRGNDAYLNLVQKKIDKHLGKGHVVLGVPSSICSVITDGVKPGDVSLEICRPMSAAVPSCLLC
SRQARVYPNSTTKTFLYRPAMLTVMGVAVVCVCVGILLHTFPRVYAAPTFRWESLERGAM
>SbANK49
MDSERRMNPALYKAATQGKMSSLKQLVDPEDPSVLSATTPQLNTALHLAALHGHAEFAGEVLD

MNEELLVAQNNDGDTPLHLAAKAGKLEVARLLVNRALAWPQDKKSPLIMTNKAGDTALHEA
VKYRRGAVAVVLLDADPNRGHDLNERMESPLDMAAREGLVQVVQKIVNSPWVGQEFLPGISLS
GTALHQAVLGTHHRIVEILLDKRPDLIDLTDSDGNNALHYAAQKDHQRAVEMLLKKRTELAYK
RNNKSMSPLHVAAQYGSTDTIKALLRHCPDVAEMADSYGRNAFHASVISGKANALRCLLRRVR
PAELLNRVDINGDTPLHLAAKMSRVHSALMLLNDSRVDPCVRDHDGQTARSLVERKLHTGEMD
AYEMYLWKQLRYQESKRCRKQQLPPLATYPSRRGNDKYFERIVETYILVATLIATVTFAATFTMPG
GYNQTTGIALQGHHVAFQIFVVSNTIAMCSSIVVVFCFIWAWQDPVRFKVDQLLWGHRLTVIAC
LGMLVSLMTAVYITVEPVSRWPAYVVIAIGTSTPAVVFLMLGREVIFVPL
>SbANK50
MDRLVIPEPSNEVMIRVEPGRQARGELTLRNAMHTMPVAFRLQPAVRGRFAVRPHTGILAPLAA
VTVEVLYLASVPPEGPSGSGGGGSRGDDAFLLHSVVAPGAAVKEPVTALDSVNPEWFSARRKQV
FVDSGIRASFVGASVAARLVEAGAVEALREVLDRSEPEWRAADATDESGRTLLDLAVSLGRADIV
QVLLEYGADADKPSRGRTPLETAAASGECLIAELLLANGATPAGSDAIHVAAAAGHNDVLKLLL
GKPPSASPASSSSASFSSSFTSIDAAGRDGKTPLRLAAEAGRRDAVKALLAAGARADARCGTDGG
TALHAAARRGDEAVARLLLSHGVAGTATVRDVAGKTAFEIAAEEGHGGRILDFLGLGEAILAAA
RKGEVRSVRRAADGGASVEGRDAHGWTPLMRAAFKGRADTVRDLIDRGTDMDAADAEGYTAL
HCAAEAGRADVVDLLLKNGANVKATTVKGRTAADAAAAAGKSKVVRLLDKASVMVGRKQD
VSEKVAPAVAKGGSMDRKRRARKGSSGAIRFVGGKEGFEAAAVSVGWSH
>SbANK51
METEAKRGMDPALYKAATQGCVRSLRKLVVKDVKILNSKTPQDNTALHLAALHGHPKFARQV
LAVSEELMVARNADGDTALHLAAKTGRQKVAEVLVDIARAWPDEPNSEDTLLKSPLIMTNHEG
NNPLHEAVRHRKTAVALALLDADHSRAYDPNEKMESPLHMAAREGLVHVVRKVFDFAWVEP
QYVSSAAVSGTALHQAVLGGHTKVVEIMLEKHEQLVDMTDSNGNNALHYAAQKNNSHVVELL
LHKKTQLAYSRNKDWQSPLHVAAQYGSTAVIKALLHHCSDVSEMEDGNGRNAFHASVISGKAN
ALRCLLRRVRPAELLNRADKNGDTPLHLAAKMSHVHSALMLLRDRRVDPCIRDRDDQTARSLV
EKKLHTGETDAHEMYLWKQLKRQESARCRKQQLPPVTFSGDSRTSSHKYFERSVETYILVATLIAT
VTFAATFTMPGGYYQDSGIAIHGHDTAFKIFVISNTVAMCSAIVVVYCFIWAWKDPLKFKIDQLV
WGHRLTMIAGLGMLVSLMASVYITVPHKSRWPAYVVIAIGMSTPAVVVLMLGRDVIFVPL
>SbANK52
MAAAPESKSPEELLCAAAKSGEEEEVAKLLASGADATHFDADGLTPLMHAAAGGHAAVARLLL
DCGAPWNALSPSGLSAGDLTSDPDTYDLLLDHALRSELVLGTVARRQAAPANASDGAPAESYLE
SRVSFSEERVMDAESKAVMMAWERPLMEAHARAVCQGGGKVLNVGFGMGLVDEAIQRYEPEE
HTIVEAHPQVYERMLKLGWGEKKNVRIVFGRWQDVMPQLGSYDGIFFDTYGEYYEDMREFHQH
LPKLLKPGGIYSYFNGLCGDNAFFHAVYCQLVALELANLGYSTQFIPLPVKDCLAEEVWKGVKQ
KYWQLDTYYLPACQSESESE
>SbANK53
MGRRHGGGGRGGGRGRGRGRDEEEDLPLHKAARSGDAAAVESLCESNPLAVNSRDRLSRTPLH
LAAWAGHVDVVRCLCKHKADVGAAAMDDTAAIHFASQKGHVEVARELLASGASVKAKNRKG
FTALHFAAQNSHLDLVKYLVKRGIDVTTKTKGGQTALHVAEDDEVRAFLKECEQSMKKGAELPS
EKKDDSAEKEGDSKSSGEVMKEGEDAGQGDKRKSEGSGASSSPGAKKAKVSLGHLVSENDMDE
EEE
>SbANK54
METSISPPGTSKQSAIRKPSPGSSLKDLCLVSKQGSIAEVESALALLKRSGGNIDGRNAFGLSALHL
ATWRNHLPIVRRLLDAGADPDARDGESGWSSLHRALHFGHLCIAGVLLQFGASLTLEDTKGRTP

VDLLSCPVSQANGDFPDAVATEVFSWGSGTNYQLGTGNAHIQKLPCKVDTLHGSYIKTVAASKF
HSVAVSYDGELYTWGFGRGGRLGHPDIQSGQTTAVITPRQVTVGLGRKRVNVVAAAKHHTVIA
TETGELFTWGSNREGQLGYPSVDTQPTPRRVSSLKQRIISVAAANKHSAAVADTGEVFTWGCNKE
GQLGYGTSNSASNCMPRMVEYLKGKVFRGVSAAKYHTIVLGVDGEVFSWGHRLVTPRRVVVAR
CLKKGGNTNLKFHRMERLQVISVAAGTTHSTALTADGALFYWVSSDPDLKCQQIFSMCGRDIVN
ISAGKYWTAVATSTGDVFMWDAKKRKDEMPIFTRVHGVKRATSVCVGETHMLVLSSIYHPEYPP
KPKSQGRKSMLEWNSGTEELDEDILFNDVQPNSGLSGSSGEMSKGVPSLKSLCEKVAVEYLLEPK
NAIQLLEVADSLEAKELKKHCEDLAIRNLDYIFTVGAPSIMNASPEILVNLEKLLDEKSSEPWSQRR
LPTMTATYPAVIDSDVEEDEREFPKPRKCGKSASRPSAMSSHDNFLQKDCTAEQAVTKQIRALRK
KLQQIEILEAKQLAGHQLDNQQLAKLESRAALEGELSELGVPSEAYSTSSACPAEGRANRKPEVSK
KQKRKNKQATQTLSVKSEPGQQIPIKDLQEVLSVNVSAEKEAYAADPIKHIEAAAFSNTKDIASPL
EKKASQPTSSKKKNRKGGLSLFLSGALDDTPKPSLPAPVVPVTPKPEGPAWGGAKITKGPASLRDI
QSEQSKTNEPTSAKAKDRYENSPDSAGRVRLSSFIPDSHSSPIAVTPARAVPSSEGDKSTPPWSSSAT
SPNVSRPSLRDIQMQQEKRHHGISHSPKTHTSGFALPHHGGSPEVGGVKDNIPNRWFRPETDAPS
SIRSIQIEEQAIKDFKRFYSSVRIVKPQV
>SbANK55
MALKFKASGYIILKEEASCNKDNDSGKDGSLPIRNRYPRQHPIIFFDVKEGCDRKKIDEFLLQWSIPI
AKSRPNDIIIPDPAPQICFRSPRILSLPFLLHVGFHGFIIAAILIWVNDILFDKFDELKRIFIKDSVRRFL
RLFKNCGGRGMSWSLTVTALKPHVVLEGKAPELNGMHANPNCINPYGYFPLHEAAERFSCDMI
KLLLRHGASANVHTVGNDVIENLLPLHVAIENTCMHKYLEDNLSPSQSHLDYIYKLIHLLCLPEM
KIFLDTTRLLAEKTNNLLEELWNYIENGKLIQSAILLLAAQEHIRRGCSSKKNGSSKKDGFYVINKR
ILWLSFALRWEKGSNEMAEKLLNERRALIDCTGLLVDVISQTGQRLSSYIQSHSEVPRVEVLEHVSS
ILKEYGFNPTGEIMDTINLRPYDCKMSDTETCNKGLINPNMAVMESDNLHAAEEKAVTVTKEVG
RGWDPTYMRRSFFPYWRSVLRTRCRVRLYPAYAEGYTRPVLDLEHFRVSRSKSFANGSTPTPNHK
LGLVGRISPLTSNQPRRAFSTAAISAFRLLKMLKYA
>SbANK56
MVCYNALRCAKVVLEGKAPELNGMHANPNCINPYGYFPLHEAAERFSCDMIKLLLRHGASANV
HTVGNDVIENLLPLHVAIENTCMHKYLEDNLSPSQSHLDYIYKLIHLLCLPEMKIFLDTTRLLAEK
TNNLLEELWNYIENGKLIQSAILLLAAQEHIRRGCSSKKNGSSKKDGFYVINKRILWLSFALRWEK
GSNEMAEKLLNERRALIDCTGLLVDVISQTGQRLSSYIQSHSEVPRVEVLEHVSSILKEYGFNPTGEI
MDTINLRPYDCKMSDTETCNKGLINPNMAVMESDNLHAAEEKAVTVTKEVGRGWDPTYMRRS
FFPYWRSVLRTRCRVRLYPAYAEGYTRPVLDLEHFRVSRSKSFANGSTPTPNHKLGLVGRISPLTSN
QPRRAFSTAAISAFRLLKMLKYA
>SbANK57
MASDESKNSKPEKLSSAAGAGLPNPFDFSSMSSLLNDPSIREMAEQIASDPVFNQMAEQLQKSAQ
GAGEQGIPALDPQQYMETMQQVMQNPQFVSMAERLGNALMQDPAMSSMLENFTSPAHKEQL
EERMARIKEDPSLKPILDEIENGGPSAMVKYWNDPEVLQKIGQAMGVGLPGDSSASTALSGPEET
EEEGGDDDESIVHHTASVGDEEGLKKALDGGADKDEEDAEGRRALHFACGYGELKCAEILLEAG
AAVDALDKNKNTPLHYAAGYGRKECVDLLLKYGAAVTLQNLDGKTPIEVARLNSQDEVLKLLE
KDAFL
>SbANK58
MASSPPSSGVLPPSYPLRLREPPLTLRNPRSILPVADLAALLLRAAYDGDVPQLKTLVKRLRKAGKS
VDEAMTEIRASWYKGQGPLHMAALSGKTAVCKLLIKDLKLDVDAAVYDGVTPLSLAILGTASAA
VTRLLLDHHADPNKAAFDGCTPLHLAATRDAYEIAELLLSRRAYVDPVSECGTPLYIAAKNGSA

KMLKLLLRHQADPNRIVCTPLKAISACSLEGMELPTKAHADVNAGTVTPLIAAAYAGSTDCIKCL
LKAGADANIPDQNGRVPIEFAATQRWKECVDVLFPVTTPLAKVADWSTTGIIEHSKLMSSKPQDE
NDGSDFEAQGDAAIGKSDYAHALTLYTMAVEINPDDSSLYAKRSLCSLHAGDKSKALDDANIYK
DMQPELSKSRSEQAAALILVKEYDRAVEVLMTGLSLDFERKPTDKALSEDHP
>SbANK59
MASSPPSSGVLPPSYPLRLREPPLTLRNPRSILPVADLAALLLRAAYDGDVPQLKTLVKRLRKAGKS
VDEAMTEIRASWYKGQGPLHMAALSGKTAVCKLLIKDLKLDVDAAVYDGVTPLSLAILGTASAA
VTRLLLDHHADPNKAAFDGCTPLHLAATRDAYEIAELLLSRRAYVDPVSECGTPLYIAAKNGSA
KMLKLLLRHQADPNRIVCTPLKAISACSLEGMELPTKAHADVNAGTVTPLIAAAYAGSTDCIKCL
LKAGADANIPDQQNGRVPIEFAATQRWKECVDVLFPVTTPLAKVADWSTTGIIEHSKLMSSKPQ
DENDGSDFEAQGDAAIGKSDYAHALTLYTMAVEINPDDSSLYAKRSLCSLHAGDKSKALDDANI
YKDMQPELSKSRSEQAAALILVKEYDRAVEVLMTGLSLDFERKPTDKALSEDHP
>SbANK60
MASSPPSSGVLPPSYPLRLREPPLTLRNPRSILPVADLAALLLRAAYDGDVPQLKTLVKRLRKAGKS
VDEAMTEIRASWYKGQGPLHMAALSGKTAVCKLLIKDLKLDVDAAVYDGVTPLSLAILGTASAA
VTRLLLDHHADPNKAAFDGCTPLHLAATRDAYEIAELLLSRRAYVDPVSECGTPLYIAAKNGSA
KMLKLLLRHQADPNRIVCTPLKAISACSLEGMELPTKAHADVNAGTVTPLIAAAYAGSTDCIKCL
LKAGADANIPDQNGRVPIEFAATQRWKECVDVLFPVTTPLAKVADWSTTGIIEHSKLMSSKPQDE
NDGSDFEAQGDAAIGKSDYAHALTLYTMAVEINPDDSSLYAKRSLCSLHAGDKSKALDDANIYK
DMQPELSKSRSEQAAALILVKVTCATGSMIEQLKFS
>SbANK61
MASSPPSSGVLPPSYPLRLREPPLTLRNPRSILPVADLAALLLRAAYDGDVPQLKTLVKRLRKAGKS
VDEAMTEIRASWYKGQGPLHMAALSGKTAVCKLLIKDLKLDVDAAVYDGVTPLSLAILGTASAA
VTRLLLDHHADPNKAAFDGCTPLHLAATRDAYEIAELLLSRRAYVDPVSECGTPLYIAAKNGSA
KMLKLLLRHQADPNRIVCTPLKAISACSLEGMELPTKAHADVNAGTVTPLIAAAYAGSTDCIKCL
LKAGADANIPDQNGRVPIEFAATQRWKECVDVLFPVTTPLAKVADWSTTGIIEHSKLMSSKPQDE
NDGSDFEAQGDAAIGKSDYAHALTLYTMAVEINPDDSSLYAKRSLCSLHAGDKSKALDDANIYK
DMQPELSKSRSEQAAALILVKEYDRAVEVLMTGLSLDFERKPTDKALR
>SbANK62
MASSPPSSGVLPPSYPLRLREPPLTLRNPRSILPVADLAALLLRAAYDGDVPQLKTLVKRLRKAGKS
VDEAMTEIRASWYKGQGPLHMAALSGKTAVCKLLIKDLKLDVDAAVYDGVTPLSLAILGTASAA
VTRLLLDHHADPNKAAFDGCTPLHLAATRDAYEIAELLLSRRAYVDPVSECGTPLYIAAKNGSA
KMLKLLLRHQADPNRIVCTPLKAISACSLEGMELPTKAHADVNAGTVTPLIAAAYAGSTDCIKCL
LKAGADANIPDQQNGRVPIEFAATQRWKECVDVLFPVTTPLAKVADWSTTGIIEHSKLMSSKPQ
DENDGSDFEAQGDAAIGKSDYAHALTLYTMAVEINPDDSSLYAKRSLCSLHAGDKSKALDDANI
YKDMQPELSKSRSEQAAALILVKVTCATGSMIEQLKFS
>SbANK63
MASSPPSSGVLPPSYPLRLREPPLTLRNPRSILPVADLAALLLRAAYDGDVPQLKTLVKRLRKAGKS
VDEAMTEIRASWYKGQGPLHMAALSGKTAVCKLLIKDLKLDVDAAVYDGVTPLSLAILGTASAA
VTRLLLDHHADPNKAAFDGCTPLHLAATRDAYEIAELLLSRRAYVDPVSECGTPLYIAAKNGSA
KMLKLLLRHQADPNRIVCTPLKAISACSLEGMELPTKAHADVNAGTVTPLIAAAYAGSTDCIKCL
LKAGADANIPDQQNGRVPIEFAATQRWKECVDVLFPVTTPLAKVADWSTTGIIEHSKLMSSKPQ
DENDGSDFEAQGDAAIGKSDYAHALTLYTMAVEINPDDSSLYAKRSLCSLHAGDKSKALDDANI
YKDMQPELSKSRSEQAAALILVKEYDRAVEVLMTGLSLDFERKPTDKALR

>SbANK64
METPPPFQESAHCDVCRCTFGTFRRRHHCRSCGRTLCHEHSSYHMALPQYGIYTDVRVCYDCFN
KSSSQGRVGNSVSAGSISGAADSFSGLSLGKEDASAPMKNSTVQSATPLIECKCGMPLCICEAPKPE
PAPTPVKNISTVPSTTQSNPKPKKPANTQQKASATSSNNSSSFLNIGLMSNDSNDKGLSDYEVSGE
GLREAIKGGDVKGVKKLLTQGVDCNYCDKQGFTLLHLAALFNQTEIALILMDHGANVESKNGQ
GETPLDCAPTMLQYKMRQRMEELAASRRPLE
>SbANK65
MAGGAGGRDPLVASEIHGFLTSADLNFDKLMMEAGTRWFRPNEIYAVLANYARFKVHAQPIDK
PISGTVVLYDRKVVRNFRKDGHNWKKKKDGKTVQEAHEKLKIGNEEKVHVYYARGEDDPNFFR
RCYWLLDKELERIVLVHYRQTSEENALPPPHAEAEVAEVPPINMAHYTSPLTSTDSASAHTELSSV
AAAPEINSNGGRAISSETDDHGSSLESFWADLLESSMKNDTPIGASSCGGSLASNQQTNNGTRDS
GNNILHANATSNAIFAPTTNVVSEAYANPGHNQASENYFGSLKHQANNSPSLLTSDLDSQSKQH
ANSLMKAPVYGNMPNDVPARQNSLGLWKYLDDDISLENNPSSGILPTEQVTDERPFHITDISSEW
AYCTEETKVLVVGYFHENYKHLAGTNLFCVIGDQCVVANIVQTGVYRLIVRPHVPGQVNLYLTL
DGKTPISEVLSFDYRMVPDSQILADDEPQKSKLQMQMRLARLLFTTNKKKMAPKFLVEGTKVSNL
LSVSAEKEWMDLLKFGSDSKGTYVPAIEGLLELVLRNRLQEWLVEKVIEGQKSTDRDDLGQGPIH
LCSFLGYTWAIRLFSLSGFSLDFRDSSGWTALHWAAYYGREKMVAALLSAGANPSLVTDPTHDD
PGGYTAADLAARQGYDGLAAYLAEKGLTAHFEAMSLSKDKRSTSRTQSLKQNTMEFENLSEQEL
CLRESLAAYRNAADAANNIQAALRERTLKLQTKAIQLANPETEAASIVAAMRIQHAFRNYNRKK
MMRAAARIQSHFRTWQIRRNFMNMRRQAIKIQAAYRGHQVRRQYRKVLWSVGVVEKAILRWR
KKRKGLRGIATGMPVEMATDAEAASTAEEDYYQVGRQQAEDRFNRSVVRVQALFRSHRAQQEY
RRMKVAHEEAKVEFSQN
>SbANK66
MAGVDAARYAHSPAHHAVAARDHAALRRVLDALPRARRPEEIRTEADSVAEEARAEAVSAVID
RRDVPGRETPLHLAVRLCDATAVEMLMAAGADWSLQNEQGWSALQEAICAREEALARVIVRH
YQPLAWAKWCRRLPRVVATMRRMRDFYMEITFHFESSVIPFISRIAPSDTYRVWKRGANLRADM
TLAGFDGFKIQRSDQTILFLGEGSEDGKVPPGSLCMINHKDKEIMNALEGAGAPASEAEVQQEVT
AMSQTNIFRPGIDVTQAVLLPQLTWRRQERTEAVGPWKAKVYDMNHVVVSVKSRRVPGAMTDE
EFFSSCNDNDTESEGFDDVLTEEEKKQLEAALKMDSPDAGGESDSFAGPRHSCVEPREREIPIEDM
SVSGNGESKHDKKGWFSSWGKRSQVSKPEGVKKMAPPRSSLCIDEKVSDLLIESPSNVQTRPGRHS
VDVVRADDNRRIRERDNRRHVASAENGRRREGSKESEYKKGLRPVLWLSPNFPLRTEELLPLLDIL
ANKVKAIRRLRDLLTTKLPPGTFPVKVAIPVVPTIRVLVTFTKFEELQPLEEFTTPPSSPDNSKSPAV
QPSSNSWIQWIKAPYRQNFSTAPGPSSRVEDIQDPFAIPSDYVWTTPEEKKKKTQENKSKSKKGRN
GA
>SbANK67
MAEIRKYAMSNQPPDIPQILLEAQNRWLRPTEICQILSNYKKFSIAPEPPNRPQSGSLFLFDRKILRY
FRKDGHNWRKKKDGKTVKEAHEKLKVGSVDVLHCYYAHGEENENFQRRTYWLLEESFMNIVL
VHYLEIKGVKQSFNRAKEAEENAGLSHADSPACSNSFASQSQVASQSMDAESPISGQISEYEDAET
DNSRASSRYHPFTEMQQPVDGIMMGNLFGASAPSVSVNNLAAGYLGEMQPTGANLTNHFSTRN
DVASVFNETGSELGGGPKASIDSVLLGEPFQEYPGGFMDSTLYSSVATLGNSLDDGLQTFMSEALY
TNNLTQKEAENDGYTDQSVRYPLLKQSSSDLFKMEPDGLKKFDSFSRWMNNELPEVADLDIKSSS
DAFWSTTETVNVADGSSIPINEQLDAFVVSPSLSEEQLFSIIDVSPSWAYNGKKTKVLITGTFLAKKE
DVENRRWSCMFGDAEVSAEVLVDGSLRCYTPVHRSGRVPFYVTCSNRVACSEVREFEFRDSETHY
MDTSDQHTTGINEMHLHIRLDKLLSLEQEDYEMYVLSNGNKSELIDTINSLMLDDNLSNLALPFD

EKELSTVRDQNLEKQVKEKLYYWLIHKIHDDGKGPNVLGKEGQGAIHLVAALGYDWAIKPIVA
AGVNINFRDIRGWTALHWAASCGRERTVGALIANGAASGPLTDPTQQYPSGRTPADLASENGH
KGIAGFLAESALTSHLSALTLKESQGGNVEEICGVTAPAAEDFAEPSSSQLACVNSQEESLKDSLG
AVRKSTQAAARIFQAFRVESFHRKKVIEYGDDDCGLSDERTLSLISLRNPKPGHGDLHSAAVRIQN
KFRGWKGRKEFMIIRQKIVKIQAHVRGHQVRKNYRKVVWSVGIVEKVILRWRRKRRGLRGFQPE
KQLEGPSWQIQPAKAEAEDEYDFLKDGRKQAEGRLQRALARVHSMNQYPEARDQYRRLQACV
NSLQESQAMQDRMLADSAGTDGGDFMAELEELCRDDGDAPMSTIS
>SbANK68
MAEIRKYAMSNQPPDIPQILLEAQNRWLRPTEICQILSNYKKFSIAPEPPNRPQSGSLFLFDRKILRY
FRKDGHNWRKKKDGKTVKEAHEKLKVGSVDVLHCYYAHGEENENFQRRTYWLLEESFMNIVL
VHYLEIKGVKQSFNRAKEAEENAGLSHADSPACSNSFASQSQVASQSMDAESPISGQISEYEDAET
DNSRASSRYHPFTEMQQPVDGIMMGNLFGASAPSVSVNNLGYLGEMQPTGANLTNHFSTRNDV
ASVFNETGSELGGGPKASIDSVLLGEPFQEYPGGFMDSTLYSSVATLGNSLDDGLQTFMSEALYTN
NLTQKEVDALSAAGITSSKAENDGYTDQSVRYPLLKQSSSDLFKMEPDGLKKFDSFSRWMNNELP
EVADLDIKSSSDAFWSTTETVNVADGSSIPINEQLDAFVVSPSLSEEQLFSIIDVSPSWAYNGKKTKV
LITGTFLAKKEDVENRRWSCMFGDAEVSAEVLVDGSLRCYTPVHRSGRVPFYVTCSNRVACSEVR
EFEFRDSETHYMDTSDQHTTGINEMHLHIRLDKLLSLEQEDYEMYVLSNGNKSELIDTINSLMLDD
NLSNLALPFDEKELSTVRDQNLEKQVKEKLYYWLIHKIHDDGKGPNVLGKEGQGAIHLVAALGY
DWAIKPIVAAGVNINFRDIRGWTALHWAASCGRERTVGALIANGAASGPLTDPTQQYPSGRTPA
DLASENGHKGIAGFLAESALTSHLSALTLKESQGGNVEEICGVTAPAAEDFAEPSSSQLACVNSQE
ESLKDSLGAVRKSTQAAARIFQAFRVESFHRKKVIEYGDDDCGLSDERTLSLISLRNPKPGHGDLH
SAAVRIQNKFRGWKGRKEFMIIRQKIVKIQAHVRGHQVRKNYRKVVWSVGIVEKVILRWRRKRR
GLRGFQPEKQLEGPSWQIQPAKAEAEDEYDFLKDGRKQAEGRLQRALARVHSMNQYPEARDQY
RRLQACVNSLQESQAMQDRMLADSAGTDGGDFMAELEELCRDDGDAPMSTIS
>SbANK69
MAEIRKYAMSNQPPDIPQILLEAQNRWLRPTEICQILSNYKKFSIAPEPPNRPQSGSLFLFDRKILRY
FRKDGHNWRKKKDGKTVKEAHEKLKVGSVDVLHCYYAHGEENENFQRRTYWLLEESFMNIVL
VHYLEIKGVKQSFNRAKEAEENAGLSHADSPACSNSFASQSQVASQSMDAESPISGQISEYEDAET
DNSRASSRYHPFTEMQQPVDGIMMGNLFGASAPSVSVNNLAAGYLGEMQPTGANLTNHFSTRN
DVASVFNETGSELGGGPKASIDSVLLGEPFQEYPGGFMDSTLYSSVATLGNSLDDGLQTFMSEALY
TNNLTQKEVDALSAAGITSSKAENDGYTDQSVRYPLLKQSSSDLFKMEPDGLKKFDSFSRWMNN
ELPEVADLDIKSSSDAFWSTTETVNVADGSSIPINEQLDAFVVSPSLSEEQLFSIIDVSPSWAYNGKK
TKVLITGTFLAKKEDVENRRWSCMFGDAEVSAEVLVDGSLRCYTPVHRSGRVPFYVTCSNRVACS
EVREFEFRDSETHYMDTSDQHTTGINEMHLHIRLDKLLSLEQEDYEMYVLSNGNKSELIDTINSLM
LDDNLSNLALPFDEKELSTVRDQNLEKQVKEKLYYWLIHKIHDDGKGPNVLGKEGQGAIHLVA
ALGYDWAIKPIVAAGVNINFRDIRGWTALHWAASCGRERTVGALIANGAASGPLTDPTQQYPSG
RTPADLASENGHKGIAGFLAESALTSHLSALTLKESQGGNVEEICGVTAPAAEDFAEPSSSQLACV
NSQEESLKDSLGAVRKSTQAAARIFQAFRVESFHRKKVIEYGDDDCGLSDERTLSLISLRNPKPGH
GDLHSAAVRIQNKFRGWKGRKEFMIIRQKIVKIQAHVRGHQVRKNYRKVVWSVGIVEKVILRWR
RKRRGLRGFQPEKQLEGPSWQIQPAKAEAEDEYDFLKDGRKQAEGRLQRALARVHSMNQYPEA
RDQYRRLQACVNSLQESQAMQDRMLADSAGTDGGDFMAELEELCRDDGDAPMSTIS
>SbANK70
MQPTPADVEQGIVDAVPRRHLKGVTMEGDTALHLVAANGDDSSFQKCAVVIHGKDKDLLCRQ
NNKGDTPLHCAARTAGRSEMVSHLIVLATVDNIVEQLLRQENNSNETVLHMAVRTGDHQLVK

HLLAKDPKLACFPEKGTSPLYLAILLDQGSIAKMLYDESENNVLSYAGPNGQNALHAAVLRGPD
VQACRTEDGVEAHVHANTAALYQPDNNGLYPIHVAAVVVGASVWLMRFERHPIPMFVKKCPS
SAGLRDAKGRTFLHVAVEKKNVDVVWYACRHPSLAWVLNMQDGEGNTALHLAVRDGNTLGI
FRHLFGSMQVNLNLTNAKKQTPRDIALYHLRPSFYFETANPEIWIKRALQIAGATRGVYRKDHFD
EEYENHHGLNSDYNKDKELEMLKDSTQSRSIGSVLIATVTFGAMFALPGGYKADDHSFGGTPTPA
GMYAFHAFMIANTIAFISSTIATLGFMFAGDAGISLARRKLHFSGAMVSTQYSITALTIAFALGVYT
VLAPVAQKTAILICVISPLVVLYNISDFWCSAAYGRNNTISISISPAQAPARFT
>SbANK71
MGLQQSKEELLYQQVNYGNVDGIRTLRGQGVGLEWIDKEGKTPLMVACMRPDLFDVAKVLIEL
GANVNAYRPGSHCGTALHHAAKKGLQQTVHLLLSHGANPFIPNDDCNTALELAREKGHVNVV
RAIEGRLSLFCGWMRENYAPAFLDAIAPQFMTRKIWAVILPREVRTPTRPLKLELAIYPELQASKPR
VVVKLWKCQIEQPKLNQADPSIIIFDKGTKTRYKILPAYEGDKQQLQWFYNACCGMAQVFNTAP
VPPANLPMPNPAPANSSVAPSELSAPSKEDVELAMAINASIQSAIAEGVPNVQPNASTPNNNGW
SIPPSNSHNGWGPSVTPAPSKTSGQSQARVDAPSSSSTYNGWDVPGTSSGQSSSKPHKTETNPPVLI
PQEALQALPTPTAPPFAEETFYSGPVHYPSIDSTPVDVTMPATTEGGTTGSTTAAAPTKQEENEAD
ASDSGKTPSGTCVICLDAPVEGACIPCGHMAGCMSCLKDIESKKWGCPICRATINQVVRLYAV
>SbANK72
MESSLSYSGGSSSFPQQVQARNPSANLSVEDITVKLLSAAYAGDVPQVKKLAKRLAKAGKSMDE
AVAEIKAPWNRGHGPLHLAAAAGKVETCKFLIKDLKVHVDTTGSIGVTPLILAIQHGCSAVIRFL
LVHDADPSKAASNGVTPLHTAAAQDACELAELLLSKGAYVDPMWEKRTPLYIACQRGNARMM
EILLQHQADPNAAVLLVHTPLKAAVFARSLEGVELLIKAGADVNAGQPVTPLIGAATAGFTDCV
KCLLKADADANIPDNNGRVPIEIAAIKGWQECVEILLPVTTPLARVADWSIEGITQHAKLSRPNL
QDPLLHENDKPDFEANGDAAFSDRDYALALNLYTKAVETDPGNSTLYAKRSLCSLHTGDKSNA
MYDADTYKGMEPDLSKSCYAQGAALILVKEYDRACEVLMSGLHLDFESKLTDTASSGDHQ
>SbANK73
MASPEAAETSPPSTPTTSASPCPTPRPLATAPPTMGATLLRAARSGDERRLVKALLADPAAPDLET
AATAGGNTLLHVAAVGGHADLASLLLRRAPRLLAARNAALDTPLHLAARAGAHKVVALLVAS
SSSSSPACSLRGLTRATNRRGETALHDAVRGGHEAAARALAAADPGLVELCGGAGESPFYMAAA
AGSLGMVRLLLKTYRDADEEEEVAGLGSSMGPGGRTVMHAAVLTSNEMIRELLQWNPTLVKEV
DDSGSTPFHYIASVGNISAMKLLLRRDSSVAYSSDSNGLFPVHIAAKMGYGQFIYELCRFCPDCDE
LLDNRGRNFLHIAIEHKKWKVVWCFSGTEDLGRMANVMDSEGNTPLHLAVKNADQMIVSLLM
ATKGVLPNIVNNQGLTALDLAVLATDKGISYTLNPQVIILRCLAWTGAVLSPRRLDHFIDEFGIGK
TSGNELKKFTNIAQNLIVGSVLVSTVTFAAVFTLPGGYISDGHPHAGAPILSHRYTFKAFVMANTL
AFVGSTLSTIWLTYAGSEHVHPLLRAIYMFLSVISMEQATRSMVIGFALGAYVVLSPVSERIAIVVC
MSTFMTLLLRNPSSWQLWFLFMPIKRRLGWKGAFKTHLPLETRSRLTIGVGSNFACQFLRRMLGM
LFTYSFIFLLALL
>SbANK74
MASPEAAETSPPSTPTTSASPCPTPRPLATAPPTMGATLLRAARSGDERRLVKALLADPAAPDLET
AATAGGNTLLHVAAVGGHADLASLLLRRAPRLLAARNAALDTPLHLAARAGAHKVVALLVAS
SSSSSPACSLRGLTRATNRRGETALHDAVRGGHEAAARALAAADPGLVELCGGAGESPFYMAAA
AGSLGMVRLLLKTYRDADEEEEVAGLGSSMGPGGRTVMHAAVLTSNEMIRELLQWNPTLVKEV
DDSGSTPFHYIASVGNISAMKLLLRRDSSVAYSSDSNGLFPVHIAAKMGYGQFIYELCRFCPDCDE
LLDNRGRNFLHIAIEHKKWKVVWCFSGTEDLGRMANVMDSEGNTPLHLAVKNADQMIVSLLM
ATKGVLPNIVNNQGLTALDLAVLATDKGISYTLNPQVIILRCLAWTGAVLSPRRLDHFIDEFGIGK

TSGNELKKFTNIAQNLIVGSVLVSTVTFAAVFTLPGGYISDGHPHAGAPILSHRQREVW
>SbANK75
MDKQSSTRHGTLEKLKSFRGIEKQRSFKFLSMEKQQSFKRNKDSPGKRGDTELHLAARAGSVAH
VQRILAECDPELVVELAARQNQDGETALYVSAEKGHVEVVCEILKASDVQSAGLKASNSFDAFHI
AAKQGHLDVLKELLQAFPSLAMTTNSVNATALDTAATQGHIDIVNLLLETDASLARIARNNGKT
VLHSAARMGHVEVVTALLNKDPGIGFRTDKKGQTALHMASKGQNAEILLELLKPDVSVIHVED
GKGNRPLHVATRKGNTIMVQTLISVEGIEINAVNRAGETAFAIAEKQGNEELINILREVGGETAKE
QVNPPNPAKQLKQTVSDIRHDVQSQIKQTRQTKMQFQKIKKRIQKLHIGGLNNAINSNTVVAVLI
ATVAFAAIFQLPGNFLEDITQAPYPDMTLGQALIASDPAFIIFLVFDALALFISLAVVVVQTSLIVVE
QKAKKKMVFVINKLMWLACLCISAAFIALTYVVVGRDDEWLAWCTMAIGTVIMVATLGSMCYC
IVAHRMEEKSMRKIRRTSTSQSFSISIDSETELMNSEYKKMYAL
>SbANK76
MPLCRGGGGAAGGGSSSGGLAAGRTSNRYSTSIGALLPARGGGWRPRLRLRRFIVSPYDPRYLAW
EHSLVVLVAYCAWVAPFEFGFVPDPHGALAIADNAVNAAFAVDIALTFFVAYADGRTYLLQDD
PRRIAWRYASTWLALDVASTVPIDVYRRILPPQASGYNFFGMLRLWRLHRVGTFFTRLEKNRKLS
YFWVRCIRLICVTLFAVHSAGCFYYLLADRYHDADHTWLSSSMPDFKQESVWKRYAASMYWSIT
TLTTVGYGDMHAVNTGEMVFTTLYMLFNLGLTAYLIGNMTNLVVHGTSRTRKYRDAIKAATSF
AVRHQLPARLQEQMVSHLSLKFRTDSEGLQQQETLDALPKAIRSGISHHLFFALVQGVYLFQGVS
NDLIFQLVSEMNAEYFAPREDVILQNEAPSDFYVLVTGSVELIELQNGSEQLAGMARAGDVVGEI
GVLCYKPQLFTARTRTLCQLLRMERTAFLRIVQANVGDGTIIINNLIQYLKEKRDSCAIAGVAEEIE
YMLARGQLELPVTLCYAASKGDDFLMRQLLKRGVDPNESDNYWHTALHISASSGHDQCVKLLL
EHGADPNATDAQGRVPLWEALSRRHRAAARLLVDAGADLSAGDAALYARAAVEADDVALLE
DVARHGGDVTVACWDDGVTALHRAVLQGNVGMARVLLEHGADADREDGGGRTPRALADQL
GHRDMQQLFIGSSSSSSHHREGAVESPKQQQGSGTDHGGSRLAVVAAHQPAVTRFKSAPAPRVP
VVPQHDSAGSSPSPSPSPPQSSRHSTPRRLVGFRNSLFGVLSSSHLHWHDGGGGAGGGGGPSRHE
RQTQSSRSRVRVTISCPEQGASARKLVFMPETMVQLVELAGSQFGFAPTRAVTMDGAQVDDARL
VRDGDHLLLVTHQWVPDTKVVQTNQ
>SbANK77
MFFSRLDDSPMFRKQMQSLEEGADLLRERCLKFHKGCRKYTEGLGEAYDGDIAFASSLETFGGGH
NDPISVAFGGPVMTKFTIALREIGTYKEVLRSQVEHMLNDKLLQFVEIDLHEMKDARKRFDKATL
LYDQAREKYLSLKKGTRTDVAAAVEDELHSARSSFEQARFNLVTALSNIEAKKRFEFLEAVSGTM
DAHLRYFKQGYELLHQMEPYINQVLAFAQQSRERSNYEQAALLERMQDFKRQIDRESRWSPNG
MNDSPNGDGIQTIGRSSHKMIEEAMQSASKGKVQTIRQGYLSKRSSNLRGDWKRRFFVLDSRGM
LYYYRKQNSRPSSGYNQRTSIPSEHGSGLLSRWFSSHYHGGVHDEKSVARHTVNLLTSTIKVDAD
QSDLRFCFRIISPTKNYTLQAESAMDQMDWIEKITGVIASLLSSQSPERRLLLSPKGSSHHRTASSSSS
FSSSTELEHSINEDCMLEKNSGSGYFEHSARVAQHHRTSMMKPDKPIDLLRKVAGNNSCADCGA
SEPDWASLNLGVLLCIECSGVHRNMGVHISKVRSLTLDVRVWEPSVINLFQSIGNTFANTVWEEM
LPSSTCVDHGDISRPDGLENMSHRFAPSKPKQSDSIAVKEKFIHAKYAEKDFVRKHNVDEIQLAE
QMWDNVSSNNKKGVYSLIVGSNADVNFSYGHTSFNSALTLGKALLLQEQSTSPSNGSSRCFDLNP
LEKGSTGDSVSPASTSARIDGLDDYVEGLSLLHLACRVADLGMVELLLQYGANVNSTDSRGRTPL
HHSIMKGRHVYAKLLLSRGADSQAVDRDGRTALKYAIDSGTIEDEEILVLLEDPSR
>SbANK78
MAGHREVAACLLSAMRAGGASADALRARNGLGATALYEAVRNGHAETVVLLATEAPELAAM
TTDGGVSPLYLAAMTGSVEMVRALLRPAPDGTPSLASFAGPEGRTALPAAASKSKEIVQEILESEQ

GLALLPRADLSGKTPLHYALLSHRQHGVVSLLLSAEASLARVSDNEDRRRRNFLHCAIEHNQEG
VVRFICRDGMFAILLNAMDYEGNTPLHLAVKYGHPRMVSFLLQTMSVEVGITNVDGLTPADLAY
SHLEPGLHYFLNPRAVVKNCLYWTRAPVTGEDHVRLHSRMSTTTTPAMDEDPKDIDGITATATI
ASVLIATVTFAAAFTVPGGYVADDHPRAGTAVLARRLAFRAFVASDTMAFLCSIVATCFLVYGG
AGQVPRGQRRLYQ
>SbANK79
MAGHREVAACLLSAMRAGGASADALRARNGLGATALYEAVRNGHAETVVLLATEAPELAAM
TTDGGVSPLYLAAMTGSVEMVRALLRPAPDGTPSLASFAGPEGRTALPAAASKSKEIVQEILESEQ
GLALLPRADLSGKTPLHYALLSHRQHGVVSLLLSAEASLARVSDNEEFVDRRRRNFLHCAIEHNQ
EGVVRFICRDGMFAILLNAMDYEGNTPLHLAVKYGHPRMVSFLLQTMSVEVGITNVDGLTPADL
AYSHLEPGLHYFLNPRAVVKNCLYWTRAPVTGEDHVRLHSRMSTTTTPAMDEDPKDIDGITATA
TIASVLIATVTFAAAFTVPGGYVADDHPRAGTAVLARRLAFRAFVASDTMAFLCSIVATCFLVYG
GAGQVPRGQRRLYQ
>SbANK80
MAFLCSIVTLGTDLFRVLTTGEVPRLVELLNASGEGGPQAADGHVAIKVNGGAVSPGAGTGSLLG
VTSNGNTALHLVASRGHAELTAFVCDSAPSLGVVACLLSKMRTAVGGPDEAAALRARNCLGAT
ALLEAVRLSRAGVVDLLMAEAPELASVTTEDGVSPLYLAAEVWSDEMVRLLLRPSPDGTPSPASF
AGRDGQTALHSAATISKGMAREILNWEPVGPTLLTRVDSSGKTPLQFAILYRRLDVVEMFLDDHT
SSEQAHISDNHGLFPVHSAAKVGSTRIIDVLIQKCPDYCELLDDKGRSLLHCAVEHNQESVVRHV
CQNGTFVTLLNAMDSEGNTPLHLAVKYGCPRIVSLLLQTMSVKTGITNKDGLTAGDLAHRVLER
GRMNYFLLAVIH
>SbANK81
MEQILDVEFAERREEALRKAKDLGDKNLQAALCCARTLPDDALRLPLLQLPDDPEYFHGEAAQI
VERKFMCSELYRAALSGSAQQVQDLVAPSVPTTGTTHQGGCSLDEVTAGRNTVLHVAAGRGLV
ALVQQLFVFVGHEAAAALLPYVNSRSETALHRAARAGRPKMVALLIRLAQEHGPGAAVLLGRK
NSAGDTALHVAARHGREAVVQVLMVAAPALSSTVNDAGLSPLYLAVMSRSVDAVKALVQWR
HASASGYKGQNALHAAVLHSADGITPWQSKAIKEWHEKTSKNLGIVAVLIATVALSAMFNVPG
GYDSGGAANLRSKQVTYNAFLVLDTVAVAASVISTMLLTYGRGAARSSAAWICLALIFLWVALM
SMVLAFMAAVVSGLDSTTTKGIIWSIFALPFAFLVALSFVWAVPAPTFTSLCLVLRALTGEDRRRM
RRHIGRRFPLVGFYLLVMYLFWFLNAVAFCLTVYVIVTTI
>SbANK82
MPVQDPSSSPSAAGGGGGARAKRTSSAPIRPADYAHSPTHHFVALRDAAGLQAILAGLPPLAHPS
RVLTAADAAREARLASSVAAALDRRDVPGGDTALHLAVRLRLPSLASALAAAGADPTLQNHAG
WTPLQEALCLGCREIAACLLRAHRLAAWAKLRRRAPALSTALRRVQDFYLEVDFHFESSVVPLLS
RAAPSDTYRIWKRGADLRADTTLAGFDGLRIRRADHSFLFFGEETSAGGRRLPPGSLLVLHRGRRE
VHDAFAAAAAAGDEDAATSDAAAYRPGLNISSARLVPRTTWLRKEKTENVGEWKARVFDVHN
VVFSFRTLKAASAGRKDFTFELAGDEDGDEDDDEFLPLEIRDDDEDGDFLVADIPPPPARRSCYVP
GRRSVAGPPSHMGTPQRRRNSVDVPRRLPACASVGRGEDGIFGRHPGTTTTGGAKWKEEETVKT
LRPSVWLTEDFPLSVDEFLPLLDILASRVRAVRRLRELLTTKFPPGTFPVKVAIPVVPTVRVVITFTKF
VPLVEPEEFFTPMSSPSLLASPGPGSIMAKPDTHKSSYLRWGSKNSRPKAVNLSQVADNADPFTVP
SDYTWVNSLGSKNHDKKSSKSKKGKTKET
>SbANK83
MASSSGRPSPVQPRVRPSPLELGFGNPSASHNPSIEDRVALLLRASHDGYVPKIKTLAKRLKEHAG
MSVDEAVRWVQAPWSKRHGPLHMAAAAGKFKACKYLIKDLGLDVNATGTDGATPLVFAIHGS

GSLAVVRLLLDHDANPNRADIYGSYPLHIAAIRGYYEISELLLSKGAYADPQWKSKSPLYIAAQCG
NARMVELLLHHDAEPKHVVYTPLKAAISGRSLIGLELLIKAGAPVNIGLPETPLVEAAAAGLTDF
VKCLLKAGANANIPDDNGRVAVEIAAIQGWQECVEVLFPVTAPLARVADWSIDGLLQYARSISS
KTQHPVLHEDHEPDYEAEGDATFCRRDYSHASNLYTMAMEIDPDNSTLYAKRSLCFLYTGYEGE
ALEDATTYKDMHPNLSKSCYEQGAALILVKEYGRTCEALLPGLISDF
>SbANK84
MGDLADLGGAGESARSHVAAAGGGGQRDRLAALLELAAADDAVGFREALAVGGAQGNLAEL
ADGVGLWYGRSKAYEPRTPLMVAATYGSVEVVSLLLGLGCVDVNRRPGVDGATPLHCAASGGS
RNAVAVVKLLLAAGADPVTPDSAGRLPADVILSPPASPDALGDLEMLLGRRRGLAVATSVPSRSS
SPPLSSSPDEGNRSPSSRSSSLSPITVDRAKKEYPVDPTLPDIKSSVYASDEFRMFAFKVRPCSRAYSH
DWTECPFVHPGENARRRDPRKHPYTAVPCPNFRRPGGCPSGDSCEFSHGVFESWLHPSQYRTRL
CKEGAACARRICFFAHDEDELRHVPHNNGAGLLSPRASSSIDMTAAAALGLLPGSPTRHFVPPPL
SPSAANNGGGAAAAHWLQGSRLRSSFNARDAQVDDLGALLEWESQYLGALSLPQSSRSQPRLST
GLSIRPTGIAPSNLEEMYASDMAMSPRFTNDQGHSVYSPAHKSALLNKFHHQQKGLLSPVNTNR
MYSPRGLDPSILHYPYGGMSPRSPRTMEPTSPLSVCVGATVTQRDMLDQFSSLNKHQVPSVGSPR
NLNASWGNIGTPKSKVDWGVDDDELVRLRHPVQPGNTAEEPDVSWVQSLVNHAELNGKRGE
MAGMASRSINRPDLSSQGDSLDQSVIASWLEQQMHLEPK
>SbANK85
MASIPCTFQLSTAVSRRVSPRAAAQGKGQGVAGLRTPLVGAGRRGLGWLRPSRLSRVVPASESGR
VGPTCWFKFGNKDAEGAGIYGSQARDDFDRDDVEQYFNYMGMLAVEGTYDKMEALLNQKIHP
VDILLMLAASEGDRPKIEELLRAGAKFDVKDVDGRTALDRATDEIREFILGFAAKKV
>SbANK86
MSASEEDAAPAAADGGSGGGSSGAGSSEGGARRRFDDKGLVARTSLILWHTHQNDVGAVRKLL
EEDAALVNARDYDSRTPLHVAALHGWHDVAECLIANGADVNAQDRWQNTPLADAEGAKRQS
MIELLKEHGGLTYGKTGSHFEPKTIPPPLTNKADWEINPLELDFTKALVIGKGSFGEILKANWRGT
PIAVKRILPSLSDDRLVIQDFKHEVNLLIKLRHPNIVQFLGAVTETKPLMLITEFLRGGDLHQYLKE
KGALNPLTAVSFALDIARGMAYLHNEPNVVIHRDLKPRNILLVNSAANHLKVGDFGLSKIIRAQ
HANDVYKMTGETGSYRYMAPEVFKHRKYDKKVDIFSFAMILYEMMEGDPPFSSYEPYEAAKYVA
DGHRPIFRKSHTNELKDLVELCWSGDISLRPSFLEILKRLEKLKEHYSHENHWHLFQ
>SbANK87
MGVEPAQPNPTAAAVTATTAEQAQDLIDAARYDDLEDVVAMFSAGISLDSTDSQGRTALHMAS
ANGHLAVVEYLIQNGANVNATNLEKNTPLHWACLNGHIEVIKALISAGASVSALNSHEKTPMD
EAVSKGKMDVIDAIGAAVAQAELDGVTVS
>SbANK88
MKKNSSFQSTGSAGGGSGTGTGSRSGSFNLRNLSKVILPPLGGPSAGQSPNNGGSDKWVISPLDSR
YRWWDTLMVVMVAYSAWVYLFEVAFMNASPKGGLEVADIVVDLFFAVDIVLTFFVAYIDPRSQL
LVCDRKKITFRYLSTFFIMDVASSIPFQAFAYFITGEVREDAVYSVLGLLRLWRLRRVNQFFTRLEK
DIRFSYFWIRSARLVAVTLFVVHCAGCLYYLIADRYPHSEKTWIGAVIPNFRQASLGIRYITAIYWSI
TTMTTVGYGDLCARNTIEMIFNIFYMFFNLGLTSYLIGNMTNLVVEGTGRTMEFRSSIRAASSFVG
RNHLPPRLKQQILAHMCLKFRVENLNQQQLLDELPKPIYKSICEHLFVPVVKDVYLFNGVSREML
LSLVTKMRPEYIPPKEEVIVQNEAPDDVYVVVSGEVEVILFDGIDERVEATLGTRDIFGEVSALSDR
AQAFTFRTRTLSQLLRLKQATLKEAMQNRPEDSVVVIKNFLKHHVEMHGINVEDLLIDNIGEHD
DDANVLTVAAMGNSGLLEDLLRAGKDADVGDAKGRTALHIAASNGYEDCVLVLLKHACNVNI
KDAQGNTAMWNAIAAGHHKIFNILYQFAHASNPHAGGDVLCLAARRGDLDALRELLKLGLDV

DSEDHDGATALRVAMANGHVDVARFLIMNGASVDKANLDDDGSSAARLTMSPTELRELLQKR
ELGHSITIVDSSPAVVPDGGSSGHSRPGRLQSTSSDNQRSPRVSLYKGHPSLRNRSSEAGKLINLRE
ATMGEFKAIIGEKLKVDAEKALIVNDEGAEIDSIDVIRDNDKLFVVTQEDLRRLATMNLSSS
>SbANK89
MTKKSIKNEKGKQNNCFKQFDAASFAVMCCARRYLSTFFIMDVASSIPFQAFAYFITGEVREDAV
YSVLGLLRLWRLRRVNQFFTRLEKDIRFSYFWIRSARLVAVTLFVVHCAGCLYYLIADRYPHSEKT
WIGAVIPNFRQASLGIRYITAIYWSITTMTTVGYGDLCARNTIEMIFNIFYMFFNLGLTSYLIGNMT
NLVVEGTGRTMEFRSSIRAASSFVGRNHLPPRLKQQILAHMCLKFRVENLNQQQLLDELPKPIYKS
ICEHLFVPVVKDVYLFNGVSREMLLSLVTKMRPEYIPPKEEVIVQNEAPDDVYVVVSGEVEVILFD
GIDERVEATLGTRDIFGEVSALSDRAQAFTFRTRTLSQLLRLKQATLKEAMQNRPEDSVVVIKNFL
KHHVEMHGINVEDLLIDNIGEHDDDANVLTVAAMGNSGLLEDLLRAGKDADVGDAKGRTAL
HIAASNGYEDCVLVLLKHACNVNIKDAQGNTAMWNAIAAGHHKIFNILYQFAHASNPHAGGD
VLCLAARRGDLDALRELLKLGLDVDSEDHDGATALRVAMANGHVDVARFLIMNGASVDKANL
DDDGSSAARLTMSPTELRELLQKRELGHSITIVDSSPAVVPDGGSSGHSRPGRLQSTSSDNQRSPRV
SLYKGHPSLRNRSSEAGKLINLREATMGEFKAIIGEKLKVDAEKALIVNDEGAEIDSIDVIRDNDKL
FVVTQEDLRRLATMNLSSS
>SbANK90
MTTVGYGDLCARNTIEMIFNIFYMFFNLGLTSYLIGNMTNLVVEGTGRTMEFRSSIRAASSFVGRN
HLPPRLKQQILAHMCLKFRVENLNQQQLLDELPKPIYKSICEHLFVPVVKDVYLFNGVSREMLLSL
VTKMRPEYIPPKEEVIVQNEAPDDVYVVVSGEVEVILFDGIDERVEATLGTRDIFGEVSALSDRAQA
FTFRTRTLSQLLRLKQATLKEAMQNRPEDSVVVIKNFLKHHVEMHGINVEDLLIDNIGEHDDDA
NVLTVAAMGNSGLLEDLLRAGKDADVGDAKGRTALHIAASNGYEDCVLVLLKHACNVNIKDA
QGNTAMWNAIAAGHHKIFNILYQFAHASNPHAGGDVLCLAARRGDLDALRELLKLGLDVDSE
DHDGATALRVAMANGHVDVARFLIMNGASVDKANLDDDGSSAARLTMSPTELRELLQKRELG
HSITIVDSSPAVVPDGGSSGHSRPGRLQSTSSDNQRSPRVSLYKGHPSLRNRSSEAGKLINLREATM
GEFKAIIGEKLKVDAEKALIVNDEGAEIDSIDVIRDNDKLFVVTQEDLRRLATMNLSSS
>SbANK91
MLEISASSTPLGKKEAALAACHGDNGKGGEGAIADDMDLSRPFLVVKNLDYKKILEWSRMRSAF
VAKSIPKDIIIPDPTPKWVCDAFSKIVPRLGLILEKDNVQCFLRLFESEEGMHWDLSITSQTLTYIVIH
NALRCARVVLVGKAPKLHGMHANPNCMNPYGYFPLHEAAERFSVDMIKLLFRHGAIANVRTV
GNKVIENLLPLQVAVENTCLHKYLEDNLSPIQDHQDYAYNLIHLLCLPEMKIFLDTVRLLAAETD
NLVDEIWSYIKDEKVVQTAVLLLAAQGQIRGNHPSKRNISGKQNGFEVIMGRMVELYQNSHKQL
VESIALKCTYLLVRIISHTGEVLDPYIKDHSEVPHAEVLEHVSSILKDSGFCPTGKGIDVRNLRPYDC
KISGELHAKDATKTVTKTSHAAGKKVLRKKRASGWDPDYTRRLFFPYWRSILGARCLVKVYPSH
APAPASELDPEHFNSWSGSLGNGSSQSPNHNLELLGRTQLPTSNHPQRRPFGTAAFRGTTAATTT
TARN
>SbANK92
MCSGPRKPSTPPLPTATAKDSSAMTAAAAVLLELAAADDVVAFRRAAEDDKAPALDAVAHWY
GPSAGAGRLRLEARTPAMVAALYGSASVLAYVLSSAPAEAARASPTDGATPLHLAAAGGAAGA
VAATHLLLAAGASADALAFSGLRAGDLLPRANAAADRDRALRVLLKSPAASPSSSPKKSASPPPP
LAAAAAAAAAALPAEPRKEYPPDLTLPDLKSGLFSTDEFRMYSFKVKPCSRAYSHDWTECPFVHP
GENARRRDPRRYSYSCVPCPEFRKGGACRKGDNCEYAHGVFECWLHPAQYRTRLCKDEVGCAR
RICFFAHKPEELRAVNPSAVSVGMQPTVSSPRSSPPNGLDMGGGMLNPAWPSSPASRLKTALAGR
ELDFDLELLALDQYQQKLFDKVSSPRASWGSAGGIGSPLPAASPARTVPDYTDLLGSVDPAMLSQ

LHALSLKQAGDMPAYSSMADTQLHMPTSPMVGGPNTAFGLDHSAMAKAIMSSRASAFAKRSQS
FIDRGGRAPATRSLMSQQSTTGAPSMLSDWGSPDGKLDWGVQGDELHKFRKSASFAFRGQSPAP
VPTPAEPDVSWVNSLVKDGHAGDIFAQWPEQEQMVA
>SbANK93
MPCSNHTMVMMSPSAGVPPRHTPHTMNHELLRAAATGDKALLEQVLGLSSTTDNGGELEATH
RGSRSCLKGVTSEGNTALHIAAGRGYLEHARIMCDLDESLVKARNNMRNTPLICAARAGHVDV
VCYLIGHALAAPATMAAAPAPAWDSGASSGEESMLRARNSEGATAMHEAIRNGHEPVLAKLM
AADGGLAAVVDGMGFSPLYLAAALGRADMVDVLIAGSPPDGVKSPAYYAGPDGQTALHAAVL
ASEEMSKSLWCWEPTLAKKVDNSGNTALHHAASAGKIGAVKLLLLEDSSLAYIPDVDGLFPVHT
AAKMGKVGIIEQLMETCPNSDELLDNRGRNVLHCAIEHKKEKVVQHMCKNPRFGRMTNARDS
RGNTPLHLAVKHGCDRIAMLLMRDVKVNLSIMNNDGATPLDLAINELDHGYTNPMNPEVLIAQ
CLVWCGAHRSPRRRDECLNKRTGVGCSEKELSKYTNLTQNRAIGSVLIATVTFAAPFTMPGTAA
DAAERPAFWAFVLSDALAFMCSTVATCLLMYAGLTTVHPRHRSRYHVWSSNLLHVGILLVIATF
AVGVHLALSPPGTGIPGGNLNAAVCAMACVSVVFAHPGTWWPMVLAKPIWARLGLKGLLGVL
LGPRPIPCQKLLLSRTPWLNLFKMVATLLILALILVTFLLDMSYLRPPAAQHRFSPNLLVQPQLQFC
SYPT
>SbANK94
MGHGVSCARTGDEHDYFRAAQVGDLDALGALLAADPSLARRATLYDRLSALHIAAANGRLEVL
SMVLDHGVPPDAVNRHKQTPLMLAAMHGKIDCVLKLLQAGANILMFDSLHGRSCLHHASYFG
NVDCLQAILTAARTTPVADSWGFARFVNVRDDHGATPLHLAARQGRPGCVQVLLENGAIVSAL
TGSYGFPGSTSLHLAARSGNLDCIRKLLAWGADRLQRDSAGRIPYAVALKRNHEACAALLNPSS
AEPMVWPSPLKFISELDPEAKALLEAALMEANREREKKILKGTKYSPPSSPPSTCEHEIDADAIDDV
SSETSDTELCCICFDQACTIEVQDCGHQMCAPCTLALCCHSKPNPTTLTLPSPACPFCRGNISRLLV
ARASTASLDADTDKAAAAAASSPQLVRRRSRRSHNLSDGGSSSFKGLSSAVAGSFSKIGRGSSRMA
DSDNGMDKPEHDL
>SbANK95
MGHGVSCARTGDEHDYFRAAQVGDLDALGALLAADPSLARRATLYDRLSALHIAAANGRLEVL
SMVLDHGVPPDAVNRHKQTPLMLAAMHGKIDCVLKLLQAGANILMFDSLHGRSCLHHASYFG
NVDCLQAILTAARTTPVADSWGFARFVNVRDDHGATPLHLAARQGRPGCVQVLLENGAIVSAL
TGSYGRIPYAVALKRNHEACAALLNPSSAEPMVWPSPLKFISELDPEAKALLEAALMEANREREK
KILKGTKYSPPSSPPSTCEHEIDADAIDDVSSETSDTELCCICFDQACTIEVQDCGHQMCAPCTLAL
CCHSKPNPTTLTLPSPACPFCRGNISRLLVARASTASLDADTDKAAAAAASSPQLVRRRSRRSHNL
SDGGSSSFKGLSSAVAGSFSKIGRGSSRMADSDNGMDKPEHDL
>SbANK96
MDVASTVPFQALAYFITGEVRENGAYSVLGLLRLWRLRRVNQFFTRLEKDIRFSYFWIRCARLVAV
TLFVVHSAGCLYYLIADRYPHPEKTWIGDVIPNFRQVSVWIRYITSVYWSITTMTTVGYGDLHARN
TVEMIFNIFYMLFNLGLTAYLIGNMTNLVVEGTHRTMEFRNSIRAATSFVGRNHLPPRLKQQILAY
MCLKFRAESLNQQQLMDQLPKSICKSICDHLFVPVVKDVYLFNGVSREMLLSLVTKMKPEYIPPR
EDVIVQNEAPDDVYVVVSGEVEVILFNGINERVEATLGTRDIFGEVSALSDRAQAFTFRTRTLSQLL
RLKQATLKEAMQSRPEDNVVVIKNFLKHQVEMHGMEVEDLLGDNTGEHDNDANVLTAAMM
GNSGLLEDLLRAGKDADVGDATGRTALHIAAAKVYEDCVLVLLKHACNVNIEDAQGNTAMW
NAIAAGHHRIFNILYQFSRASNPHAGGDVLCLAARRGDLGMLRELLKLGLDVDSEDHDGATAL
RVAMAEGHADVARFLIMNGASVDKASLDDDGSGSGTARRTMSSTELRELLQKRELGHSITIVDSP
AVIPDRLQSTGSNQQRWPRVSIYNGHPFLRNRSYEAGKLINLPGTMEEFIATVGEKLKVDAEKVLI

VNDEGAEIDSIDVIRDNDKLFVVTGEDMRQLASMDSMSSSLCL
>SbANK97
MKNNSSFQSTGSAGGGGGSGTGERSGSGSLSLRYLSKIILPPLGGPPGQSQSHGGSDKWVISPLDSR
YRWWDTLMVVLVAYSAWVYLFEVAFMNASPKGGLEVADIVVDLFFAVDIVLTFFVAYIDPRTQL
LIRDRKKITFRYLSTFFIMDVASTVPFQALAYFITGEVRENGAYSVLGLLRLWRLRRVNQFFTRLEK
DIRFSYFWIRCARLVAVTLFVVHSAGCLYYLIADRYPHPEKTWIGDVIPNFRQVSVWIRYITSVYWS
ITTMTTVGYGDLHARNTVEMIFNIFYMLFNLGLTAYLIGNMTNLVVEGTHRTMEFRNSIRAATSF
VGRNHLPPRLKQQILAYMCLKFRAESLNQQQLMDQLPKSICKSICDHLFVPVVKDVYLFNGVSRE
MLLSLVTKMKPEYIPPREDVIVQNEAPDDVYVVVSGEVEVILFNGINERVEATLGTRDIFGEVSALS
DRAQAFTFRTRTLSQLLRLKQATLKEAMQSRPEDNVVVIKNFLKHQVEMHGMEVEDLLGDNTG
EHDNDANVLTAAMMGNSGLLEDLLRAGKDADVGDATGRTALHIAAAKVYEDCVLVLLKHAC
NVNIEDAQGNTAMWNAIAAGHHRIFNILYQFSRASNPHAGGDVLCLAARRGDLGMLRELLKL
GLDVDSEDHDGATALRVAMAEGHADVARFLIMNGASVDKASLDDDGSGSGTARRTMSSTELRE
LLQKRELGHSITIVDSPAVIPDRLQSTGSNQQRWPRVSIYNGHPFLRNRSYEAGKLINLPGTMEEFI
ATVGEKLKVDAEKVLIVNDEGAEIDSIDVIRDNDKLFVVTGEDMRQLASMDSMSSSLCL
>SbANK98
MDVASTIPFQGLAYLVTGEVRENAAYSMLGVLRLWRLRRVKQFFTRLEKDIRFSYFWIRCARLVA
VTLFLVHCAGCLYYLIADRYPHREKTWIGAVIPNFRQASLRIRYISSIYWSITTMTTVGYGDLHAEN
TVEMIFNIFYMLFNLGLTAYLIGNMTNLVVEGTRRTMEFRNSIRAASSFVGRNHLPPRLKQQILAY
MCLKFRAESLNQQQLMDQLPKSICKSICEHLFVPVVKDVYLFNGVSREMLLSLVTKMKPEYIPPKE
DVIVQNEAPDDVYVVVSGEVEVILFNGIDEHVKATLGTRDIFGEVSALSDRAQAFTFRTRTLSQLL
RLKQATLKEAMQSRPEDSVVIIKNFLKHQVEMHGMKADDLLGDNTGEHDDDANVLTVAAMG
NSGLLEDLLRAGKDADVGDAKGRTALHIAASNGYEDCVLVLLKHACNVNIKDAQGNTAMWN
AIAAGHHKIFNILYHFARASNPHAGGDVLCFAARRGDLGALRELLKLGLDVDSEDHDGATALR
VAMAEGHADAARFLIMNGASVDKASLDDDGSGSGAARLTMSPTELRELLQKRELGHSITIVDSP
AVIPDGGSSGHSRPGRLQGTSSDNQCWPRVSVYKGHPFLRNRSSEAGKLINLPGTLEEFKAIVGEK
LKVDAKKGLIVNDEGAEIDSIDVIRDNDKLFVVTEEDLRRLASMDSLSSS
>SbANK99
MRVPCCSLCNVRYDEEERTPLLLHCGHGFCRACLSRMLAAAPGATLACPRCRHPTAVGNSVSAL
RKNFPILSLLSASPSSPSFLHSDSGSSSDGSEDEDDFFARPSRRPAPAPAAAPPGCSSFDLVSHPDLKL
ARRIGSGPPGPAGQEVWAGMLSRGGGGGGVKRCKHQVAVKRVPLAAGDGLEVVQEEVERLRR
ASTWCRNVCTFHGAVRVGGHLCFVMDRYVGSVQAEMRQNGGRLTLEQILRYGADIARGVAEL
HAAGIVCMSIKPSNILLDAHGHAVVSDYGLSAILKNLTSRRVPDDSSAGIDATLLSPNYTAPEAW
GPLKKSLNMFWDSANGISPESDAWSFGCTLVEMCTGAVPWAGLSAEEICKSVVKEKKPPPQYSR
VVGVGLPGELWKMIGECLQFRASRRPSFQDMLKTFLRHLLDIPRSPPASPENDFTNENLPNGMEP
PTTSILEMVHDNPNALHHLVCEGDAAGVRDLLAKAASERNGSLIRSLLEAQNTDGLTALHLACR
RGSGELVEAIVAYQENVDILDKDEDPPIVFALAAGSPRCVRALVGRSASINSRLREGLGPTLAHVC
AHHGQPECMQELLMAGADPNAVDGEGESVLHIAVARRYTDCAIVILENGGCRSMGISNSQHKT
PLHLCIETWNTAVVRRWVEIASLEDIAEAIDVPSPVGTALCMAAALKKEHEKEGRELVRILLAAG
ADPTAQDDPHCRTALHTAAMIDDVELVKIILEAGVDVNIRNAQNTTPLHVALNRGANSCVGLL
LAAGANCNIQDDDGDNAFHIAADAAKMIRENLTWIAQMLLQPSPAVDVRNHRGWTLRDFLER
LPREWIYEELMETLEDKGVHLSPTMYEVADWVKFRRTVTSPAFGWQGAGPRSIGFVQSVVDNDH
LAVSFCTGEAHVLTSEVIKVIPLNRGQHVQLKPDVLEPRFGWRGQSRDSIGTVLCVDDDGILRVGF
PGASRGWRADPAEIERVEEYKVGNWVRIRPSLTVAVHGMESITPGSVGIVYSIRPDSSLLLGLCYLS

HPWLCEPEEVEHVDPFKIGDQVCVKRSVAEPRYAWGGETHHSVGKIIDIESDGLLIIDIPNRAAPW
QADPSDMEKIENFKVGDWIRVKATVPSPKYGWEDVTRNSIGIVHSLQDDGDVGVAFCFRSKLFLC
SVADVEKAQPFEVGEKVHVSPSIPEPRLGWLNETAATIGAISRIDMDGTLNIKVSGRKSLWKVAPG
DAERLSAFEVGDWVRPKPSIGSRPTYDWNSVGRISIAVVHSIQDSGYLELAGCFRNGKWLTHNTD
IEKVQTLKIGQHVRFRAGISEPRWGWRDANPDSRGIIAGVHADGEVRVAFFGVPGLWRGDPADL
EVENIFEVGEWVRLRNNVEQWRSLKPGSIGVVHGVGYQGDAWDGTIHVAFCGEQERWVGPSSQ
LEGVSKFVVGQRVRIRGCIRHPRFGWSNHSHSSIGTISSIDADGKLRIHTPAGARAWLIDPAEVEKV
EEEEEVCVGDWVKVKDSVATPVYQWGDVNHNSIGVVHRADDGELWIAFCFCERLWLCKAWEV
EKVRPFRQGDKVRIRPGLVSPRWGWGMETYASKGEVVGVDANGKLRIKFRWRDRLWIGDPADI
VLDDAHLLTEASNGF
>SbANK100
MHTPHTMNPQLLKAVSNGDADLLAQILSTTTIAEDSRCACLEGVTADGSSALHIAARHGYLKLV
EMICDQDISLIKATNNLLDTPLICAARAGHADVVDYLIQLASTQRDTEYVLRARNSGGATAVHEA
VRNGHASVLGKIMSRDASLAAMVDGQGVSPLYMAVVSNRADMVDILIRESREGSVKSPASYAGP
DGQTALHAAFFATNAHSMCKSLQRWDPALAEKADSSGRTALHYAASFGKLGVVKLLLVNSLLA
YIPDDDGLYPVHYAAMAGYSIIIREIMEICPSCDELVDKKHRSILHCAVEFGRATVVWYICVNPKF
MSIMNAGDSEGNTPLHLAVKHGHVLSFILLMMDIRVNLGIINHKGFTPLGVAWNENAHKYSFSV
FMDIYMEVSFFCCEAYSIPSDLVGNQRYNHCLEDNKKSSRFSKVSQTMLCLSVLIAAASFAAAFTP
PGNEKAGMAPLDGESYLQSYVEANTLSFYHSAIATCLLIHASLATIPIRYRSNYLRVSAVMVLTAV
VCMFEAFTSVVRLSLDPQKSFFFFCALLVQRAEYFLLCLLLIIPVCLRVLRKIKHPRPDMPFKVLAA
LASFLRIGVALYMISFVTRVQPGKQESCSGPECHDGVLLHPT
>SbANK101
MAATRHTPHTMKHELLKAVSTGDAGLLEQVLGLQSSATVEQGEESCLKGVTAEGSSALHIAASC
GYLELVKMVCAQDISLVKARNNLCDTPLICAARAGHVDVADYLMECAINEQEDLRARNLDGAT
AMHEAVRNGHVLLLQRLMSKDSGLAAVEDERHVSPLYLAVASNRADMVKVLIGESSNSVTPVS
YSGPDGQTALHAAVYISREIGESLQCWDPTLATVADDYGRTALHYATLAKNIGPVKHLLANSSL
AYIPDNEGLYPVHIASIVGNVNIVCKFMEICLNYDELLDNKRRNILHCAVEHGRIQVVWHICRNP
KSARMMNARDGEGNTPLHLAVKKGHTLIFSLLMMDTMVNLDIMNNEGLTPLDVAFSTLHSDYT
FSSFTNTSIITCLTLCEASGSPCHQARNLTDKWCLEEKKESSSYANVSRSILYISIFIVVGSVTAACTPP
GGYIAEGKDAGKPVFGERTGFWIFMVANSMSFHLSTIAIILFVFARLTRHRRFHLILSAALVFGAVL
SMVIAFAAVVGLTLDPANSWDEYILIWLVSNLAFPISLRVAMQLWTSKHRWQDISKVVAQAILVV
YVIRASIIGMQSLVKSLLLGRHESCSWPWCAIQVDSAFLYPT
>SbANK102
MAEAEADATAALSARSKVQAFLEAARAGDLQSLKSLVAVLDEDGTGAASVASAVRDANKRTAL
HFAAREGRADVCEFLIGELGLPVDPKDDDGETPLIHATRQGHLDTAKYLLDHGADPSVATNLGA
TALHHAAGIGNTELMKLLLSKGVDVESESDAGTPLVWAAGHGNSDAVKLLLQHNAKPNTEND
DGVTALLSAVAAGSLPCLEVLIKAGANPNVKAGGATPLHIAADSGNIEVIKCLLKAGADPNICD
DDELKPVQVAAWRNNREAVELLLPLTSPIPGVSNWNVDGIIEQMLPKEYGEKSQSKEAASLKSGR
TQTVEVSPEAKKRSLEAKSRGDDAFRRKDYLVAVDAYTQATELDPTDATVLSNRSLCWLRAGQA
ERALEDAKACRALRPDWAKACYREGAAHRLLQRFEDAANAFYEGVQLDPENKELVSAFREAIE
AGRKFHGVDTPSSTQ
>SbANK103
MEPMESQLTAVALSDSDSASVEGAAAADAADADLQALRRLSDNLAAAFRSPDDYGFLSDARIA
VPGAPDLRVHRCVLCARSPFLRDVFARRAASDGEEKDKGRDRGKVELRDLLGDEVEVGYEALRL

VVDYLYSGRVAALPKAACLCVDEDGCAHVGCRPAVAFMAQVLFAASTFDVAELTNLFQRRLLD
VLDKVEVDNLPLILSVANLCNKSCVKLLERCLDVVVRSNLDMITLEKALPPDVVKAIVDARLSLG
LVLPEDKGFPNIHVRRVHRALDSDDVELVRMLLKEGKTNLDDAYALHYAVEHCDSKITTELLDF
ALADVNHRNPRGYTVLHIAAMRREPKIIVSLLTKGARPSDLTFDHRKAVQISKRLTNHGDYFGPT
EDGKPSPKDRLCIEILEQAERRDPHLGEASVSLAIEGDSARGRLLYLENRVALARILFPMEARVAM
DIAQVDGTLEFTLGSNANLPTEIQRTVDLNDTPFIMKEEHLARMRALAKTVELGKRFFPRCSKVL
DKIMDDETELASLGRDTSTEKKRRFHDLQDLVHKAFSEDKEENDRSAARSSSSSSTTSIGIGAVRPR
R
>SbANK104
MQQQPQQQGLDIDKLQQVVKTRWLKPQEVLKILQNHELFTISHKTPQKPQSGSWFLFNRRVLRY
FRSDGFEWQKKKNGKTINEAHERLKVDNVDALNCYYARGDKNPTFQRRIYWMLDPAYEHIVLV
HYRDVLEGSISVSARNDSSTSNQNGSASRAEVHSSPGWTSELIVQCPNSCSPGSAEEVSSRTSASES
DWIQHKAALRKLKMQLSLEDKEDCDVNAEEVPANNAPIILPGIQNEELDTSRNHDDIFDVLDFN
EDRINGTGTHSCPSAIDVLKNSDTWLEEDQLEAILHPACMTLTENQWFKIHEISPESAFSSESTKVII
VGDFLCNPPHSSWELLFGDVKVPVEIIQQGVIRCHTPCLNAGKVRMCLVDGNGKSCSEAREFEFL
EKPTKGMIDGNRNPCNEARDSKIHQIPTKSSDELSLLLHYVQMLFDGHACGLFSNFSLPLPNLGC
EFQINQMDIIKKTYEQLDPENTVNSVMEALLNDKFKQWLSSKCEQNIDGDHFLPKQYHSIIHMIA
ALGYVLALKPLLSSGVPINYRDANGWTALHWAARFGREDMVVALLAAGAAAGALSHPTSEDP
AAKTPASIALAYGFKGLSAFLSEAQLTTHLDSIESKENGKPDSREGGICRAVDRISDKSSHVHGGT
DDQLALKDSLGAVRNAVQAAGRIQAAFRIFSFKKKKEMALQNRNSCFLSISETEAVSLSHGMLEK
AALSIQKNFRCWKKRKEFLRIRNNVVRIQARVRAHQERKKYKELLSSVGVLEKVMIRWYRKGVG
LRGFNSEAMPIDEVDEDVAKVFRKLRVETAIDEAVSRVSCILGSPKAMQQYRRMLKRYQQAKDD
HEK
>SbANK105
MEPKPHAPASGPATPRFKLGKQSSLAPERGGGEGGGVSSAEGSSAEANGVMSFQLMYLAHEGN
AEGIRELLDGGADPNFRDSDGRTALHIAACEGHAEVVELLLQRGAEAAVEDQWGSTPLADAMH
YQNHDVIKIFEKHGSKHKIAPMHVNNVREVPEYEIDPAELDFTNGNDLSKGTFRKATWRGIPVA
VKKLDDDLIVDESKVQAFRDELDVLQLIRHPNVVQFLGAVTQSNPMMIVMEFMRKGDLRTHLSK
KGALPPSYAVKLALDIARGMNYLHEHKPQAIIHRDLEPSNILRDDTGHLKVADFDLCKMLKWRK
KVREDKAVTSPGNACKYVAPEVLRNEEYDTKVDVFSFALILQEMIEGCLPYYDKKNDEIEKVHNS
KERPPFRAPPKHYAHGLRELIEQCWSENPADRPDFRVIINRLSAIQNEIVHRNRWKVRPLKCFLSIE
GMWKKDRNEGSTTRSSRSSRSYY
>SbANK106
MGHGLSCSRDGGEEHDLFRAVQLGDLDALLAADPELARHATTIYDRLSLLHIAAANGQLQVLS
MLLDDDGAGGAHAPARPDVDVLNRKKQTPLMLAAMHGRTDCVLRLLEAGANILMFDSVHAR
TCLHHAAYYGHADCLQAILSAAKASPVADSWGFARFVNVRDEHGATPLHLAARQGRPQCVHH
LLHAGAIVSAPTASYGFPGSTALHLAARRGNLDCVRELLAWGADRLHRDSAGRIAYAVALRRSH
RACAALLNPAAAEPMVWPSPLKLISELNPEAKALLEAALMEANREREKQIIVNLKGGTTTKTKSS
YSSSSAHDDDGTAVASRSQLDDDDDATELCGICLEQACSMEMQDCGHQMCAACTLALCCHSKP
NPTTLALQPPACPFCRATITRLLVANNKTSNSSDEAALGGGVRSHSHGSSSFRGLTSAIRSLSLSRIG
RRGSGRVADSDGICHGQASTPCGVNSLLPAQAIIQSFF
>SbANK107
MAGWGSSRVAACGPWGRGAALERELSRDGSHYSISSGILPSLGARSNRRVKLRPFIVSPYDRRYRC
WETFLIILVVYSAWVSPFEFGFIRKPTGALAAVDNVVNAFFAVDIILTFFVAYLDRMTYLLEDDPKR

IAWRYTTSWFVLDVASTIPSEFARRILPPNLRSYGFFNMLRLWRLRRVSSLFARLEKDRHFNYFWV
RCAKLICVTLFAVHCSACFYYLLADRYPVPTDTWIGNSMPDFHQRGLWIRYVISVYWSITTLTTVG
YGDLHAENTREMIFNIFYMLFNLGLTAYLIGNMTNLVVHGTSRTRKYRDTIQAATSFALRNQLPS
RLQDQMISHLSLKFRTDSEGLQQQETLDALPKAIRSSISQYLFFNLVQKVYLFEGVSNDLIFQLVSE
MKAEYFPPREDVILQNEAPTDFYILVTGSAELVELRNGGEQIAGVAKAGDVVGEIGVLCYRPQLFT
VRTKSLCQLLRMNRTAFLSLVQSNVADGTIIMNNLMQLLKEQKDNSVMAGVLKEIENMLARGR
LDLPVTLCFAVNKGDDFMLHQLLKRGLDPNESDNNGHTALHIAASKGDEQCVKLLLDYGADP
NARDSEGKVPLWEALCEKHNAVIELLVESGAELSSGDTALYACIAVEENDAELLENIIRYGGNINS
STKDGTTPLHRAVCDGNVQMVELLLEHGADINKQDNNGWSARDLADQQGHDDIQVLFKSRKA
HRQHASNGRVAPMLIGRFNSEPSMPNMNHEDAEVRSKVVPQKLLRKRVSFQNSLFGVISSSQAR
QDTGGLLSKGLAGTGGPSCHHDSLIRVTISCPEKGNAAGKLVRLPRSMTELLELGAKKFDFKPTK
VLTVGGAEIDEVELIRDGDHIVLVSSDWVPDVAQIRPNDQ
>SbANK108
MAAAEAAASSAPAPERRLLEVEEDEVVIDFKPNAKCRADLRLRSLHPSLPVAFKVQTSSPLKFLVS
PPRGAVPPLSTASLRVVLRPQAHPPPSFPRSRADRFLVLSSLSAARLDSAVGSDDDDVSAVRLRVFF
GGPYLLRLAADAGDAAAVRLILRRQPHLLPFLDHLQAADAAEQWAPLHAAAARGDCGEVRRL
GPDALAARDREGRTVLHAAAAAGEAEAVAVLVDMGADTSAVDARGRTPLDVAREKGYQEVV
DVLERWELVMTAARRGDLGSLESLLSKRAAVCGRDQYGLTALHLAAIKGHCDAIALLAGSGCM
DVECEDVEGHRPLHLAVEGGHAEAVEVLLDMGADVNARTRRGATPLQMAEAMGYEAVAQLL
CARGAEVAAAPALCVASSSSSSISCA
>SbANK109
MEPSSSITFASSSSYLSNGSSPCSVAMPPPGPPQAPPLAGGEGWGGGVATAGSGSSVEAVSLNRLS
KNLERLLLDRDLDCSDADVEVPDGGPPVPIHRCILAARSDFFYDLFAARGRGGALRGDATAGAG
GAAEGAASGRPRYKMEELVPGGRVGREAFQAFLGYMYTGKLRPSPVDVVSCADPVCPHDSCPP
AIRSAVELMYAACTFKIPELTSLFQRRLLNFVDKTLVEDVIPILKVASHSGLTQVIDKCIQRIARSDL
DDISLDKELPPEAVEEIKNLRKKSQTADGDGDAFISDPVHEKRVRRIHRALDSDDVELVKLLLNES
DITLDDANALHYAASYCDNKVVSELLDLALANLNLKNSRGYTALHLAAMRREPAIIMCLLNKG
ANVSQLTADGRSAIGICRRLTRLKDYNTKMEQGQESNKDRLCIDILEREMMRNPMAVEDAVTSP
LLADDLHMKLLYLENRVAFARLFFPAEAKVAMQIAQADTTEEFGGIVAASTSGKLREVDLNETP
VTQNKRLRSRVDALMKTVELGRRYFPNCSQVLDKFLEDDLPDGLDQFYLQRGTADEQKVKRMR
FCELKEDVLKAFSKDKADSSMLSGLSSSSSCSPPQKSAKR
>SbANK110
MEDTLKSLSMDYLNLLINGPQAFSDVTFNVEGRLVHAHRCILAARSLFFRKFFCGAATADQAAA
PGALLLDHLSPRSPSGASSSSSPRGGGAGAAAAAAAPGAVIPVNSVSYEVFLLLLQFLYSGQVSLV
PQKGEPRPGCGERACWHTHCAAAVDLALDTLAAARSFGVEELALLTQKQLAAMVEKASIEDV
MKVLMASRKQDLHQLWTTCSHLVAKSGLPPEVLAKHLPIDVVAKIDELRLKSSLSRRSPFLAHHH
PYHPAAGGMEASSAADIDDHHKIRRMRRALDSSDVELVKLMVMGEGLNLDEALALHYAVENC
SREVVKALLELGAADVNHPAGPAGKTPLHVAAEMVCPDMVAVLLDHHADPNVRTVEGVTPLD
ILRTLTSDFLFKGAVPGLAHIEPNKLRLCLELVQSAAMVMSREDAHTAAAAVNAAPMYGEPSGG
TVYNASGTSSSMVNLSLDNRMVYLNLGMDAQFGKMGDGSDGDDGGSRGQGGPSSLFSPHGYH
>SbANK111
MSLEIHEEDGGASGARPAKVMSVSGSGKRGRYVRQVTGRHNDTDIHVAARGGDAVALRRALSV
AAAAVAATGEGPEDLEAARRAVAAEPNEAGETPLVAAAERGHLEVVVELLRHLDAEGLAAKN
RSGYDALHVAARGHAVVQEMLHHDRMLAKTFGPANTTPLISAAMRGHIEVVVELLLEQDDFG

LVEMARDNGKNALHFAARQGHIGIVKALLEKDPQLARRNDKKGQTALHMAVKGTSCDVLRAL
VDADPAIVMLPDKNGNTALHVATRKKRAEIVSVLLRLPDTHVNALNRDHKTAFDIAEGLPVCEE
SCEIKDILSQHGALRSRELNQPRDELRKTVTEIKKDVHTQLEQTRKTNKNVHGIAKELRKLHREGI
NNATNSVTVVAVLFATVAFAAIFTVPGGNDNNGLAVVVQATSFKIFFIFNAVALFTSLAVVVVQI
TVVRGETKSERRVVEVINKLMWIASVCTTISFIASCYIVLGRHFQWAAILVSLIGGVTMAGVLGTM
TYYVVKSKRQRKIRKKEKMSRRSGSSSWYDHTELSDTELNPVYAL
>SbANK112
MAAANGMKVALHRQVSGGSMKHNAELRRQASLESPRTGRATSRFLFGRQSSMDPNRRRGRSQS
PVGSGTPAEDLSVPENLDATMQLLFFACQGDALGVEGLLRSGVDVNSINLDGRTALHIAACEGH
RDVVRILLSWKANIDARDRWGSTAVADAKFYGHSKVYELLKIHGAKVPRTKRTPMMVSVPGEIP
EYELNPGEVQFRRGCDITPGMYHIAKWNGTKVSVKILDRDGCSDQEAANSFRHELTVLEKVRHP
NVVQFVGAVTQSIPMMIVSELHEEKDLSVCIQKKGKLHAHKVLRYGLDIARGMTYLHQCKPDPII
HCDLKPKHIFLDSGGNLKIAGFGVIRVSKIGPDKVRLINHGALVDSFSYHTAPELYRNDAFDSSVD
AYSFGFILYEMVEGSVRTPEDSGHTIRFEGMRPPLKGKLKGYPPDFKALVEECWHPQAMARPTFS
EIIIRLDKIYSHCDSKGSWKESLKLWK
>SbANK113
MVLVRRSLTMSRPRSCHDADDRGWNPLHVAARKGDLKEVRRLLDDGMDVNAPAWGPKCPGA
TPLHLAAQGGHVKIMDELLERGANIDARTKGACGWTPLHIAAKERNKKAVRFLIENGAFLPPEM
NDHRFNPPLHYCSGLEWAYEMKRVQDESDSPGETSLSSDN
>SbANK114
MGFLSLVGNSFGCSASGERLVSAARDGDLQEARALLEYNPRLARYSTFGGRNSPLHYAAAQGHH
EIVSLLLESGVEINLRNYRGQTALMQACQYGHWEVVQTLILFNANVHRTDYLNGGTAIHFAALH
GHARCLRLVLADYVPSIPNFCTVMNHRPSEEDSASDFDHDALVKMVNQKADGGLTPLHMAAL
NGHVECVQLLLDLGASVSEVTIEDGTTIDLIGAGSTPLHYAACGGNVVCCQLLIARGACLTVQNA
SGWTPLMVARSWQRNSVEEILSKEPEGRIRTLPSPYLCLPLMSIMNIARECGWRYLNQSPVCIDPC
AVCLEGSCSVAAEGCKHEFCTRCALYLCSTSYTSVSPAGAIPCPLCRHPIISFITLPGTSPIRELPRNS
LSLSFCTTCPAVNSDSSSPIAAHLYRTEFQCGRMPPMMGSSSFRSLSCQRIPAMKLNPAFCMGSMD
TNPCLIRCSKFGSSLRRSASQGEETRRAWPLTFNPIVATSS
>SbANK115
MSTAHTCKPHIHRDPRTTILHCAGLLAGKPDSYARPPPLWLSPPPSLPPSLLPPAQISTSFQRAAAA
SPSPPRDTRTDMAAFTKLEDSPMFRKQVNSLEQHTDELKERCSNLHKGCKRFMGSLDEAYAGDL
SFADNLQAFGAGLDDPISVAIGGPVMSKFTTAFRELGTYKELLRSQVEHMLSDRLSQFINVDLNG
VKDCRRRLDRAAVAYDQAREKFVSVRKGTRAEVVTELEEDLHNAKSAFERCRFNLVHALANIEA
KKKYEFLESISAVMDAHLRYFKQGYELLSQMEPFIHQVLTYAQQSKQMAVSEQDKLAKRIQEFRT
EEEIANVRMASNVDTSTSGDGIHVVGLQSYKTIEALMQSTANGEVQVIKQGYLFKRPQNTRGEW
KRRFFVLDSHGTLYYYGNKGKSQGVSSQQTASEGAGVFGRFRFLNQRASSQGEDSLSCHKIDLRTS
TIKVDADENDLRFCFRIISPVKTYTLQAESGADQKDWIQKITGVIASLLNSPFPQQLSYGNVSAESN
RSASSVDSLSIEDNKSSEGHDDIFNLLRNIPGNDSCAECRSPDPDWASLNLGILICIECSGAHRNLG
VHISKVRSLRLDVKVWEPVIMDLFHALGNNYANSIWEALLPKEDQGMDESNGAILFIEKPKPND
AFSIKERYIQTKYVDKLLFARDPDQITINIFEAIRKNDVREAYRILAIAEVSANMTYDALSKDVHHV
QPVTDKMLLDPISCEIIQDSGKPEGCLQGCSLLHFACQYGHPIMVELLLLFGADINMQDFHGRTPL
HHCVQKKNDELTKHLLKRGARTTIKDGGGLTALERRMELGAITDEDLFILFVR
>SbANK116
MPGSQNGGPRPRPVKAETIHGLARAGDLAGVQRKLRENPALLNDKNPVMCQTPLHVAAGYNN

TEIVKFLLNQQGTETVDLEAKNMYGETPLHMAVKNSSCGSTNLLLEHGAHIEAKANNGMTPLH
LAVWHALQAGDCSTVSVLLSYNADCFAKDDEGKMPLNHIPGGAGSEKLLKLLTHHMEEQRKK
KALMSCVEGQAMSEFEEAISQIVGLQELKMQLRRWARGMLFDEKRRAMGLGIARRRAPHMAFL
GNPGTGKTMVARILGKLLHMVGILPTDKVTEVQRTDLVGEFVGHTGPKTRRKIQDAEGGILFVDE
AYRLIPMQKSDDKDYGLEALEEIMSVMDSGKIVVIFAGYCDPMKRVITSNDGFCRRVTKFFYFDDF
STTELAEILHMKMMSPSESSLLYGFKLHPSCSIEVIGELIARETTEERRKQMNGGLVDTLLINAREN
LDSRLDFNCDDTDTMITIMLEDLEAGLRQISRQRQLQ
>SbANK117
MDDVSKYSHSPAHLAVLRRDHAALRRLVAALPRLPRAGEVATEEESIAGEAVADAVSAVIDRRD
VPRRETPLHLAVRLRDPVAADILMSAGADWSLQNADGWSALQEAVCTREEAIATIIARHYQPLA
WAKWCRRLPRILASISRIRDFYMEISFHFESSVIPFIGRIAPSDTYRIWKRGAALRADMTLAGFDGFR
IQRSDQTFLFLGDGARPEDAGGKELHPGSLIVLAHKDKEITDALEGAGVQPTESEVAHEVALMSK
TNMYRPGIDVTQAELVPHLNWRRQERTESVGHWKAKVYDMQNVLVTVKSRRVPGAMTDEELF
AMDGEEKNGRGAELDAELDEVLTAEERKQLDSALRMGNNEEEAEERGDQGEAGADHMDANG
VAKDKKGWFGWGGKKGAKGDDKPPKVGSKDETSDPGKQKEKGSGKKKKGGSSAESLKHESEY
KKGLRPVLWLTPDFPLKTDELIPLLDVLANKVKAVRRLRELLTTKLPTGTFPVKIAIPIVPTIRVIVTF
TKFEELQPLDEFATPPSSPTQFQDAKAKESEGSGSWYSWVKGGRGTQSSDSGDSRNWKDEIDPFHI
PSDYTWVDATEKKRRMKAKKAKSRRGTARKQSSKNTSEGGHHPMMDGFEE
>SbANK118
MATPPRFPPALAGCFPRRGSPRGPDNPNPRPSSPLPGSPEDALLRAAFDGDLRLVKKMTRVLDGG
EERGAEKVRAVRSSNGLGALHLAAARGKLPVCRYLVDDLGLDVNSINEAGLTPLTCAIDAGSVD
VVQYLLNNGADTETLISTGLTPLVYAVGKGNCEIVQALLCKGAYVDALTTVGAALHLAAQNGR
DDIVKVLLDHHADHNKIAWGVNRPLIYAISARSLKCVKLLIEAGADVQGIGTETPLALAATDGLT
DILKCLVQAGADPNVCDGFGFTPIETAARYNRREDVEILFPVTSRIPTVNDWSVDGIISYAKSKPAL
KEEEERKGAADYVLQFIRPTPTHRDEDLYTVMLANLKFQGREAVKNKDYLGAVDIYTKAMNLD
PADATLFANRSLCRLRMGDGRKALTDAVACKSMRPGWSKACYREGAARMVLKDYEKACGAFL
DGLKLEPGNAEIESALWEALESLKISHKKEKE
>SbANK119
MVEELHTDVDASDDKGDTPLAYAIQGGTVDTVQYLLDHDANPDKPIGKGSTPLHLAAAGGNCE
MLKALLSKGANVDSLSDTGTPLHVAAYCKQDGAMKILLDHGADYNKVFNTLCTPLFVSLTSRSL
ECVKLLIKAGADVKGVGTVTPLIVAVNNGLTDFYNCLLEAGADPDVRDDFGHLPIELAAYENRR
KDVEILLPVTSRVPYVRDWSVDGIIDFVRSMPSVEGDPMYKMKPDDIKFEGNKAYKRKDYLTAVK
LYSMAINHRPDDTTLYSNRCLCWIKMGEGVRAFTDAGICRMQCPDSAKACYLQGAANMLLKD
YKKACDAFLDGLKLDPANVEIEKALRDAVNCLKTLDTAK
>SbANK120
MLPWAATASPPSTPAAVSFAPPLRAAAPRPPPPLCRPLPRMLCAGLNPPPRAASSDGNVFWEEPD
DGSGSDYEDDGPEQRRALRFPSSSPSSRLEAARQQEQDLRREIELLLTPEEKAILDQHETPDVTKISS
PKWHPLHSYALALQIPLMDKLLDSGVDINLLDRDGFTCLHKAVIGKKEAVISHLLRKGANPHVR
DRDGATPLHYAVQAGALQTVKLLIKYKVDVNVADNDGWTPLHLAIQSRNRDIVKVLLVNGVD
KTRRTKDGRTALDLSLCFGRDFKSYDLAKLVKLIPANRGV
>SbANK121
MASSEIEVIDAAGPAATTGGAGRAVAEGAGQEGEDEEALKDDVYTGAAYGDLEKLHRLVEREG
RSVTVPDALGYHALQWAALNNRVAAAQYILEHGANVNAIDHTGQTALHWSAVRGHVQVAEL
LLKEGAKVDAADLYGYQTTHVAAQYGQTAFLCHIVTKWNADYDVPDNDGRSPLHWAAYKGF

ADTIRLLLFLGAYRARQDKEGCTPLHWAAIRGNLESCTVLVQAGKKEDLTVQDNTGFTPAQLAA
DKNHRQVAFFLGNARRVHERECVGNGYFGKLLKVGLAPLLWCIIIALIFVYIHSIILGDYNTKMTV
SLGLFSWLGVLLATAGLFMFYRCSRKDPGYISKNIRDSQNQRDDEPLLKMGLDNPELLDGNWSQ
LCITCKIVRPVRSKHCSTCDCCVEQFDHHCPWVSNCIGKRNKWEFFMFLILEVSAMIITGVTAIIRSI
GDPASPASFGGWLGYTAINHSWVVSFVIMDLLLFFGVITLTVIQASQISRNITTNEMANAMRYSYL
RGPSGRFRNPYDHGVRKNCSEFLLKGYNEDIENTVQTLQTDEEMGPIQMRSPVSQNGESIPPHVN
GTDHGSTNSHDNSKSHRQSSSQCGNQSKKYDRIPSGLWA
>SbANK122
MASSEIEVIDAAGPAATTGGAGRAVAEGAGQEGEDEEALKDDVYTGAAYGDLEKLHRLVEREG
RSVTVPDALGYHALQWAALNNRVAAAQYILEHGANVNAIDHTGQTALHWSAVRGHVQVAEL
LLKEGAKVDAADLYGYQTTHVAAQYGQTAFLCHIVTKWNADYDVPDNDGRSPLHWAAYKGF
ADTIRLLLFLGAYRARQDKEGCTPLHWAAIRGNLESCTVLVQAGKKEDLTVQDNTGFTPAQLAA
DKNHRQVAFFLGNARRVHERECVGNGYFGKLLKVGLAPLLWCIIIALIFVYIHSIILGDYNTKMTV
SLGLFSWLGVLLATAGLFMFYRCSRKDPGYISKNIRDSQNQRDDEPLLKMGLDNPELLDGNWSQ
LCITCKIVRPVRSKHCSTCDCCVEQFDHHCPWVSNCIGKRNKWEFFMFLILEVSAMIITGVTAIIRSI
GDPASPASFGGWLGYTAINHSWVVSFVIMDLLLFFGVITLTVIQASQISRNITTNEMANAMRYSYL
RGPSGRFRNPYDHGVRKNCSEFLLKGYNEDIENTVQTLQTDEEMGPIQMRSPVSQNGESIPPHVN
GTDHGSTNSHDNSKSHRQSSSQCGNQNVFLYICAYLLDDQSTTVLFGLFLRPCSVTSLWLHTHEG
FRNSPKKMKASETSSACSYDAR
>SbANK123
MAPPPPPLRSDVAMFSAGSKKRSMLLQAAYDGDLRSFKKLLTSLDKRSGRLREKVEAVKVEADG
VLKGAGALHLAAGNGRLEICSYLVERLRVDVDAVDCGDRTPLLHALFGENVVTFKYLLDHGAN
QDKVNTDGFAPLHSAAGFGYCEMVELLLAKGAYIDPVTSCGTPLHIACTEGEDRTVKILLEHNA
DYNKMVNGMTPLYFAISAASLKCVKLLVEAGAIANGDYILNYLQDAPSNGSAECLNCFLGFGPD
WQPRNDDARVDQQKIAELKSQGDEAVEKKDFLSAAEVYSMALELDPDDATLFANRSLCWLHIG
KGGKPLLSLLDAYECKKRRPDWPKACYRQSKALMLLKEYKGACDALLEGLKLDPGNAEIEDEL
WKAVESTKLSAPTQAMCTTDIC
>SbANK124
MAPAPPRPFASIFSAAALSSSMQYMLLQAACDGDLRLFKRLVRALDKGRGRLREAVEAARTDGG
NGALHLAAGSEQLEVCSYLVEGLRVDVNVVDDEGRTPLVYAVVSENAAVVKYLLDHGADPNK
ADNDGLAPLHSAAGIGHCEMVELLLAKGAYVDPLADECGTPLHLAAKERQAGTMKILLDHKA
DCNKTYMIYGLYGMTPLFQAINVSSVKCVKLLVEAGADVSSDCVLTSLTDSNLGNEGSTECLNFL
LDAIASSNVPDDDHVKKRKISLKSLGSKAVEKEDYFSALSFYSKAMDLDPDDATLFSNRSLCWLR
MRDGQKALLDSLACREMRPDWPKACYRQGAALMLLNDYKSACQAFFDGLMLDPKNTEIENAL
RQAMQSLKVSEGTKVK
>SbANK125
MCDAFYDISAQLGPILEKDSVRSFLRLFVNEPGMSWDFAITAQTLTYMVGYNSLRCAKVALEGKA
PELNRRRANPNCINPYGYFPLHEAAERFSIDMIKLLFRHGASANVRTIGANIIEDLLPLHVAVDNT
CLHKYLEDNMYPIQYHQNYIYKLIHLLCLPEMKIFLETTRLLATRTDNLIDELWKYMEDGKLIQSA
VLLLSAQEQIRGGCSTKINGNNKQGGFNILMHRIMRNSIALRWEKSENGNSRKQLEEKRALLDCT
ALLIDIIYQAGEVLSAYIQTHSEVPHVNVLDRVSSILGEFGFHTTGGIIDANNLHPYDCIMTGNDLR
RTDVTKAITETANLHDAKGKTQRKKLPKGWDPTYMKRRFFPFWRSVLRSQSPLKIYPRYAATDA
DSFPDIELFNTNPSISSSSTSIPNRKMSLVGRAPQLASNLHSMRLYATAATKLLKVLKNA
>SbANK126

MDLPPLSHQALFAAVRSADAAAVRALLADAEAPGSGTSLAALAAAQTDAGETALYVAAEAGSE
EVVRLLLPLYDFEAATVRSRLDLDAFHVAAKQGHTGVVKEFLGRWPGLCSVCDSSNTSPLYSAA
VKDHLDVVNAILDTDDSCIRIVRKNGKTSLHTAARIGYHRIVKALIERDPGIVPIKDRKGQTALHM
AVKGKNTDVVEELLMADVSILNVRDKKGNTALHIATRKWRPQMVQLLLSYDETLEVNAINSQN
ETAMDLADKVPYGESKTEIIEWLTEAGAKNARNVGKIDEASELRRTVSDIKHNVQAQLSENAKT
NKRVTGIRKELQKLHREAIQNTINSVTMVATLIASIAFVAIFNLPGQYFQDVNSGGDIGEAHIAKL
TGFRLFCLLNATALFISLAVVVVQITLVAWETGAQKQIIKIVNKLMWTACLSTGAAFISLAYVVVG
PQHAWMAFTVSAIGGPIMIGTLLFLAYLLLRPRFKFGEDRQRRIKRASGSKSFSWSLHDGFSDLEA
FSDHEKKIYAL
>SbANK127
MFEQGKPRSIRAKAKKSPFVEVEADEIGQASGVVFARESALAPKNDQGCGVSHSHGDDETQESD
GCVQSQGLDEHGWVDFGHSLQLVLFSRQWSLAESLVDLADQQSMLDYGLSIALDAIWFLRTKRD
LEGLNSLIVKIVASGAKDFARAILRTSLLASCVAACQSKAITVGDSKEIVAERLHERLRDCPGAEHL
KIEAGAKVQKFMEWALQCIHMHHCSEDTHRCRWSCNTLQEVQLHLSAFRTFLDIAGDNLSGKIF
TEAFDAVCFPLTLFSSLFEPGWSSGSSAVSIKGLLSLLVEGGADNVNQCFLEAARFGSTELVRILLEV
AYQNSLAVDTELALVYASHYCKFNTMECLVDEGNVNSFLSPLIKAAERGCLQVVHWFVSRGISEI
EMCLALTTAASSGHFMVASYLLAHIPQQILEALSTQILKAARGQGSRSLDGVTFLLRSNFLRDAAA
TYEAADIIATGGTNGEPPDLVAFLKEHWSQSAFAEGVTTGEMHFVNVMRVLRRGASPICLEDLPS
EMVLGITYLPLYRECVSAGGQLLPQRLRGELLEAVHRLGEPAGTESQGKDLVRTLERHMPSFLVG
S
>SbANK128
MEQTCCYFPLRWESTGDQWWYASPIDWAAADGHYDIVRQLLHLDPNLLIKLTSLRRIRRLEALW
DDDARFADAARNRASVARSLLLECECRNPNHHRAGGENTLLRAGYGGWVLYTAASAGDAAFV
RELLERDPLLVFGEGEYGVTDMFYAAARGRSADVFRLLLDHAMSPRCSTDCRNGQGGAGRGSM
FRLEMMSRAVHAAARGGSVEMLRELLEEGPSSVSTYLDIRGSTVLHAAAGRGQLQVVKYLLASF
DIINLTDNHGNTALHVAAYRGHQPVVEVLVAASPSTLSAVNNAGDTFLHSAVTGFRTPGFRRLD
RQLELMRYLIRERTADIQKIINLRNDAGLTALHLAVVGCAHPDLVELLMTAPSIDLNAEDANGM
TALALLKQQLRSATSDRLIRQIVSAGGVLNSSILRTRSAIASQIKMQGGIASSPGTTFKVSDAEIFLFS
GMGAVESLRPSSCSSNGKDDPTHADADCAENHGSSEKRLSSASRAKDRLKMMLRWPRHKTPKK
SEDSSPLDSIKKLSQHEAETPAPLRQKFTKTTALNGKRTLAVKSSTPSSSSTKKKLNTKLIHGIMEA
MPQLAPATVRSGSPTDMLRRSSISSTPQPLAKLKDICLDDEISMVTPSVGKLKDIVLDSDDTTEDPS
CSNSSMDDGCGTGTAESAARKHGCGNGRLINICFGAQGLTVEDSASGQQTSKMFKQQCLRVS
>SbANK129
MEEMQVATTEEPRDPFLPLRWESTGDQWWYATPIDWAAASGHYDVVRELLRLDPNLLVKLTSL
RRIRRLESVWDDDARLRDAATNRAAVARRLLLDFGEPADKKHHHHLPRGGGGDADADANAN
RLLRAGYGGWLLYTAAAAGDERFVRELLAAQPLLVFGEGEYGVTDILYAAARSGCPEVFRRLFD
AVLSAASCPVEGEEFRREMMCRAVHAAARGGSLDVLRDLLRGCDDAAAYRDAQGSTILHAAA
AKGQVEVVKDLFASFDIVDSVDDQGNTALHIAAFRGHLRVVEALVTASSSLISATNEAGDTFLHM
ALTGFGTPEFRRLDRQMELIRQLVGGAIVDISSTINAQNYDGKTILHLAVVGNLHPDLVEHLMSV
PSIDLNICDNDGMTPLDLLRKQPRTTSSEILIKQLILAGGFTNSRDHETRSVIASQIKMHSIVGSPGTS
FKISDAEIFLDAGIDVPGISERAVSFSSIIGRVDADILRPKLKKLNSFQDAAKHIKVLLRWPHRKSKR
SGSGQKDLDDDASSVDSAKTWSHEETPTPLRQRYSRISSLFNKRTYAGKTSPSEAMQKSGVVQPHS
IPASTSWSSSSLVDKIEAAHLDKDQSPYISRLIRHTPKKTGSLNSRLMNQSLRLRA
>SbANK130

MDGAAKKKMTRSISRQLSSGAARLWRQLSLDPHTPRRGGPGPGAGAGARQTRFAIARQSSLDPT
PRGGPDGSSAHQQLAVPENLDATMRLLFAACQGDAAGVEELLRSGVDVDSIDLDGRTALHIAA
CEGQGEVVRLLLDWKANINARDRWGSTPAADAKHYGHFEVYNLLRARGAKVPKTRKTPMAVS
NPKEVPEYELNPLELEFRRGEEVTKGTYLAKWYGSKVFVKILDKDSFSDADSINAFKHELTLLEKA
RHPNLVQFVGAVTQNVPMMIVSEYHQKGDLASYLEMKGRLKPHKAIRFALEIARGLNYLHECKP
DPIIHGHLSPKNIVRDDEGQLKVAGFGSLSLTKVSEDKVQMVQPVTKLDNVYIAPEVYKNEPFDR
SADVFAFGLILYEMIEGTPAFHPKPQEEAAKMICLEGLRPPFKNKPKYYPSDVKELIQECWDTTPS
VRPTFAEIIVRLNKIHASCAKQGRWRDTFKLPWKQTGDR
>SbANK131
MPAAATDGASALHVVAASGDAQGYLDLARLVCCKAPELLLACDGNGDTPLHCAVRAGNAEM
ASLLIQEANGCVERKTMLRMTNKRGETALHEAVRFRHDTGLRMVKALMSHDKELARMVARDG
TSPLYLAVSLHHSAIAFELLSQDKELSYSGPLGQNALHPAVLRSKKMTQELLKRNKDLTKQRDLS
GSTPTHFAASADDPSLEFFLYVFVERTLEFYSLGIYFAPANWLTRFYSFLNLPLYQLVQADPSSAFQ
RDNDGLFPVHVAASAGNLVAVIILLILCPGCSGLRDSLGRTFLHVAVEKRSHNIVKFVRMRPEFDS
ILNIQDSQGNTALHLAILEGHLCIFQTLMMNPHVRLNLPNHDGKTPMDLAESRAPPGFYFGMHA
QRRILGTLTFVNAQNGNSRRDRFKEKLVPKLDKAEESKKITEFAQIVGICSVLVATATFAVVFQLP
GGLRTDDNDNSPHDNAPSQSPSPAPGHPIGTPIMAGKYAFDGFILANTLAFSCSTIATFSLVYCGM
AAVDIDKRIKLVSISIALLNGAARSFCAAFAFALYLLLSPVEPATAIATATMTALVLLDAVRFLWLL
FVDTVIVLTRGRGAAPLLKLTTAFIANMVYLFWPYIIIFSLLGGHNNSALHSS
>SbANK132
MAATETEVIGSSVGMDPALYKAATQGSVRSLRKLVVRDVKILNSKTPQDNTALHLAALHGHAEF
AREVLAVSEELMKNNSHVVELLLIRKTELAYSRNKDRQSPRHVAAQYGSTDVIKALLRHCSDVAE
MEDGNGRNAFHASIISGNESTIRCLLRHVRPTELLLNRVDGYGDTPLHLAVKMSRVHFALLLLND
VRVVDPCVRDYQGQTARSLVEKKLNTDETDTYEMHLWTQLMQQESKRCSRQQLPPTVSDRRRP
LNSKDFDSVVDAYFLAATLIATVTFAATFTMPGGYDQAKGIALHGNNRVFKTFVISNSVAMCSSI
VVIFLLIWARQEPAILRLHYLAWSQKLTIVACLAMLLSLMTAVYITVAPTAPWPAYAVIAIGICSPG
LFFVISWMGKATINVH
>SbANK133
MPASIDPCPAANCQIMEAGKKTATMMDSRLLDAAVSGDTTMMKHLALHDPAVLLGTTPRGNT
CLHISAMHGHAGFCMDAMALNRSLLSAVNNDEETPLVATVTRRLYSFYIFQI
>SbANK134
MSSSNGAEDSPSETNALVEAMDAKLMMATGTGDVQQLKHLVHRQEQDSNMMVVVMAKQTA
SVEKPCPQQRNMDPHLLALASSGSSEELQTLLNGENSQATGHGTDGSLTTLRASSYGGDTEANES
ILAWMTAQGDTALHVVAACSQGNNLFTRFREAKHILVEQNNKKRHPTRAERHAMTTYGDGDN
FLESTRIIYGKAKHLLFVQNNKGDTPLHCAARAGKSNMVACLIDLASSEGENRIKELLRKENKHK
ETALHEAVRVGNKDIVDLLMWKDSELANFPEDGGTSPMYLAILLKWDEIVKTLYDKSSHGKLSFS
GPNGQNALHAAVLRHQAQDLAKLLKWNETHLCPQNKDLTTQRDETGSTPLHFAAAVKFLFRP
SNICRQVLEANPDALYQPDHAGVFPIHVAASAGASWNVDMFVKRCPGSAGLCDAKGKTFLHV
AVEKKEANVIRSVCRNLSLSWIMNMVDNDGNTALHLAVEAGSLQMFCPLLANPQVNLNLPNSR
GETPLDIAQYKIPEDGFYHALNSEVQICHTLRIASAVNGVRRHGHLKDNKTVRVKHDESKEMEA
VKDSTGSLCIGSVLIATVTFGVTFAVPGGYVADDRNNGGTPIHARRYAFDAFIASNTLAFVLSTMA
TLGVMHSGSSLLSLQRRKMHIFIAIYLVSNSITSLTAAFALGAYVVLAPVAQKSAIATCVLSSLVLLY
MNMEFIWRRLLLLPPLRTRKGLIWAWSYSAYVIAASMLSGYWPLIFIFGWAAYTNQSIT
>SbANK135

MSSSNGAEDSPSETNALVEAMDAKLMMATGTGDVQQLKHLVHRQEQDSNMMVVVMAKQTA
SVEKPCPQQRNMDPHLLALASSGSSEELQTLLNGENSQATGHGTDGSLTTLRASSYGGDTEANES
ILAWMTAQGDTALHVVAACSQGNNLFTRFREAKHILVEQNNKKRHPTRAERHAMTTYGDGDN
FLESTRIIYGKAKHLLFVQNNKGDTPLHCAARAGKSNMVACLIDLASSEGENRIKELLRKENKHK
ETALHEAVRVGNKDIVDLLMWKDSELANFPEDGGTSPMYLAILLKWDEIVKTLYDKSSHGKLSFS
GPNGQNALHAAVLRHQAQDLAKLLKWNETHLCPQNKDLTTQRDETGSTPLHFAAAVKFLFRP
SNICRQVLEANPDALYQPDHAGVFPIHVAASAGASWNVDMFVKRCPGSAGLCDAKGKTFLHV
AVEKKEANVIRSVCRNLSLSWIMNMVDNDGNTALHLAVEAGSLQMFCPLLANPQVNLNLPNSR
GETPLDIAQYKIPEDGFYHALNSEVQICHTLRIASAVNGVRRHGHLKDNKTVRVKHDESKEMEA
VKDSTGSLCIGSVLIATVTFGVTFAVPGGYVADDRNNGGTPIHARSSGGPEERNCHMCPQFSRAA
IYEYGIYLETSSSTTTVAHEEGANLGMVIFSICYRG
>SbANK136
MAEIEVVSEGGGGRRDEDEERRRRQEEGAGQPADPVVDVYSAAAYGDLERLRGFVEQGGPAAA
ALREPDGNGYHALQWAALNNYPHVALYIIEHGGDVNATDHARQTALHWAAVRGSTSVADVL
MEHGARVEAADVNGYRAVHVASQYGQTSFLHHIISKYGADFDALDNDGRSALHWAAYKGNA
DTIRLLLFMDANQVRQDKNGCTALHWAAIRGNLEVCTVLVHTGTKEELTLKDSGGLTPVQLAA
EKGHRHLSNILSNATKVSFEDKFFPGKSRKFGYAPFLFAYLVVCLILFLNSIVFAPNFSRITATAGL
WSWAAVSLFFASQVMFYRVSRKNPGYIKANSKRSDPKEPLMEIDLTNSSTWTGNWSQLCPTCKII
RPMRSKHCPTCKHCVEQFDHHCPWISNCVGKQNKWDFFVFLWMGIATSFLGAAVGFHRLWTE
PIILSSSETWMHFMVTKHPGAVLFMFLDVFLLTGALILTGAQAVQIARNLTTNEVANRSRYTYLRG
PDGRFRNPYSRGCQRNCTDFLINGYSNDEEIAWPTLQQTVQGR
>SbANK137
MAEIEVVSEGGGGRRDEDEERRRRQEEGAGQPADPVVDVYSAAAYGDLERLRGFVEQGGPAAA
ALREPDGNGYHALQWAALNNYPHVALYIIEHGGDVNATDHARQTALHWAAVRGSTSVADVL
MEHGARVEAADVNGYRAVHVASQYGQTSFLHHIISKYGADFDALDNDGRSALHWAAYKGNA
DTIRLLLFMDANQVRQDKNGCTALHWAAIRGNLEVCTVLVHTGTKEELTLKDSGGLTPVQLAA
EKGHRHLSNILSNATKVSFEDKFFPGKSRKFGYAPFLFAYLVVCLILFLNSIVFAPNFSRITATAGL
WSWAAVSLFFASQVMFYRVSRKNPGYIKANSKRSDPKEPLMEIDLTNSSTWTGNWSQLCPTCKII
RPMRSKHCPTCKHCVEQFDHHCPWISNCVGKDFGQSPLYSHLQKHGCISW
>SbANK138
MDHRRQHQHCVQLACPPGDSPATKPMMRHPKDDAAHLFISMCPSLYRAVYRGRTEEVMELLL
RQKGAARDGKAAAGIRHGQCDILEVSAESNTVLHVAAEQGHDKLIQELYHRFTEHGLLLSHRNS
ALDTPLHCAARAGHVMAVAVLVKLSRGSGESILGCRNQAGDTALHLAARHGHHVVVAALVSA
AAGPAADLNNAGVSPLYLAVMSGSVQAVKAITKCKDASSAGPSSQNALHAAVFQSSEMVDVLL
GWRPALADQVDSSGSSPLHFASSAGDRSVVHAILRAAPPSTVYKKDSSGLSALHVAARMGHHRV
AKEMLRMYPDAGELRDGDGGTFLHTACREKQASVVSSVAIKSRRLRGLLLDARDGGGNTALHL
AVAAGAPGVVEDLLRKGGARADVVNDDGDTPFDLLAAASTTSSFTMVRLVVTLVAYGAQLGST
RRQDQLAPWSGRDVVQGVERTSDSLAVVAVLIAASAFAAGFNVPGGYDSGGTGRALLEGKSPAF
GTFLFLDMFAVATSVVAVILLVYGKTSRSAVASFTSFAWALQCMWVSLMTLMLAFYVALAITSAV
SRYGLMAIETCIFALQMCVTTWIMPAKSWSTIWRFIVRQRISHDKGKNVIIKRQYPLAGAFVLHFC
LFTVITNIAGLIFWVTTDGRQGTGTPVVLSPTPSPVV
>SbANK139
MDHRRQHQHCVQLACPPGDSPATKPMMRHPKDDAAHLFISMCPSLYRAVYRGRTEEVMELLL
RQKGAARDGKAAAGVSGIRHGQCDILEVSAESNTVLHVAAEQGHDKLIQELYHRFTEHGLLLSH

RNSALDTPLHCAARAGHVMAVAVLVKLSRGSGESILGCRNQAGDTALHLAARHGHHVVVAAL
VSAAAGPAADLNNAGVSPLYLAVMSGSVQAVKAITKCKDASSAGPSSQNALHAAVFQSSEMVD
VLLGWRPALADQVDSSGSSPLHFASSAGDRSVVHAILRAAPPSTVYKKDSSGLSALHVAARMGH
HRVAKEMLRMYPDAGELRDGDGGTFLHTACREKQASVVSSVAIKSRRLRGLLLDARDGGGNTA
LHLAVAAGAPGVVEDLLRKGGARADVVNDDGDTPFDLLAAASTTSSFTMVRLVVTLVAYGAQL
GSTRRQDQLAPWSGRDVVQGVERTSDSLAVVAVLIAASAFAAGFNVPGGYDSGGTGRALLEGKS
PAFGTFLFLDMFAVATSVVAVILLVYGKTSRSAVASFTSFAWALQCMWVSLMTLMLAFYVALAIT
SAVSRYGLMAIETCIFALQMCVTTWIMPAKSWSTIWRFIVRQRISHDKGKNVIIKRQYPLAGAFVL
HFCLFTVITNIAGLIFWVTTDGRQGTGTPVVLSPTPSPVV
>SbANK140
MCTCMLRWILALIKRGVLILDMQEKEFEVFETGIYKHGQCSIHEVTAAGRNTVLHVAAAQGHHE
LIRELYLRFRDHQGLLSRRNLALDTPLHSAARAGHAAAVKMLAEGAHSVVKDLLCKGKVRTNV
LNKDGHTALDLAAGSTNFFTMVSLVVTLVAYGAQLRPQRQDHLKPWMSAAACQGQGKGIDKT
SDSLAVVQRDHGPGHPRSQGHLQVVPLPGHRGRGVVRGRGGPARLRQGIALRGLVEELRAGAA
LHVGVAREPFPGLQRGADSRGERKGGLLLRLLGHLRGRLFPHHLHRQVDRASHWVTHSLEVSTS
VQAIQSTACHQAAVSARRRYRSQLDSLFGYKFISLRRRCYHSCNVLQRGASATAHLTCTCTCTFST
FPLEKSSMHVYIASCIHCYWRLGLLPSSVSNFAEC
>SbANK141
MLHQHVPFPPWHGLHRGDDGGNGDASETSRSGRLSTHRGVLILDMQEKEFEVFETGIYKHGQCS
IHEVTAAGRNTVLHVAAAQGHHELIRELYLRFRDHQGLLSRRNLALDTPLHSAARAGHAAAVK
MLAEGAHSVVKDLLCKGKVRTNVLNKDGHTALDLAAGSTNFFTMVSLVVTLVAYGAQLRPQR
QDHLKPWMSAAACQGQGKGIDKTSDSLAVVQRDHGPGHPRSQGHLQVVPLPGHRGRGVVRG
RGGPARLRQGIALRGLVEELRAGAALHVGVAREPFPGLQRGADSRGERKGGLLLRLLGHLRGRL
FPHHLHRQVDRASHWVTHSLEVSTSVQAIQSTACHQAAVSARRRYRSQLDSLFGYKFISLRRRCY
HSCNVLQRGASATAHLTCTCTCTFSTFPLEKSSMHVYIASCIHCYWRLGLLPSSVSNFAEC
>SbANK142
MYLAAKENKIEVLRVLLEHDPSLGYFTSTDGSPLLCIAATEGHVGVARELLRHCPDAPYCDATGS
TCLHIAVTFGLADFVRFVVRSPQLQHLVNLPDNKGEPALLLAHRIMAAGDEPKRKTMMSVVTIL
QCYNKYPRRAFHVAVLR
>SbANK143
MMDSRLLDAAVSGDTTMMKHLALHDPAVLLGTTPRGNTCLHISAMHGHAGFCMDAMALNRS
LLSAVNNDEETPLVATVTRRLYSFYIFQI
>SbANK144
MERLVEASVGGHVAEMKHLHLRDPGVLLGTTPQGNTCLHIAAIHGHEVFCKEVQALKPSLLAA
VNSDGETPLLAVMASGHVSIASVLLRCCRDQQLSETILKQDKRGCNALHHAIRCGHRELALELIK
AEPALSHAVNEYGESPMFAAVTRNYEDVFDKLLEIPNSAHGGACGWNALHAAVRKGNSGYQIS
GPSIPLLDYAAFNGHVDVARELLKHCPDAPCCETTGSTCLHRAVWSEQPEFVKFVLGSPQLQKLV
YMREGECGDTALHLAVHKCNPKMVALLLNQSIDVTVFNKAGYSANWLLSTDRAKTLNWHVK
DQVTDISRKDIKSLTQTYTGNTSLVAILIATITFAAAFTLPGGYSTDPGNEGLPIVAREIAFKAFLFSD
TLAMCSSFAVAFIYAL
>SbANK145
MDPQQQSIIQLAAGASPANPALWPMQLPQCGHERLVGNNMRPSLYLAVFKGSMAEVEKLLPQQ
HAAALDCEATGIVMHGQCDILELTAERSTALHVAAEQGHDELIRELYHRFRDQGLLSRRNSALD
SPLHCAARAGHDRAVNVLVEVARDCGENILGCKNEAGDTALHLAARHGHGATVEVLVSAAA

GPAAELNNAGVSPLYLAVISGSLRAVRTIITKCKDDASSAGPNSQNALHAAVFQSSEMVDVLLG
WRPALADQVDSSGSSPLHFASSDGDLSIVRAILRAGPPGTVYKKDSSGLSALHVAARMGHHRVIK
EILGSCPEAADLRDGDGGTFIHAAAREKRSSVVSLATKDPMLRGLLDAQDSDGNTPLHLAVAAG
STGIVEDLLHEGKVRANVLNNDGDTAFDLAAGSTTSFFNMVSLVVALVAYGAQLRPQRQDQLK
QWGGRDKVRKGIQNTSDSLAVVAGLIVAAAFAAGFNLPGGYGDNGKANLRGDLVFKSFLVLNT
GAVTTSVVAVILLVYGKAASHSAGSWKSFAAALQLLWMSLVFMMLAFQAALLSVATTKSLTYGF
QVVYTCILVLQVWIVKWLGVTLTRLHTIWRFHSQGRKHFDVKRQYPFVKATVLNLQLFIATSFLIS
IGFQAIFGLNRRSMDTSIAPSPSPLCF
>SbANK146
MDPQQQSIIQLAAGASPANPALWPMQLPQCGHERLVGNNMRPSLYLAVFKGSMAEVEKLLPQQ
HAAALDCEATEMVDVLLGWRPALADQVDSSGSSPLHFASSDGDLSIVRAILRAGPPGTVYKKDSS
GLSALHVAARMGHHRVIKEILGSCPEAADLRDGDGGTFIHAAAREKRSSVVSLATKDPMLRGLL
DAQDSDGNTPLHLAVAAGSTGIVEDLLHEGKVRANVLNNDGDTAFDLAAGSTTSFFNMVSLVV
ALVAYGAQLRPQRQDQLKQWGGRDKVRKGIQNTSDSLAVVAGLIVAAAFAAGFNLPGGYGDN
GKANLRGDLVFKSFLVLNTGAVTTSVVAVILLVYGKAASHSAGSWKSFAAALQLLWMSLVFMM
LAFQAALLSVATTKSLTYGFQVVYTCILVLQVWIVKWLGVTLTRLHTIWRFHSQGRKHFDVKRQY
PFVKATVLNLQLFIATSFLISIGFQAIFGLNRRSMDTSIAPSPSPLCF
>SbANK147
MDWRLLKAATSGDVPSLKQLLALDGPGVLLGITPQGNTCLHIASIQGHEEFCKNILTVDHSPALA
LLSTINKDGETPLLTAVARGCAPLLAAVLLRRCRDQQLMQTILKQDRRGCNALHHAIHIGYTKL
ALELIEGEPAMSKTVDERNESPLFNAARRNFTEVSHKLLEILDSADCGPLGVHVLHAALAIHGDE
ELDTLIKGTIVGGGVILHTHKSLINKTTKE
>SbANK148
MPASIDPCPAANCQIMEAGKKTATMMDSRLLDAAVSGDTTMMKHLALHDPAVLLGTTPRGNT
CLHISAMHGHAGFCMDAMALNRSLLSAVNNDGETPLVAAVRGGRTSTTSLAPSFLRCYRDLHLS
EAILKQDKQGNNALHHAIRSGHRELALELIAAEPALSKAVNKYDESPMYIAVMRNYKDVSEKLL
EIPDSAHLGGTNGHNALHAAVRNGTAAIAKKIVETRPALALTEDKIRKATPLHQAVLWDKVDV
LRVILEHDRSLGYVVSSKGTPLLVSAAYRGNVGVARELLKHCPDAPFAKTNGWTCLHQAVWNG
QLEFVDFVLGLPQFGRFLINMRDQDGDTALHLAVQKSNPKMVAALLLHRDIDVRVLNDNGNEA
IWKLWNVTKDSKTLNWNEISMLMLKADPQAATDIYNLRREAHDKVTETTRNDIKSLTQTYTGN
TSLVAILIATITFAAAFTLPGGYSADPGNEGLPIMARKFAFKAFLISDTLAMCSSLVVAFVCIIARLE
DLEFLLHYRSFTKKLMWFAYMATTTAFATGLYTVLAPRLLWLAVAICVLTTSLPILTKLLGEWPIL
RLRLRLGRTFKSDLLDMV
>SbANK149
MSSEDSLKSLSLDYLNLLINGQAFSDVAFSVEGRLVHAHRCVLAARSLFFRKLFCGLDPNHQPPPP
PPGSSAGRAAPPDLVIPVSSIRYEVLVLVLQFLYSGQASVAAPKSGPLPGCGARGCWHTRCGAAV
DLALDTLAAARSFGVEQLALLVQKQLEAMVKEASVDDVMKVLMASRKFEMQELWATCSHLVA
RSGLSADLLAKHLPIDVVAKIEEIRAKSPAVVSPAGSGGGPRSPFLTHHYLPINAASSAADRDHRIR
RMRRALDAADIELVKLMVMGEGLDLDDALAVHYAVQHCGRDVVKALLELGAADVNSRAGPA
GKTALHLAAEMVSPDMVSVLLDHHADPNARTLDGVTPLDVLRSLTSEFLFKGAVPGLTHIEPNK
LRLCLELVQSAVMVATRDDGAGTGTGGGDTGGGGSDGGGSFPRGDAADDSLVSLTMNSTLMY
QGQDLAAAMAGGEARKGSGGGGRGGSPSNLYFPNGFP
>SbANK150
MYTPVHLAAYENKVDVLTVLLEHDPFLGYLISTYGVPLLCMAASSGHVGVARELLKHCPDAPYC

NANGLTCLHVAVLNGQAVFVEYVLGSKQLQSLINTADSSGETALHLAQRNGLMLIAASLGDKE
KITNNYPINEGPGITRSNSDSWNHSTGYNALHAAVRNGNVVMAKRLMEARPWLVRQENEDKR
TPMNLAAHKNQIEVLRVLLQHDPSLGYFISTDGSPLLCIAATEGHVGVARELLRHCPDPPYCDAT
GSTCLHIAGSTGQGNFHLINLPNDRGETALRLAGRMLPAGDEQKTQTMMSIFDVLLCYRRDLLA
EPFTSSSSGEHAVKAPYAGSKARSGSATVGS
>SbANK151
MESAQGQAISKDELPDCMFMCSELYIAAFEGRTEEVLIGLQTGGVHTATAARNGRRRPSPGDATP
TVIISGTQHGPCCSTREVTADRSTLLHIAAGQGHRDLVTEVGLRDGALLFAANSSLDTPLHCAAR
SGHAGAIEAIVRLARRDVDDADRRLREELLGRRNRGGDTALHVAARHGHGEAVEALMKLAPEL
AAGVNGAAVSPLYLAVMSRSVRAVEAILGYRDASAAGPMSQNALHAAVFQSSEMVSLLLRWKP
RLATDLDGQKSSPLHFASSDGDCAIIKEILTYAPPSTAYLQDREGHSALHAAALMGNGPAVKLLL
QFYPASADIRDNQGRSFLHAAALRGHSSIVSYVIKNRMLENLLNVQDQEGNTALHLAVQAGEYR
VVSKLLSSGKMQVHIMNNEGCTPSDQIENSTSFYSMVRLVVMLNVYQAQFRPQRQDHVEKWAG
QDLVKWRLATSKNLAIVSTLVATVAFSAAFNVPGSYGSDGKATLNGNRMYNAFLVLDTIAVTTA
VVATILLVYGRASSRSHHSWLDFIISMHFLWLSLLSMMLGFFAAIAATSDKNSTLIALVRLIYSGLY
VLTLLLTILGSPGTLRGVLRLLFGRKQHLKRRINRQYPFVVIYAFNMLLFVVISNVAIAAVETIGSVS
TSSRNHV
>SbANK152
MESAQGQAISKDELPDCMFMCSELYIAAFEGRTEEVLIGLQTGGVHTATAARNGRRRPSPGDATP
TGTQHGPCCSTREVTADRSTLLHIAAGQGHRDLVTEVGLRDGALLFAANSSLDTPLHCAARSGH
AGAIEAIVRLARRDVDDADRRLREELLGRRNRGGDTALHVAARHGHGEAVEALMKLAPELAA
GVNGAAVSPLYLAVMSRSVRAVEAILGYRDASAAGPMSQNALHAAVFQSSEMVSLLLRWKPRL
ATDLDGQKSSPLHFASSDGDCAIIKEILTYAPPSTAYLQDREGHSALHAAALMGNGPAVKLLLQF
YPASADIRDNQGRSFLHAAALRGHSSIVSYVIKNRMLENLLNVQDQEGNTALHLAVQAGEYRVV
SKLLSSGKMQVHIMNNEGCTPSDQIENSTSFYSMVRLVVMLNVYQAQFRPQRQDHVEKWAGQD
LVKWRLATSKNLAIVSTLVATVAFSAAFNVPGSYGSDGKATLNGNRMYNAFLVLDTIAVTTAVV
ATILLVYGRASSRSHHSWLDFIISMHFLWLSLLSMMLGFFAAIAATSDKNSTLIALVRLIYSGLYVLT
LLLTILGSPGTLRGVLRLLFGRKQHLKRRINRQYPFVVIYAFNMLLFVVISNVAIAAVETIGSVSTSS
RNHV
>SbANK153
MAQPQSRQEQSATPMDRRLLAAATTGDTIAMQHLASHDPAVLLGTTPQRNTCLHISCIYGHEGF
CKDVLTLNQCLPLLAATNADDETPLLAAVARGRAKLASILLRFYCDQHLRDTILMQDKRGFNAL
QHAIFRGLRKLALELIEAEPALSKSVNKWYGSPMFTTVMIDNGDVFEKLLEIHDAADGGARRYN
ALHAAVTTGNAVMAKRIMEARPLLVRQENDDKRTPMYLAAKENKIEVLRVLLEHDPSLGYFTST
DGSPLLCIAATDGHVGVARELLRHCPDPPYCDTTGSTCFHIAVTSGLADFVRFVVRSPQLQHLVN
LPDNKGEPALLLAHRIMAAGDEPKRKTMMSVITILQCYNNNNLSVHAEPFTSGMERLVKAAVC
GHVAEMRNLYLHVPDVLVRTTPQGNTCLHIAAIHGHKVFCKEVQALKPSLLAAVNSDGETPLL
AAVASGRVSVASVLLRCCGDRQLSNTILKQDKRGCSALHHAIRSGHRELALELIEAEPALSQAVN
EYGESPMFIAVMRNYEDVFNKLLQIPDSALGGACGMNALHVAVRNGNSDIATKIMKTVPALAR
EEDTVTKTPVHLAALYDKIDVLGVLLEHDPSLGYLVTGDGFPLLSLAGFRGHVGIARELLKHCPD
APSTSVSGWTYLHTAVSYQQTEFVDFVLGLPQSGRLVNMRTKDGDTALHLAVQKCNPKMVAAL
LLHPGIDVTVLNNNGNPANWALPKHLAKTLNWNEVSMLTVKADPQDAGSILNLHMDAKDEV
TDSSRKDIKTLTQTYTGNTSLVAILIATITFAAAFTLPGGYSTDAGNEGLPIMARKIAFKAFLISDAL
AMCSSLAVAFICVIARWEDLEFLLYYRSFSKKLMWFAYMATTTAFATGLYTVLAPQLPGLAVAIC

VLTSLLPILTKLLGEWPILRLRFRLGQTYNSELLKML
>SbANK154
MEVDRRKNLQIGSKGKEEVQSKNQRREEPLASSEVDMDEPEAAESGDMVVDGNNGSSKESPVPP
MDSKNSKGCAAKKRRSVSADFGAELDLDLGNGEESGAQQEERKLSRQDRVELCRSFQHAVSSH
DWESAEGLVGMADAQGLNDVLCVAVDAIWFLSDKDELLAIVGLIRRIVSEGAKDFTRAALRTSFL
ASCVSACRGRSTSLADAVSFMGQKLHERLQESQGDEVLKAEASAKVHRFTEWALKCIGLHSRVR
ENRGKGNHDTVIEVQLQLSAFKTFLDLADNELTGKDFTEAFDAACFPLTLFSTTFDQGWASGISA
AAVQGLLELLVEGGADNVNQCFLEAARYGSTELVRILLQIAQRNSLDIDVDLALGFAAHYGKIET
MGCLVEEGNAVGFLGPLMRAAERGCLQVVEWFVNHGCREMELCLALTAATSSSQIAVAAYLLP
LVPQHVLAPLSIEIIKAAGERSTGSLHGVDFLLRSDFLNDPAATYAVADSIARCTDEAVDAKLRSF
MNEHWSEAAFSAGFESAQQHFVNFMRIMERGESPIRLGDLPLELVIAMAYLPLYKECMNSIGRLL
PQRLRGQLVEAASRLEDRQVERDSQSRELLAILEHHIPRFMTQT
>SbANK155
MRSLISNCFTSLGSGFKAKGADVNGKVFRGYPTTAAAREGCAEVAELLVRAGASQPACEEAVVE
AALQGQAALAAIFMRSDLVRPRVAVHALVSAAARGFVDVVDTLIKCGADPNATARVLLRSLKPS
LHANVDCTALFAAIVSRQVAVVRHLLQAGVKRDTKVRLGAWSWDASTGEELRVGAGLAEPYD
AVWCAVEYYESTGSILRMLLRSGYTSATAHLGRTLLHHAILCGSAGAVQTLLASGADSEAPVKTS
RSNRSRPVHLAVRLGQPEILRMLVDRGCDVNARADAGDTAAILCSRHKREDCLGVLVSAGADV
ALLNSAGDSPSSVASSGGWKTGFERAVIGAIRSGTIPRSSDRNVFSPLMFGALCGDATAMEVLLAQ
SDVDVDEQDLDGCSPIMAAAKMGNVEAFRALVFAGANVKLSNKRGETAIGLAQQSKKRDLFEQ
VMLEFALEKGMPGGFYALHCASRRGDTAAVRHLASTGGDVNIPDGDGYTPLMLAAREGHAGV
CELLISYGARCDLRTPRGETALSLARAALATAGFNKAEDVIMDELGRQAVLQGAHVRKHTKGGR
GRPHGKSLRMVAAAGVLRWGGSNRRNVICREAEVGGSSAFQRHRQRKGDAYEHGLFRVVTAT
GREVHFVCQGGEEAAELWVRGIRAVTRAAFGKRGKE
>SbANK156
MRSDLVRPRVAVHALVSAAARGFVDVVDTLIKCGADPNATARVLLRSLKPSLHANVDCTALFA
AIVSRQVAVVRHLLQAGVKRDTKVRLGAWSWDASTGEELRVGAGLAEPYDAVWCAVEYYESTG
SILRMLLRSGYTSATAHLGRTLLHHAILCGSAGAVQTLLASGADSEAPVKTSRSNRSRPVHLAVRL
GQPEILRMLVDRGCDVNARADAGDTAAILCSRHKREDCLGVLVSAGADVALLNSAGDSPSSVAS
SGGWKTGFERAVIGAIRSGTIPRSSDRNVFSPLMFGALCGDATAMEVLLAQSDVDVDEQDLDGCS
PIMAAAKMGNVEAFRALVFAGANVKLSNKRGETAIGLAQQSKKRDLFEQVMLEFALEKGMPGG
FYALHCASRRGDTAAVRHLASTGGDVNIPDGDGYTPLMLAAREGHAGVCELLISYGARCDLRTP
RGETALSLARAALATAGFNKAEDVIMDELGRQAVLQGAHVRKHTKGGRGRPHGKSLRMVAAA
GVLRWGGSNRRNVICREAEVGGSSAFQRHRQRKGDAYEHGLFRVVTATGREVHFVCQGGEEAA
ELWVRGIRAVTRAAFGKRGKE
>SbANK157
MAVLLRPAAIAGGRQVWPVAEDDEGAREAEAASQRLVEAVARGDAREAGELLAAGRADVNY
AGVVWLRARRIAEAEPQEGAAAEARAVHEEIRADVSPLFLASGNGDVALVRALLAKGADVNGK
VFRGYPTTAAAREGCAEVAELLVRAGASQPACEEAVVEAALQGQAALAAIFMRSDLVRPRVAV
HALVSAAARGFVDVVDTLIKCGADPNATARVLLRSLKPSLHANVDCTALFAAIVSRQVAVVRHL
LQAGVKRDTKVRLGAWSWDASTGEELRVGAGLAEPYDAVWCAVEYYESTGSILRMLLRSGYTSA
TAHLGRTLLHHAILCGSAGAVQTLLASGADSEAPVKTSRSNRSRPVHLAVRLGQPEILRMLVDRG
CDVNARADAGDTAAILCSRHKREDCLGVLVSAGADVALLNSAGDSPSSVASSGGWKTGFERAVI
GAIRSGTIPRSSDRNVFSPLMFGALCGDATAMEVLLAQSDVDVDEQDLDGCSPIMAAAKMGNVE

AFRALVFAGANVKLSNKRGETAIGLAQQSKKRDLFEQVMLEFALEKGMPGGFYALHCASRRGD
TAAVRHLASTGGDVNIPDGDGYTPLMLAAREGHAGVCELLISYGARCDLRTPRGETALSLARAA
LATAGFNKAEDVIMDELGRQAVLQGAHVRKHTKGGRGRPHGKSLRMVAAAGVLRWGGSNRR
NVICREAEVGGSSAFQRHRQRKGDAYEHGLFRVVTATGREVHFVCQGGEEAAELWVRGIRAVT
RAAFGKRGKE

>SbANK158

MAGDWQELGQAAAIGLLFAYLVAKLISTVIAFKEDNLRITRSPPSSPTAAAPAPATPFPAEPRLPS
HDGSTDGGSASDSDWEGVESTELDEEFSAASAFVAASAASGTSVPEEAQLRLYGLYKIATEGPCTA
PQPSALKLKARAKWNAWHKLGAMPTEEAMQEYITIVQELFPNWDAGASAKRKDEDNMASASA
SKGPMGPVFSSLMYEEDEGNDSELGDIHVSAREGATEDIVKFLAAGVEVNVRDTEGRTPLHWAV
DRGHLSAVEVLAKANADLNAKDNEGQTALHYAAVCEREDIAELLVKHHADLQIKDEDGNTAQ
DLCPSSWSFMNQAN

>SbANK159

MAAAAPEKRPPRSLFELASDFFDSSVLLRAHPSTAPSAVEPSEPSRSPPPPTTQQQQQQLSEASGSR
WTCNTCAAEFESLQEQREHFKSDLHRLNVKLSIAGKTIIKEEDLDKADSDSLFDDLEISSVSGSEDE
QENGPASERLLSVKGKEEFRKKLYFRRASGDTISIYRCMLLKEHEEPLIDCKSGQMENASCVPEEE
MINRVKHLTCEPRDASHLRIVILTSGGHFAGCVFDGNSIVAHKTFHRYVVRAKAGKRQSGKDAT
GKVAHSAGSSLRRYNEAALKKEVQELIVSWKSYFETCVCAFIYAPSKNRQMLFDGEKTQSVIQSC
DIRPVPLTVHRPTLKEAKRVYCNLTQLYYEMECSTTDETLPDAGSVKNVEQSQGKKKEVDADPEE
SISDLSARLELLNKNESATIPSSKNETTPLHEAAKCGNAQLTLELLEQGLDPCIKDARGKTPYSLAS
DKEVRNTFRRFMALNLDKWDWHAADVPSALTKEMEESQAAKQADKDAKRKARAKELKKLKK
AREKEEKEKEKAKAQASQSQTDVKGTSSGQMANRTASVPGLKPKHQTPQQILIAKEEERQRKQA
EEREKRAAAAERRLAALAAQSAGAGASGAAATNCSAQRAVPDDNSCSCCFASLAGKVPFHRYN
YKYCSTTCMHLHSEMLQDD

>SbANK160

MIWRYLVSLVQRTNKKMDLHQNVCSQLKMENASCVPEEEMINRVKHLTCEPRDASHLRIVILTS
GGHFAGCVFDGNSIVAHKTFHRYVVRAKAGKRQSGKDATGKVAHSAGSSLRRYNEAALKKEV
QELIVSWKSYFETCVCAFIYAPSKNRQMLFDGEKTQSVIQSCDIRPVPLTVHRPTLKEAKRVYCNL
TQLYYEMECSTTDETLPDAGSVKNVEQSQGKKKEVDADPEESISDLSARLELLNKNESATIPSSKN
ETTPLHEAAKCGNAQLTLELLEQGLDPCIKDARGKTPYSLASDKEVRNTFRRFMALNLDKWDW
HAADVPSALTKEMEESQAAKQADKDAKRKARAKELKKLKKAREKEEKEKEKAKAQASQSQTD
VKGTSSGQMANRTASVPGLKPKHQTPQQILIAKEEERQRKQAEEREKRAAAAERRLAALAAQSA
GAGASGAAATNCSAQRAVPDDNSCSCCFASLAGKVPFHRYNYKYCSTTCMHLHSEMLQDD

>SbANK161

MEREMSAEYELNEIDAADTLHGSVGSRLSLFARELKSRRSSWQSSSALRLPQNCFYGSFVIHPNGR
WYRIWSTAMFLWSIYSTFFTPFEFGFFRGLPEHLLDLECVQLVFLADVAVHFFLAYRDAHTYRMV
YDKRKIALRYIKGSFALDILGCFPWDFIYKATGRTEVVRCLVWLRLYRARNILAFFKRMEKDIRISY
LFTRIVKLITVELHFTHTAACVFYYLATTLPPAREGGTWIGSLTLGGTRYISFREMDLLTRYVTSLYL
AIVTMATVGYGDVHAVNPREMVFTVVYISFSILLSAYLIGNMTALIVKGSKTERFRDKMTDLIRYM
NRNKLGAGIRSQVKDHLLLQYESSYTRDRVVDDIPVAVRSKMSQTLYLDMVSRVHLFRGCSEDFL
SQIVVKLHEEFFLPGEVILEQGTVVDQIYIVVHGCLEEVATGEDGSEEIISELLPYDIIGDVSVVCNVP
QPHTVRVCELCSLLRIDKQSLTSILQIYFKDSRQILSNLLKGRATESKGKQLESDITYLISRQEAELVL
GVNNAAYHGDLFRLKGLISAGADPSKPDHDGRTALHVAALRGYEDIVRFLIQRGANVNSIDKFG
NSPLLLALKSGHERITSLLVKHGATLNLEDAGGYLCRVVTDGKVDLLKRLLRFGVDPNCKNYDQ

RTPLHVAAAEGLHLVATMLVDFGADVLAKDRWGNTPLDEGRRCSSRPLVRILEQARTVAVTL
>SbANK162
MSQSFDINVLREEARSRWLKPSEVYYILQNHERFPITHEAPKKPPSGSLFLYNRRVNRYFRRDGHT
WRRKKDGRTVGEAHERLKVGNVDSLSCYYAHGEQNPCFQRRCFWMLEPAYEHIVLVQYREVAE
GRYYSSQLSNGPAEPLSSLGYPNAICGNQYYSSISGTSEGSESHQSYSNLSSVTEVSSYSGNKEYHKN
DGSLLSIPEVGHTCLQQNRTDNGNSKNKSGLNMALKKIAEQLSLGEDDDDYIYSNQAHSVGGD
NQVKQIQQEGTQKVLSRNIAPSWEDVLHSSSGLPTSSIYQQSDVEYQKNSEYHPPECLDSSDLRIQL
SAAKRFLLGPEATIDSPSLNSALRNRVNSVTDTISAYDSRFESSLNPDWQTKTPSTFQSNSQGSEITE
LFDHGHFEPYSREDTAFALQQTNKFNIREISPEWAFSYEITKVIITGDFLCDPSNLCWAVMFGDSEV
PAEIVQPGVLRCHTPLHSSGNLRICITSGNREVCSEFKEFEFRSKPTSSSFTDIAPSSKYLKSSEELLLL
AKFARMLLSGNGSPEVPDGDPQSGQCPKLKMNEELWDRLIDELKVGCENPLSSVDWILEELLKSK
LQQWLSAKLRGFNGTDFLSKHEQGIIHLISALGYEWALSSVLSAGVGLNFRDSNGWTALHWAAY
FGREKMVAALLAAGASATAVTDPTAQDPVGKTAAFLASERGHTGLAGYLSEVSLTSYLASLTIEE
SDVSKGSAVVEAERAVESISQRSAQLHGGTEDELSMKDSLAAVRNAAQAAARIQNAFRAFSFRK
RQQKTARLRDEYGMTQEDIEELAAASRLYHQSHASSGQFYDKAAVSIQKKYKGWKGRKHFLNM
RRNAVKIQAHVRGHQVRKKYRTIVSTVSVLEKVILRWRRKGHGLRGFRAEQQPMVEAIEEDDEE
DDDFDDDEAVKVFRRQKVDQAVKEAVSRVLSMVDSTEARMQYRRMLEQFRQATAELEGSNEVT
SIFDSDLELLGINNF
>SbANK163
MSCLLNSQNSSGETSGAGTRASMERVKHENFCRAVAEDNTALLLLAVGNSRKEALCRIRKGSDA
SRVLDPEMSARLLHLACKHDAVECARLLLEGGSGIAAAPIDARDQLTWTPLHVAAETHSTRCIEL
LLSKNARTDLRVVDGRPLLPLEIALMSRRVQTKWSVDNSIEDLLSFLKQMDLNAVRLLAEETRVV
GELAYRYAMEGSVPALAMLLLVVEEKISPQVSVVIQGVSTKRSIYNSIVDEALSMGDASARDGNER
RKALLCEIQLLNQFGSASWRDHNDRRTLPPLLRAAKVGDMNVIKMLLMGNVDVNEADSEGNT
ALHWCLSGASYTKEPRIVWLLLKNGARVFQWNKLGLTPVHSAAAKGNYKALQSLLLHAQDCV
DIPTKTKETPLFFAVNNGFVDCAKLLLCFGADTKARNLRKQRPIDVATSQDMRFILSSANVLPWN
HSSPQQNRVMRKKIYKELLGDKFDDHDKDYCDDNYTGFKTSVGQYDFRSSNHSAQGLKSKSQY
VPKQAQGSKFVPRAKASSSGASTATAAKVPATPGHPAAPVVPATSVARTPTQAPVATAETAPPS
AQPQAAPAATLAASGDADVYSQAASNLVSGNNLEQIIQQILDMGGGTWERDTVVRALRAAYN
NPERAIDYLYSGIPVNVETPPVAGAPAGGQQTNQQAPSPAQPAVASPVQPSAASARPDANPLILF
PQVNLHFCGRKIRTSLLPGLSIVKLVQLALLENNIRTTDFYLLLNGRLLDEHVTVNDIPAESNIQVR
PRVRGGVDEVHETTYRSIDTVINKRGHEKMFRRVIIPKQLGKRYACLLSPFSQYILFGILRCVCREA
HQFGKCYNGAFTLADFMISKKGHIKMSSSVFLDNYTPQGGEADYRQLYRILLVFVDAERGLVPLY
FAHLLDTLRSTNELNRRDPDSITFLLNHPSLLTYAERHRLFCLIDQMILKLPREEKEKVFKKVNSIP
GYSKWDSNFEVMKEFNRVYNFPSGKGIHRYAGSGLNTTIHFCRNYLSHPEDWVSILFAEVAISLLV
EDMLAKVIKELLDYQPPVYVGTWDLIGLIANVRADTGTMLNQDGAAYSD
>SbANK164
MHFSDSILACGNPRIACGFCSQIPLWPIFFLASCLQTAWGNSSGETSGAGTRASMERVKHENFCR
AVAEDNTALLLLAVGNSRKEALCRIRKGSDASRVLDPEMSARLLHLACKHDAVECARLLLEGGS
GIAAAPIDARDQLTWTPLHVAAETHSTRCIELLLSKNARTDLRVVDGRPLLPLEIALMSRRVQTK
WSVDNSIEDLLSFLKQMDLNAVRLLAEETRVVGELAYRYAMEGSVPALAMLLLVVEEKISPQVSV
VIQGVSTKRSIYNSIVDEALSMGDASARDGNERRKALLCEIQLLNQFGSASWRDHNDRRTLPPLL
RAAKVGDMNVIKMLLMGNVDVNEADSEGNTALHWCLSGASYTKEPRIVWLLLKNGARVFQW
NKLGLTPVHSAAAKGNYKALQSLLLHAQDCVDIPTKTKETPLFFAVNNGFVDCAKLLLCFGADT

KARNLRKQRPIDVATSQDMRFILSSANVLPWNHSSPQQNRVMRKKIYKELLGDKFDDHDKDYC
DDNYTGFKTSVGQYDFRSSNHSAQGLKSKSQYVPKQAQGSKFVPRAKASSSGASTATAAKVPAT
PGHPAAPVVPATSVARTPTQAPVATAETAPPSAQPQAAPAATLAASGDADVYSQAASNLVSGN
NLEQIIQQILDMGGGTWERDTVVRALRAAYNNPERAIDYLYSGIPVNVETPPVAGAPAGGQQTN
QQAPSPAQPAVASPVQPSAASARPDANPLILFPQVNLHFCGRKIRTSLLPGLSIVKLVQLALLENN
IRTTDFYLLLNGRLLDEHVTVNDIPAESNIQVRPRVRGGVDEVHETTYRSIDTVINKRGHEKMFRR
VIIPKQLGKRYACLLSPFSQYILFGILRCVCREAHQFGKCYNGAFTLADFMISKKGHIKMSSSVFLD
NYTPQGGEADYRQLYRILLVFVDAERGLVPLYFAHLLDTLRSTNELNRRDPDSITFLLNHPSLLTY
AERHRLFCLIDQMILKLPREEKEKVFKKVNSIPGYSKWDSNFEVMKEFNRVYNFPSGKGIHRYAGS
GLNTTIHFCRNYLSHPEDWVSILFAEVAISLLVEDMLAKVIKELLDYQPPVYVGTWDLIGLIANVR
ADTGTMLNQDGAAYSD
>SbANK165
MLNSSGETSGAGTRASMERVKHENFCRAVAEDNTALLLLAVGNSRKEALCRIRKGSDASRVLDP
EMSARLLHLACKHDAVECARLLLEGGSGIAAAPIDARDQLTWTPLHVAAETHSTRCIELLLSKN
ARTDLRVVDGRPLLPLEIALMSRRVQTKWSVDNSIEDLLSFLKQMDLNAVRLLAEETRVVGELAY
RYAMEGSVPALAMLLLVVEEKISPQVSVVIQGVSTKRSIYNSIVDEALSMGDASARDGNERRKALL
CEIQLLNQFGSASWRDHNDRRTLPPLLRAAKVGDMNVIKMLLMGNVDVNEADSEGNTALHWC
LSGASYTKEPRIVWLLLKNGARVFQWNKLGLTPVHSAAAKGNYKALQSLLLHAQDCVDIPTKTK
ETPLFFAVNNGFVDCAKLLLCFGADTKARNLRKQRPIDVATSQDMRFILSSANVLPWNHSSPQQ
NRVMRKKIYKELLGDKFDDHDKDYCDDNYTGFKTSVGQYDFRSSNHSAQGLKSKSQYVPKQAQ
GSKFVPRAKASSSGASTATAAKVPATPGHPAAPVVPATSVARTPTQAPVATAETAPPSAQPQAA
PAATLAASGDADVYSQAASNLVSGNNLEQIIQQILDMGGGTWERDTVVRALRAAYNNPERAID
YLYSGIPVNVETPPVAGAPAGGQQTNQQAPSPAQPAVASPVQPSAASARPDANPLILFPQVNLH
FCGRKIRTSLLPGLSIVKLVQLALLENNIRTTDFYLLLNGRLLDEHVTVNDIPAESNIQVRPRVRGG
VDEVHETTYRSIDTVINKRGHEKMFRRVIIPKQLGKRYACLLSPFSQYILFGILRCVCREAHQFGKC
YNGAFTLADFMISKKGHIKMSSSVFLDNYTPQGGEADYRQLYRILLVFVDAERGLVPLYFAHLLD
TLRSTNELNRRDPDSITFLLNHPSLLTYAERHRLFCLIDQMILKLPREEKEKVFKKVNSIPGYSKWD
SNFEVMKEFNRVYNFPSGKGIHRYAGSGLNTTIHFCRNYLSHPEDWVSILFAEVAISLLVEDMLAK
VIKELLDYQPPVYVGTWDLIGLIANVRADTGTMLNQDGAAYSD
>SbANK166
MGENSSGETSGAGTRASMERVKHENFCRAVAEDNTALLLLAVGNSRKEALCRIRKGSDASRVLD
PEMSARLLHLACKHDAVECARLLLEGGSGIAAAPIDARDQLTWTPLHVAAETHSTRCIELLLSKN
ARTDLRVVDGRPLLPLEIALMSRRVQTKWSVDNSIEDLLSFLKQMDLNAVRLLAEETRVVGELAY
RYAMEGSVPALAMLLLVVEEKISPQVSVVIQGVSTKRSIYNSIVDEALSMGDASARDGNERRKALL
CEIQLLNQFGSASWRDHNDRRTLPPLLRAAKVGDMNVIKMLLMGNVDVNEADSEGNTALHWC
LSGASYTKEPRIVWLLLKNGARVFQWNKLGLTPVHSAAAKGNYKALQSLLLHAQDCVDIPTKTK
ETPLFFAVNNGFVDCAKLLLCFGADTKARNLRKQRPIDVATSQDMRFILSSANVLPWNHSSPQQ
NRVMRKKIYKELLGDKFDDHDKDYCDDNYTGFKTSVGQYDFRSSNHSAQGLKSKSQYVPKQAQ
GSKFVPRAKASSSGASTATAAKVPATPGHPAAPVVPATSVARTPTQAPVATAETAPPSAQPQAA
PAATLAASGDADVYSQAASNLVSGNNLEQIIQQILDMGGGTWERDTVVRALRAAYNNPERAID
YLYSGIPVNVETPPVAGAPAGGQQTNQQAPSPAQPAVASPVQPSAASARPDANPLILFPQVNLH
FCGRKIRTSLLPGLSIVKLVQLALLENNIRTTDFYLLLNGRLLDEHVTVNDIPAESNIQVRPRVRGG
VDEVHETTYRSIDTVINKRGHEKMFRRVIIPKQLGKRYACLLSPFSQYILFGILRCVCREAHQFGKC
YNGAFTLADFMISKKGHIKMSSSVFLDNYTPQGGEADYRQLYRILLVFVDAERGLVPLYFAHLLD

TLRSTNELNRRDPDSITFLLNHPSLLTYAERHRLFCLIDQMILKLPREEKEKVFKKVNSIPGYSKWD
SNFEVMKEFNRVYNFPSGKGIHRYAGSGLNTTIHFCRNYLSHPEDWVSILFAEVAISLLVEDMLAK
VIKELLDYQPPVYVGTWDLIGLIANVRADTGTMLNQDGAAYSD
>SbANK167
MLNSSGETSGAGTRASMERVKHENFCRAVAEDNTALLLLAVGNSRKEALCRIRKGSDASRVLDP
EMSARLLHLACKHDAVECARLLLEGGSGIAAAPIDARDQLTWTPLHVAAETHSTRCIELLLSKN
ARTDLRVVDGRPLLPLEIALMSRRVQTKWSVDNSIEDLLSFLKQMDLNAVRLLAEETRVVGELAY
RYAMEGSVPALAMLLLVVEEKISPQVSVVIQGVSTKRSIYNSIVDEALSMGDASARDGNERRKALL
CEIQLLNQFGSASWRDHNDRRTLPPLLRAAKVGDMNVIKMLLMGNVDVNEADSEGNTALHWC
LSGASYTKEPRIVWLLLKNGARVFQWNKLGLTPVHSAAAKGNYKALQSLLLHAQDCVDIPTKTK
ETPLFFAVNNGFVDCAKLLLCFGADTKARNLRKQRPIDVATSQDMRFILSSANVLPWNHSSPQQ
NRVMRKKIYKELLGDKFDDHDKDYCDDNYTGFKTSVGQYDFRSSNHSAQGLKSKSQYVPKQAQ
GSKFVPRGHPAAPVVPATSVARTPTQAPVATAETAPPSAQPQAAPAATLAASGDADVYSQAAS
NLVSGNNLEQIIQQILDMGGGTWERDTVVRALRAAYNNPERAIDYLYSGIPVNVETPPVAGAPA
GGQQTNQQAPSPAQPAVASPVQPSAASARPDANPLILFPQVNLHFCGRKIRTSLLPGLSIVKLVQL
ALLENNIRTTDFYLLLNGRLLDEHVTVNDIPAESNIQVRPRVRGGVDEVHETTYRSIDTVINKRGH
EKMFRRVIIPKQLGKRYACLLSPFSQYILFGILRCVCREAHQFGKCYNGAFTLADFMISKKGHIKMS
SSVFLDNYTPQGGEADYRQLYRILLVFVDAERGLVPLYFAHLLDTLRSTNELNRRDPDSITFLLNH
PSLLTYAERHRLFCLIDQMILKLPREEKEKVFKKVNSIPGYSKWDSNFEVMKEFNRVYNFPSGKGI
HRYAGSGLNTTIHFCRNYLSHPEDWVSILFAEVAISLLVEDMLAKVIKELLDYQPPVYVGTWDLIG
LIANVRADTGTMLNQDGAAYSD
>SbANK168
MSCLLNSQNSSGETSGAGTRASMERVKHENFCRAVAEDNTALLLLAVGNSRKEALCRIRKGSDA
SRVLDPEMSARLLHLACKHDAVECARLLLEGGSGIAAAPIDARDQLTWTPLHVAAETHSTRCIEL
LLSKNARTDLRVVDGRPLLPLEIALMSRRVQTKWSVDNSIEDLLSFLKQMDLNAVRLLAEETRVV
GELAYRYAMEGSVPALAMLLLVVEEKISPQVSVVIQGVSTKRSIYNSIVDEALSMGDASARDGNER
RKALLCEIQLLNQFGSASWRDHNDRRTLPPLLRAAKVGDMNVIKMLLMGNVDVNEADSEGNT
ALHWCLSGASYTKEPRIVWLLLKNGARVFQWNKLGLTPVHSAAAKGNYKALQSLLLHAQDCV
DIPTKTKETPLFFAVNNGFVDCAKLLLCFGADTKARNLRKQRPIDVATSQDMRFILSSANVLPWN
HSSPQQNRVMRKKIYKELLGDKFDDHDKDYCDDNYTGFKTSVGQYDFRSSNHSAQGLKSKSQY
VPKQAQGSKFVPRGHPAAPVVPATSVARTPTQAPVATAETAPPSAQPQAAPAATLAASGDADV
YSQAASNLVSGNNLEQIIQQILDMGGGTWERDTVVRALRAAYNNPERAIDYLYSGIPVNVETPPV
AGAPAGGQQTNQQAPSPAQPAVASPVQPSAASARPDANPLILFPQVNLHFCGRKIRTSLLPGLSI
VKLVQLALLENNIRTTDFYLLLNGRLLDEHVTVNDIPAESNIQVRPRVRGGVDEVHETTYRSIDTV
INKRGHEKMFRRVIIPKQLGKRYACLLSPFSQYILFGILRCVCREAHQFGKCYNGAFTLADFMISKK
GHIKMSSSVFLDNYTPQGGEADYRQLYRILLVFVDAERGLVPLYFAHLLDTLRSTNELNRRDPDSI
TFLLNHPSLLTYAERHRLFCLIDQMILKLPREEKEKVFKKVNSIPGYSKWDSNFEVMKEFNRVYNF
PSGKGIHRYAGSGLNTTIHFCRNYLSHPEDWVSILFAEVAISLLVEDMLAKVIKELLDYQPPVYVG
TWDLIGLIANVRADTGTMLNQDGAAYSD
>SbANK169
MGENSSGETSGAGTRASMERVKHENFCRAVAEDNTALLLLAVGNSRKEALCRIRKGSDASRVLD
PEMSARLLHLACKHDAVECARLLLEGGSGIAAAPIDARDQLTWTPLHVAAETHSTRCIELLLSKN
ARTDLRVVDGRPLLPLEIALMSRRVQTKWSVDNSIEDLLSFLKQMDLNAVRLLAEETRVVGELAY
RYAMEGSVPALAMLLLVVEEKISPQVSVVIQGVSTKRSIYNSIVDEALSMGDASARDGNERRKALL

CEIQLLNQFGSASWRDHNDRRTLPPLLRAAKVGDMNVIKMLLMGNVDVNEADSEGNTALHWC
LSGASYTKEPRIVWLLLKNGARVFQWNKLGLTPVHSAAAKGNYKALQSLLLHAQDCVDIPTKTK
ETPLFFAVNNGFVDCAKLLLCFGADTKARNLRKQRPIDVATSQDMRFILSSANVLPWNHSSPQQ
NRVMRKKIYKELLGDKFDDHDKDYCDDNYTGFKTSVGQYDFRSSNHSAQGLKSKSQYVPKQAQ
GSKFVPRGHPAAPVVPATSVARTPTQAPVATAETAPPSAQPQAAPAATLAASGDADVYSQAAS
NLVSGNNLEQIIQQILDMGGGTWERDTVVRALRAAYNNPERAIDYLYSGIPVNVETPPVAGAPA
GGQQTNQQAPSPAQPAVASPVQPSAASARPDANPLILFPQVNLHFCGRKIRTSLLPGLSIVKLVQL
ALLENNIRTTDFYLLLNGRLLDEHVTVNDIPAESNIQVRPRVRGGVDEVHETTYRSIDTVINKRGH
EKMFRRVIIPKQLGKRYACLLSPFSQYILFGILRCVCREAHQFGKCYNGAFTLADFMISKKGHIKMS
SSVFLDNYTPQGGEADYRQLYRILLVFVDAERGLVPLYFAHLLDTLRSTNELNRRDPDSITFLLNH
PSLLTYAERHRLFCLIDQMILKLPREEKEKVFKKVNSIPGYSKWDSNFEVMKEFNRVYNFPSGKGI
HRYAGSGLNTTIHFCRNYLSHPEDWVSILFAEVAISLLVEDMLAKVIKELLDYQPPVYVGTWDLIG
LIANVRADTGTMLNQDGAAYSD
>SbANK170
MGLLGMMGDSFGCSATGERLVSAARDGDIQEARALLELNPRLARYSTFGIRNSPLHYSAAKGHH
EIVSLLIESGVNINLRNCRGQTALMQACLYGHWKVVQILVLFKANIHRRDCFSGATAIHFAALKG
HTRCIRLLVADYVPSLSEFWTIMRAKSKDQMKKDAFDAVALQRLVNGKSDGGVTPLHLAALHG
HAESVQLLLDLGASVSEVTVNDGSTIDLIGSGSTPLHYAACGGSAVCCQLLIAAGANMGAQNAN
GLTPLLVARSWHKTSIEGILSKQPENRMRILPSPYLCLPLMSIVKIARECGWRKTSASSTCQDPCVIC
LEVECTVAAEGCGHEFCTKCALYLCSTTSSSTSIRGVPGSISCPLCRHAIVSFMRLTSTTPIKELPWTS
ASLALCAAGASTGPDHGRSLQRRSDMRRLRSSSVQLGCSSFISIGSGKLSSIKLNCTGAEEAVPCLV
SCLRPDVQRSSSYRERIRRYSEF
>SbANK171
MASSGDPPGSTDEKKTPKSEGSSDERQGVPPAGFSNPFDFASMQSLLNDPSIKEMADQIAKDPAF
NQMAEQLQKSAHSTGEQGMPPLDPQQYMETMQKVMENPQFMTMAERLGNALMQDPAMSSM
LETFSSPSHKEQLEERMSRIKEDPAMKSILDELENGGPSAMMKYWNDPDTLQKIGEAMGASFPFG
AGSSAEPSGPEETEEEGGDDDESIVHHTASVGDEEGLKKALDGGADKDEEDSEGRRALHFACGY
GELKCAQILLEAGAAVDALDKNKNTPLHYAAGYGRKECVDLLLKHGAAVTLQNLDGKTPIDVA
KLNNQDEVLKLLEKDVFL
>SbANK172
MEDSIVMPGSAAAAAPPPAERPCNGAADQGQQQLQVEEQPGDDVEAGLRQAPGGDVLIPRGQ
AGVETEIQNAGEEDLETLLHVAVRKRNDNPAEFEILRRFAKSPDINGMTPLYLAIFLGYGDMAEL
LIRTFGRDLSYDGPNGQNVLHVAALRSRDVTQKILEAQKVLLSSNEVSWEDLRNVADASGSTPLH
FAASVGVKGVTSLLVDGDTTGMRKKAVDSNGMCPIHIAASVGAMDAVHALVGEEDTHAALRE
NRMGRTFLHIAVENKKTDVVKLVCRKSRPAFRNILNMKDKDGNTALHLAVQNRDESSFSHLVG
NRYVDLNHVNKDGYTPLDLASKIKIENSFASPKNPTEWMVRVLAHSGAYFGAHRRDMKYGTIT
QKDQRADHTAPSTETRTQKKKKKKQAAELDESVLVASALIATVTFAAAFTMPGSYKTEGHRAGT
PALASRYGFKVFVIADILAFYCSVAATFSLAEYGNRGDVDPLVRRVYAQRAVRFFHVALKSVIIAF
ALGVGVVMWDVSVVTVVIVAVATSALVLYGNEALAHDLRFMLVMYRRFGFSRAWGLCPSTSSH
LHWNSWRLRSFFATLAWNIAKLFGTYILIFLVAYIAQLKQKSGI
>SbANK173
MERQSSIRLGALEKLKSFRGMEKQKSFRGIMSLERRSRDSPGKRGDTPLHLAARSGSVAHAQRILA
ELDRALVAEMAAKQNQDGETPLYVAAEKGHAEVVREILKVSDVQTAGIKASNSFDAFHIAAKQ
GHLEVLKEMLQALPALAMTTNSVNATALDTAAILGHVDIVNLLLETDASLARIARNNGKTVLHS

AARMGHVEVVRSLLNKDPGIGLRTDKKGQTALHMASKGQNAEIVVELLKPDVSVIHIEDNKGN
RPLHVATRKGNIIIVQTLLSVEGIDVNAVNRSGETAFAIAEKMNNEELVNILKEAGGETAKQQVH
PPNSAKQLKQTVSDIRHDVQSQIKQTRQTKMQVNQIKKRLEKLHIGGLNNAINSNTVVAVLIATV
AFAAIFTVPGNFVEDLTQAPPGMSLGQAYVASNPAFIIFLVFDALALFISLAVVVVQTSLIVVERRA
KKRMVFVMNKLMWLACLFISVAFIALTYVVVGRDDWWLAWCTMAIGTVIMLTTLGSMCYCIIA
HRLEEKNTRKIRKKASASQSRGSWSRSVDSDEEILNSEYKTKMYAL
>SbANK174
MAAAAMHPYTRQPYVRPSHTTTMHPRLLMAARLGDTQRLKNLLDDGRSVSVGAHVVRVEGSS
SSSGPSAATPLTTTACGSSCSLLHVVAASGDGDEFLESAKVIHDRARHLLGIPDSNGDTPLHLAAR
AGNARMVSHLIHLAKTTDDDVAGEEGHGGADESRSSRLVKELLRGENRRGETVLHDAVRVGSR
CMVIRLMEEDPELASFPREEGRGASPLYLAVVMEEVAIARSLHDMSHGSLSYAGPNGQNALHAA
VLRGKVMTAMLLGWNEGLTEQGDHDGCTPLHFATSQQPEEGRSLPCRISNKFPWVRLSAADIPL
LLLQTNPCSAYCRDAGGAFPIHVAAAVGAHKAVTTLLGMSPDSAGLQDAGGRTFLHVAVEKKR
HSVVKHACRAPSLAWILNMQDKDGNTALHLAVKAGDTRTFFLLFGNRQVRMDLANNDGQTC
RDLSLIDIPPGLSYKWNPKQMIHRALTRARAAHGIRRWDQFEEECILRPRREDEEKESEKLNNSTQ
TLGISSVLIVTVTFGATFALPGGYIADDHANGGAPTLAGRRAFRVFVVANALAFICSSLGTVGLMY
SGITTVDLPIRQRHFLRSLFFVSSSLTCLVVAFASGSYTVLSPVAHSTAVAICVISMVVIVYRSLGRFQ
RMYALAAPLYVRAGIQPLLRLAKDIFTRMLRLYWPFIVIFTWAACATNHDA
>SbANK175
MYFSKLDDSPMFRTQIQSLEESAELLRERCLKFHKGCRKYTEGLGEAYDGDIAFASSLETFGGGHN
DPISVAFGGPVMTKFTIALREIGTYKEVLRSQVEHMLNDRLLNFVDIDLHDVKDAHKRFDKASLL
YDQIREKYLSLKKGTRPDITTAIEDELHSARSSFEQARFNLVTSLSHVEAKKRFEFLEAVSATMDSH
LRYFKQGYELLHQMEPYINQVLAYAQQSRERANKEQASLVERMHEYKRQIDRESRSSINGLNDSY
NGDGIQTIGRSSHKQIEAVMQSTSKGKVQTIRQGYLSKRSSNLRADWKRRFFVLDSRGMLYYYRK
QITRPPAGCSMQRSVNPPEHGSGLLSRLFSSHYHGIIHDEKSVARHTVNLLTSTIKVDAEQSDLRFC
FRIISPTKIYTLQAESAVDQMDWIEKITGVIASLLSSQSPEQCFLSSPKGSGHDRSTSDGSSFTSSVEFE
PSTIDDLVLEKYSGNGQHDVRGTHHHRTSVKPEKPIDLLRKVDGNNMCADCGASEPDWASLNL
GALLCIECSGVHRNLGVHISKVRSLTLDVRVWEPSVINLFQSLGNMFVNNIWEEMLPDDNSSADG
SDTSQYLSVSKPKHKDVFSAKEKFIHAKYVNKEFIRNHSMDENQLAQQMWNSVAANDKKAAYS
LIVRSRPNVNLVYGEMPSSPFLTLGKALQQEQPASPHDGSPRFFDCNSHDKISPREPLSPASTSSRT
DDMEDTCEGLSLFHLACRVADVGMVELLLQYGASVNMNDSRGRTPLHHCILKGRHQHAKLLLS
RGADSQAMDRDGRTALQYAIDSGTSDEDILALLEDHSR
>SbANK176
MDSEPDFELRGLDIDVELDPEDLQPSVPLKKVPGGDLFEAARAGDCDRLALLLEAGANVNARDR
WDSVALYYACLAGHAEAARMLLEAGAVCAERTFDGDRCHYAALNLRLRWLLKSFEARPPPLAP
LPAALRATFLACPANRTAFLEMLQGSAGAESAALAAAAGFGPKDDPSSACLFPPDITFYVDRKPV
EAHRVILCARSPFFEKKFKTDWKDRKEVRFSNQKLYYGALYSLIHFFYSDRLEVAVDDMENLARA
CKVCKCEELQKILDKEVVHQKYAEYKSAKELDLDNSQKRFILQAQSLPKEDRLPSALQRILQTCLA
NSREGDYSEESNEMIRNSEDDDLADLYIKVGDKVFHCHQVILASRSEYFRARLSRTVDFLDGNSGF
QAAQNVPLLEEHDLSAEAFEKMLEYMYTDKLEHLDPDQAEELFDVASRYLLFPLKRVVADMLLP
YLEHVSPAELCHWLMLSDIYGVMKIREYILDIIACNFEMFADTREFRALLLTLPPPSGDDSLRTTRP
SAPGTGGNTDQGNILDDLREKWLEAEGAELDERDESAALFDRRLEMLMLVAEKEADDVDA
>SbANK177
MDSEPDFELRGLDIDVELDPEDLQPSVPLKKVPGGDLFEAARAGDCDRLALLLEAGANVNARDR

WDSVALYYACLAGHAEAARMLLEAGAVCAERTFDGDRCHYAALNLRLRWLLKSFEARPPPLAP
LPAALRATFLACPANRTAFLEMLQGSAGAESAALAAAAGFGPKDDPSSACLFPPDITFYVDRKPV
EAHRVILCARSPFFEKKFKTDWKDRKEVRFSNQKLYYGALYSLIHFFYSDRLEVAVDDMENLARA
CKVCKCEELQKILDKEVVHQKYAEYKSAKELDLDNSQKRFILQAQSLPKEDRLPSALQRILQTCLA
NSREGDYSEESNEMIRNSEDDDLADLYIKVGDKVFHCHQVILASRSEYFRARLSRTVDFLDGNSGF
QAAQNVPLLEEHDLSAEAFEKMLEYMYTDKLEHLDPDQAEELFDVASRYGVMKIREYILDIIACN
FEMFADTREFRALLLTLPPPSGDDSLRTTRPSAPGTGGNTDQGNILDDLREKWLEAEGAELDERD
ESAALFDRRLEMLMLVAEKEADDVDA
>SbANK178
MFYHQIAALLLENGADVNARNIYGQTALMQACRFGHWEVVQTLLVFRCNVSKVDSLSSRTALH
LAAAGGHVKCARLLLAGAGGNKLVNRAASGGVTALHLAALHGHADCVHLLIDERADVAAPT
LPCAASPMASIGAGSTPLHYAAAGGEVKCCQILVSRGADRTAVNCNGWLPVDVARTWGCHWL
EHVLSPKSHLPIPKFPPSAYLSSPLASLLTIARDCGLVLNTTPPEVLDDDGGDACAVCLERPCTVAA
EVCGHELCVKCALDLCSVIKSYDVPGIAGTIPCPLCRSGIASFRRRAADEDEPDVNAGGGRRRGA
GGDHQASSSPEKKRSTDSDQEILPLFFTPPAVLS
>SbANK179
MQACRFGHWEVVQTLLVFRCNVSKVDSLSSRTALHLAAAGGHVKCARLLLAGAGGNKLVNRA
ASGGVTALHLAALHGHADCVHLLIDERADVAAPTLPCAASPMASIGAGSTPLHYAAAGGEVKC
CQILVSRGADRTAVNCNGWLPVDVARTWGCHWLEHVLSPKSHLPIPKFPPSAYLSSPLASLLTIA
RDCGLVLNTTPPEVLDDDGGDACAVCLERPCTVAAEVCGHELCVKCALDLCSVIKSYDVPGIAG
TIPCPLCRSGIASFRRRAADEDEPDVNAGGGRRRGAGGDHQASSSPEKKRSTDSDQEILPLFFTPP
AVLS
>SbANK180
MGNALGCTGLGERLAAAARDGDAAEVRRLLEANPGLARCAAFGSLNSPLHLAAAKGHHEIAA
LLLENGADVNARNIYGQTALMQACRFGHWEVVQTLLVFRCNVSKVDSLSSRTALHLAAAGGH
VKCARLLLAGAGGNKLVNRAASGGVTALHLAALHGHADCVHLLIDERADVAAPTLPCAASPM
ASIGAGSTPLHYAAAGGEVKCCQILVSRGADRTAVNCNGWLPVDVARTWGCHWLEHVLSPKS
HLPIPKFPPSAYLSSPLASLLTIARDCGLVLNTTPPEVLDDDGGDACAVCLERPCTVAAEVCGHEL
CVKCALDLCSVIKSYDVPGIAGTIPCPLCRSGIASFRRRAADEDEPDVNAGGGRRRGAGGDHQAS
SSPEKKRSTDSDQEILPLFFTPPAVLS
>SbANK181
MGRGPLGSKRRVAEGETEPEEEEEESAAEYEVEEVRDHIASSRGSRLALFGSDLRLGRFRPRRRTRR
RLLAGEVGAVEGFFHDLVIHPDNKWYRLWTKFILVWAVYSSFFTPLEFGFYRGLPKNLFFLDIAG
QIAFLIDIVVKFFVAYRDPDTYRIVYNPTAIALRYCKSSFIFDLLGCFPWDVIYKACGSKEEVRYLLW
IRLTRVTKITEFFWRLEKDIRVNYLFTRIVKLIVVELYCTHTAACIFYYLATTLPESMEGYTWIGSLQL
GDYKFTHFREIDLVTRYITSLYFAIVTMATVGYGDIHAVNIREMIFIMIYVSFDMILGAYLIGNMTA
LIVKGSRTERFRDKMKEVIRYMNRNKLGKEIREQIKGHLRLQYESSYTEASVLQDIPISIRAKISQTL
YKPYIESIPLFKGCSAEFIQQIVIRLQEEFFLPGEVILEQGSAVDQLYFVCHGALEGVGIGEDGQEETL
LMLEPESSFGEISILCNIPQPYTVRVCELCRLLRLDKQSFTNILEIYFVDGRRILSNLSESEYGGRVKQL
ESDITFHIGKQEAELTLRVNSAAFYGDLNQLKSLIRAGADPKNTDYDGRSPLHLAASRGYEDVVQ
FLINEGVDIDHTDQFGNTPLLEAVKQGHERVAALLFTKGAKLNLKNAGSHLCTAVAKGDSDFIR
RALAYGADPNCRDYDHRTPLHIAAAEGLYLIAKMLVEAGASVFATDRWGTTPLDEARKCGGRT
LGALLEEARANELAMFPERGEEVRDKMHPRRCSVFPYHPWRAAGTGEERRRMEGVVLWIPHTIE
SLVASAQEKLELPGPASRLRLLCEDGARVLDVDMVNDGQKLYLVGGEDGDQKDGQ

>SbANK182
MASEIEVLEDTTTSSTSLVAAAAAAAAPSAAEGAGAPAEDESLKNDVYTAAAYGDLEKLQRLVE
GEGRPVTEPDGGGYHALQWAALNNRVAAAQYILEHGADINAVDHTGQTALHWSAVRGHIQV
AELLLKEGAKVDAADLYGYQATHVAAQYGQTAFIYHIVAKWNADPDIPDNDGRSPLHWAAYK
GFADSIRLLLFLDAYRGRQDKEGCTPLHWAAIRGNLEACTVLVQVGKKDDLMVKDKTGLTPAQ
LAADKNHRQVAFFLDNARRVHDRGCGANTRFGKLSKLGLAPLLWCTIIGMLIIYMHSVISGQYA
TTMTAPFGMFAWSGVFLATAGLVMFYKCSRKDPGYININTRGSQNQRDDEPLLKMELENPALLS
GNWSQLCITCKIVRPVRSKHCSTCDRCVEQFDHHCPWVSNCIGKKNKWEFFMFLTLEVFAMIITG
SAAIIRIVRDPDSPSSFGAWIHYSAFQHPGVVSFLALDCFLFFGVAVLTVVQASQIARNITTNEMAN
SMRYAYLRGPGGRFRNPYDHGIRKNCSDFLFNGYNEDTERLDQALHTDEEMGMIQMTSAVSQN
GDNHLHHANGTDHSCADSQANSKPHSQVGSSQCCDHSKKTDRTPLGLGLGLGRNSASRQYVRS
LIPL
>SbANK183
MADVAGNETDQGAAARKSDDAWVQQGKLDPQLLMAAHCHDSKRLKELLRVNINDEDHADG
GGIGMVGAAPASEAVVIVDVVPRRPPPPSPAASGAPPPPPPPDEGMTMEGDSLLHVVAACGDGD
DDCLDCAKMIVHDKKHKGGGGAAAMRLALEARNSNGDTPLHRAAAAGNARMISCLLDLVAC
TAADDDEAAAIMKALVRTQNKRGETALHQAVRAPAATKVACVDRLMDVDPELACIPFPHQQE
DAASPLYLAISLGELGIARHLHSKSKGNVSYSGPDGRNVLHAAVHRGQAAALPMVLEWLKKDP
KAMEDDDMPRQQEEGQGHADLLSHLTSQRDKENGSTPLHLAASWSGLPSANWFHRTHPYFWG
WSGSPARMLLDANESMAYQPDKRGLYPIHVAAGAGSLRVVKALLGKCPECAVLRDAEGRTFLH
VAVEEGRYGVVKYVCRQNPGLASSSILNAQDKNGDTPLHRAVHAGYSGIFYCLVRNPWVRLDV
QNNKGRRPIDVSWSTMPLRVYYAWDPRIHIQKYLLRVGAPYGESRGDLFGQKHVLRKEDEDKISE
NLTAAVQVMCIFSVLITTVTFASAFTLPGGSRSAGDAGGGVPGTPVLAGSSYVFDAFILSDALAFIF
SLYATSLLLYAGVPYGTLNARFSNINLAYTLLWHAGRSLLAAFALGLYVVLLPVARTIAIAVAVL
MVIIAIGFLKASEGINSMIVIGRIPIPAVWNVVLGWVVYVLDHFWSTIIIFGLPAIRKWGNLP
>SbANK184
MAAVAAGNESELQSAAAVEPPLTTAAAAADARALATLDPQLLMAARRGNSKALKDLLQLDDD
DQQGEDTHGRINDEGSGTPQPTTAAAASTTTSVAVAPSISPPHVIVEVDPRRLDAVAAHLPPPPQ
PVPVPVLDEDGVTTEEGSLLVHSEEIVEQVVVEPPPRPHDDDAAPPPARQPPAAAPAVILDEDGV
TMEGDSLLHVVAACGDTQEFLDCVDVVVRNKEKKSGAGAGGTAKRRALLEARNNKGDTPLHC
AAGAGNAHMITRLVDLMANTADDDEATTVAAAKLAFLRMQNECGETALHQAIRAAAANHKL
INEVACWACIEELMAMDPELACIPHEDGASPLYLAISLGEVGIAQHLYVQSKGKLSYSGPDGRNV
LHAAVYFDRALCKVLEWLEDTKPKEDSSSSVCLVSQLTRQREYNGSTPFHLAASMGWCDVARVL
LDANESTAYQADDQGSYPIHVAAWADNSLGVVKLLLERCPDCATLRDAQGRTFLHVAAEKERL
AVDNNGDTALHSAVRTGNLAVFNCLFRNRQVRLNVANKDGMTPLDLSWTMIPEGFHYGLNPI
NIVHWSLVAAGARYSRGRSSLAFFAEKYMPKREAYTDEESKKYTEATQVMSIVTALIATVTFASAF
TLPGGYRSADGQPVFAGSYAFDAFILADTLAFICSISATCTLVYAGVPAVDSALRHWYFTLSVVLL
QSAARSLVAAFGLGLYLLLAHTATATATAIAAFAIVLASSLYGHMEAWRIIRMAAMVRARIGTPL
RAALIYLGAISLQVLAHFWSYVIIFGLPAIRKRVHQNQLI
>SbANK185
MAAATASGSEQQAAAPEPTAPPAPACPRATLDPQLFMAARRGDSKLLKHLLQLQDEDEDEEDD
ANNAVRKGGLILDEANNGGVRTNEGDLLLQVVDSGEIEVLDDDHRRSSSHGDDDAPPAAVDEH
DEPLDADGVTMEGDSLLHVVAAARGGDDTHKLLDCAKMIVRDKKRRKGGAAAVRLALEARNS
KGDTPLHCAAAAGNDRMITCLVEILPSKYNDDGDEATPVKKKELVRMRNECGETALHHAVRAP

HNNEACIVIDKLMKHDPDLACVLHKDGTSPLYLAISLGKHKIAEHLYSKSQGKLSYSGPHGRNLT
RQRDSEQKGSTPLQLAASLEGWPDARYVYTWFPQIRRVSMSATKALLSVNISTAYQADDQGSYPI
HVAAQAGSLAVVKLLLEWCPDCANLRDGQGRTFLHVAAEKERLALVRYVVVSSSADMILNAQ
DSNGDTPLHAAVRAGNLAVFSCLFRNRQVRLDVANQDGMTPVDLSYTRIPPRFNYSLNPRSSVR
RILLAAGAPHGGARPELFYARHIPKRDLDMEAKKHTEATQVMSIVTALIATVTFASAFTFPGGYG
PDGQPVLAGSYAFDAFILADTLAFICSISATFSLVYVGFPSMDISIRFRYLRLSAILLQSAARSLVAAF
GLGLYLLLVVHHTASAIAACAIVLASSLYGNMEFWGIIHMATTVLARIGMQRYAVVSYARKIFFT
VLRHFWSYVIIFGLPAIRKRVNSQVDKVIT
>SbANK186
MERRLLEAAMVGSATSMKEMAAQDPSLLLGTTPQGNTCLHISSIHGHEGFCKDVLTLNNSLLTV
TNMDGETPMLTAMTNGHMSLASTLLECCCTLGFSEAILQQDKNGCNALHHAIHSGHKDLALEL
IEKEPALSKAVNKYSESPMFIAVMRDFTDVSEKLLEIPGSSHVGTYGHNALHATVRNGNAVMAK
KILEGRPWLVKERTRDKKTPVLMTVIWDKIDVLRVLLEHDVFLGYECYDDAGNLSPLLVAAAYR
GHVDVAQELLNHCPDAPYCDRNGWTCLHEAVKEGQTEFVEFILRTPQLRKLINMRNNKDGRTA
LHQAVRMCNPKIVASLLFHKDTDFTLNDHITGESVIWQLRWASKHAKTLNWSLIPCFNIG

Table S3. Primer information

Primer Name	Primer Sequence (5′→3′)
PP2A-F	AACCCGCAAAACCCCAGACTA
PP2A-R	TACAGGTCGGGCTCATGGAAC
SbANK68-F	TGCATCTTGTGGAAGGGAGC
SbANK68-R	CCAGCAATGCCTTTGTGACC
SbANK69-F	TGCATCTTGTGGAAGGGAGC
SbANK69-R	CCAGCAATGCCTTTGTGACC
SbANK136-F	TCAATGGATACCGGGCAGTC
SbANK136-R	GCGTTTCCTTTGTAGGCAGC
SbANK137-F	TCAATGGATACCGGGCAGTC
SbANK137-R	GCGTTTCCTTTGTAGGCAGC
SbANK26-F	CAGGTGATGGGAAAAGGGCT
SbANK26-R	AGATCCATTGCCTCCCTTAAGAA
SbANK91-F	CATTCATAATGCCTGCGATG
SbANK91-R	CATGCAGTTGGGATTGGCAT
SbANK170-F	CCCCTCCCATGTGTCCAAG
SbANK170-R	CTTGCAGCAGAGACAAGTCG
SbANK134-F	CACCAATACATGCTCGAAGAAACA
SbANK134-R	CTAGACAACAATGGCTTGGCG
SbANK135-F	CACCAATACATGCTCGAAGAAACA
SbANK135-R	CTAGACAACAATGGCTTGGCG
SbANK127-F	TCGTCGCAGAGAGATTGCAT
SbANK127-R	TGGCGCCTGCCTCTATTTTT

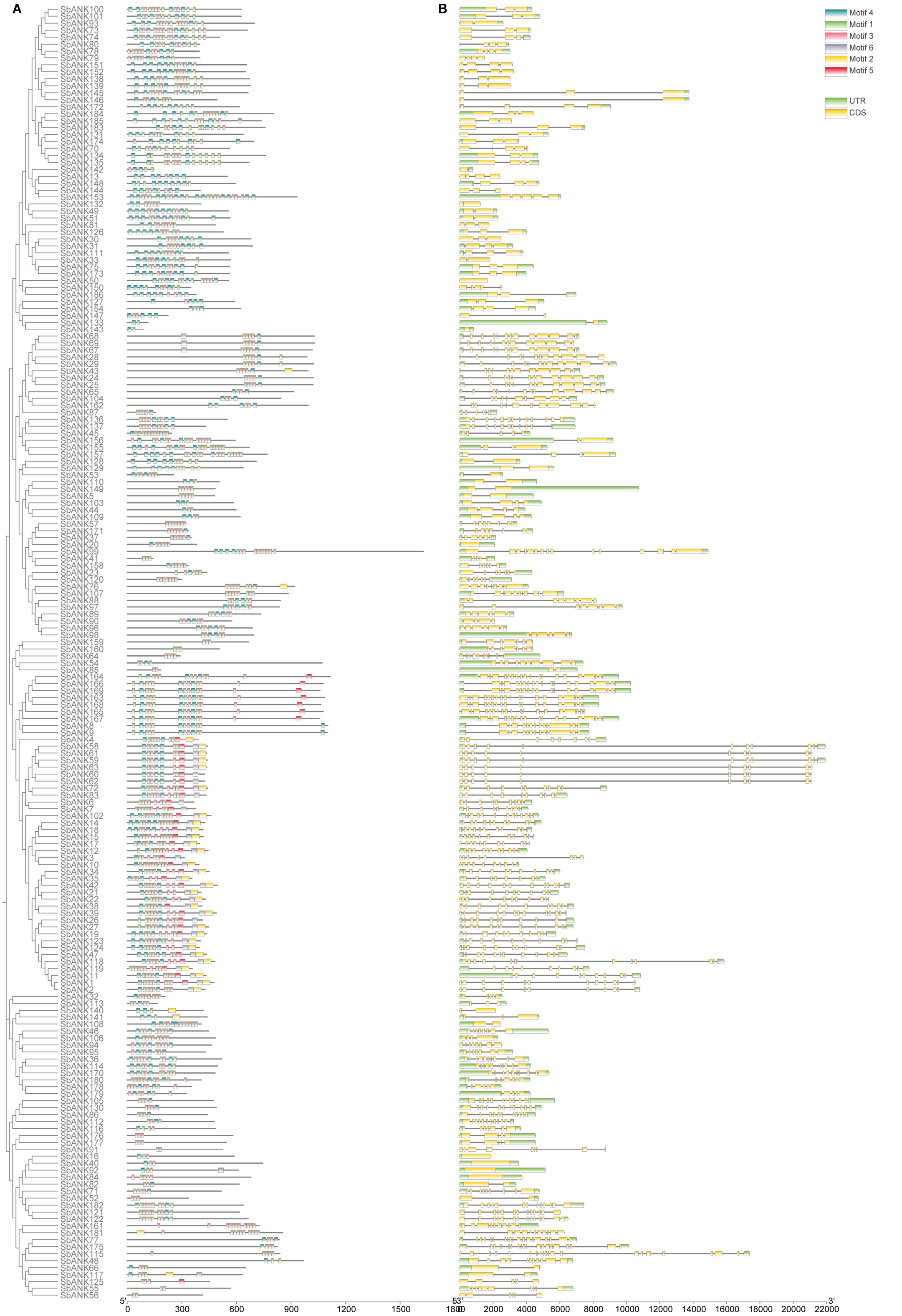

Figure S1. Detailed information on the conserved motifs and exon-intron organization of ANK candidate genes in *S. bicolor* where (A) represents conserved motifs, and (B) represents exon-intron organization.

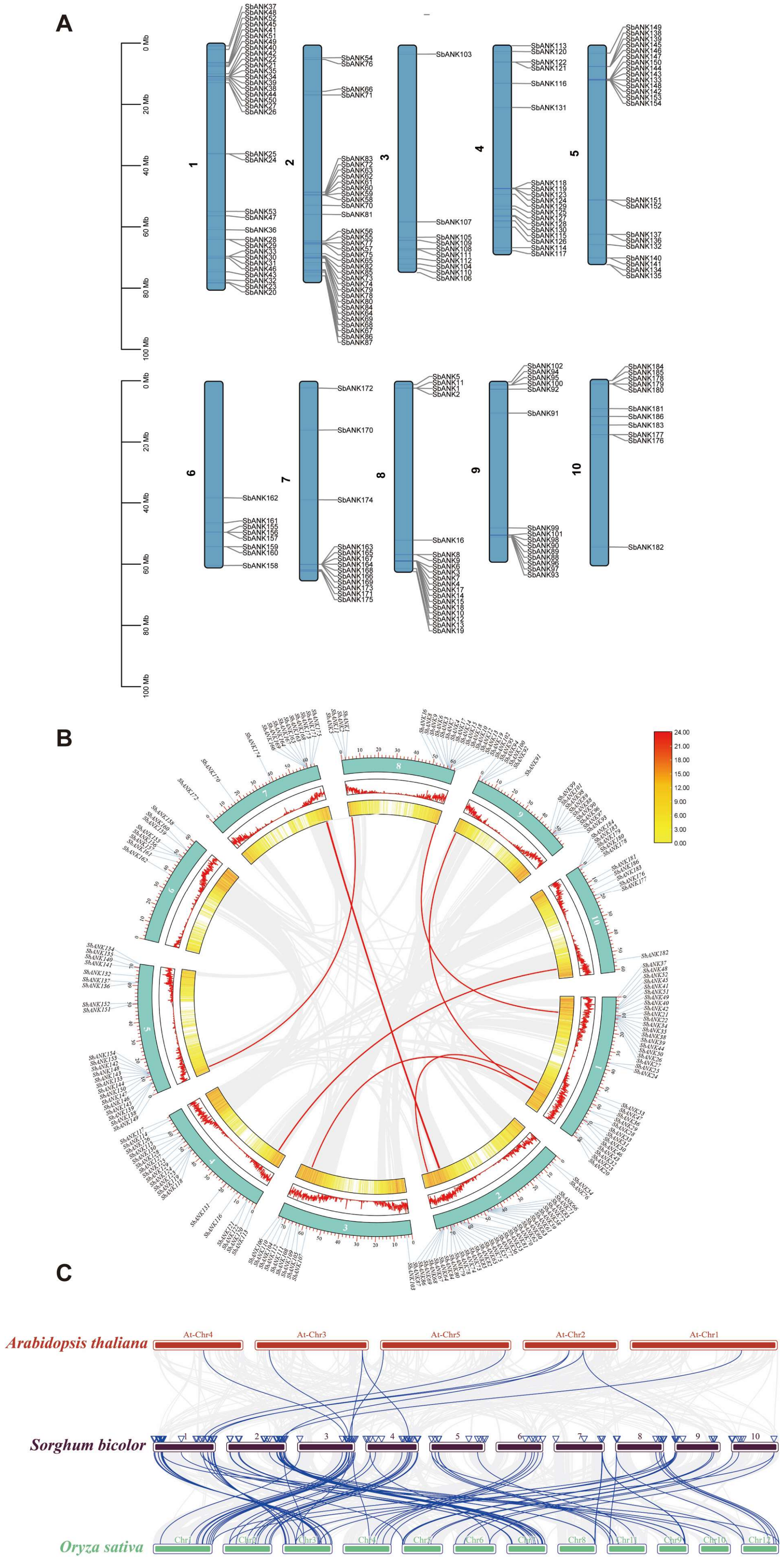
A
0 Mb
20 Mb
40 Mb
60 Mb
80 Mb
100 Mb
B
24.00
21.00
18.00
15.00
12.00
9.00
6.00
3.00
0.00
C
Arabidopsis thaliana
At-Chr4
At-Chr3
At-Chr5
At-Chr2
At-Chr1
Sorghum bicolor
Oryza sativa
Chr1
Chr8
Chr10

Figure S2. Chromosomal distribution and synteny analysis of *ANK* candidate genes in *S. bicolor*. (A) Spatial distribution of *ANK* candidate genes on the chromosomes of *S. bicolor*. (B) Intraspecies synteny analysis describing the chromosomal localization of *ANK* candidate genes. (C) Interspecies collinearity analysis illustrating the relationships of *ANK* candidate genes among *S. bicolor*, *A. thaliana*, and *O. sativa*. Colored lines represent syntenic relationships of *ANK* candidate genes between *S. bicolor* and other plant species.

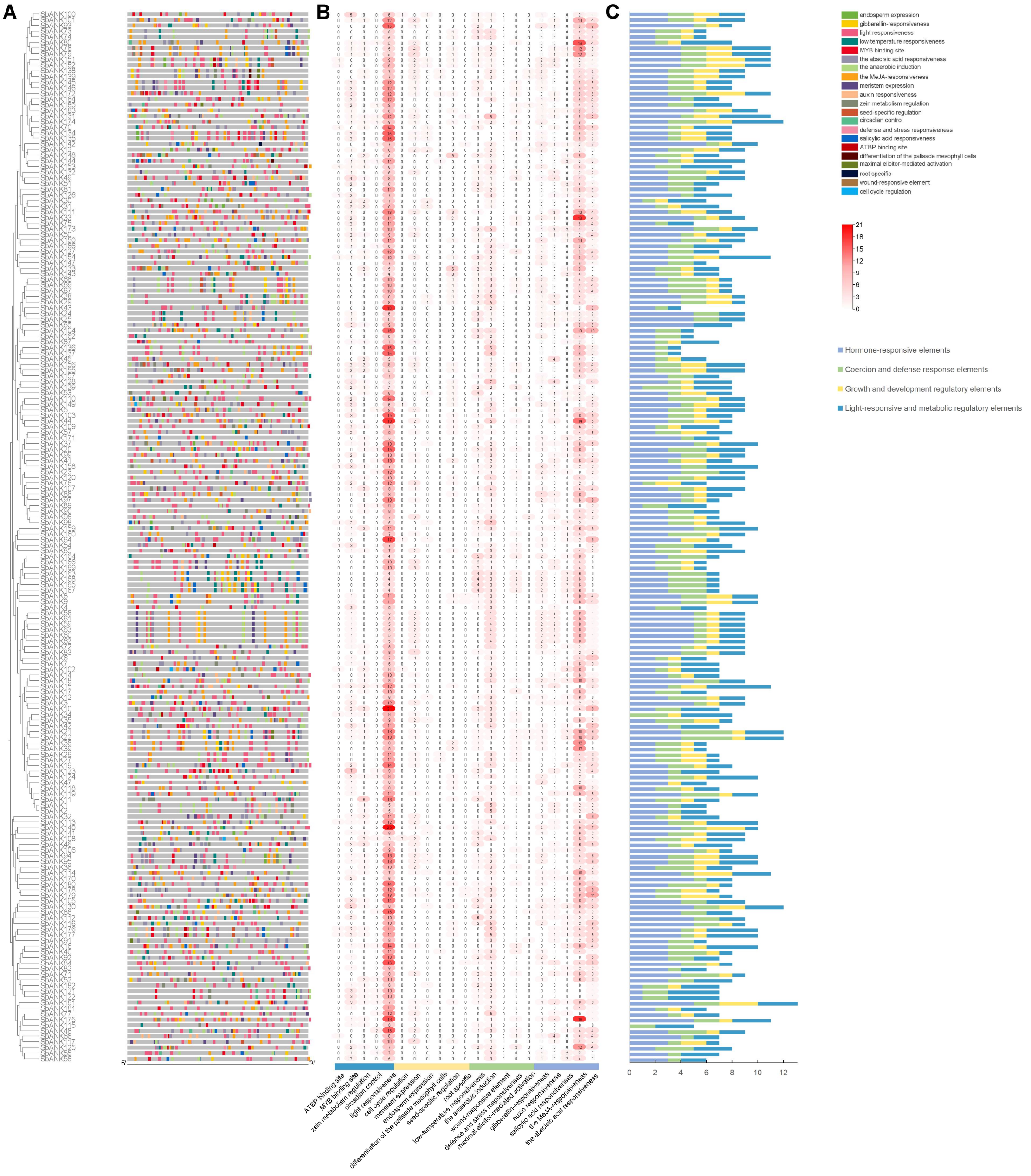

Figure S3. Quantitative analysis of the number of cis-acting regulatory elements in *SbANK* candidate genes. Different color shades and corresponding grid numbers represent the number of distinct promoter elements identified in the *SbANK* candidate genes. The multicolored histogram shows the total number of cis-acting regulatory elements in each functional category: blue represents hormone response elements, green represents coercion and defense response elements, yellow represents growth and development-related elements, and teal represents light response.
